# TETRASPANIN 8-1 from *Phaseolus vulgaris* plays a key role during mutualistic interactions

**DOI:** 10.3389/fpls.2023.1152493

**Published:** 2023-07-03

**Authors:** Thelma J. Parra-Aguilar, Luis G. Sarmiento-López, Olivia Santana, Juan Elías Olivares, Edgar Pascual-Morales, Saul Jiménez-Jiménez, Andrea Quero-Hostos, Janet Palacios-Martínez, Ana I. Chávez-Martínez, Luis Cárdenas

**Affiliations:** ^1^ Departamento de Biología Molecular de Plantas, Instituto de Biotecnología, Universidad Nacional Autónoma de México, Cuernavaca, Morelos, Mexico; ^2^ Departamento de Biotecnología Agrícola, Centro Interdisciplinario de Investigación para el Desarrollo Integral Regional Unidad Sinaloa-Instituto Politécnico Nacional, Guasave, Sinaloa, Mexico

**Keywords:** nodulation, mycorrhization, tetraspanin, nodule, arbuscule, ROS, exosomes

## Abstract

Arbuscular mycorrhizal (AM) fungi and rhizobia form two of the most important plant-microbe associations for the assimilation of phosphorus (P) and nitrogen (N). Symbiont-derived signals are able to coordinate the infection process by triggering multiple responses in the plant root, such as calcium influxes and oscillations, increased reactive oxygen species (ROS), cytoskeletal rearrangements and altered gene expression. An examination was made of the role of tetraspanins, which are transmembrane proteins that self-organize into tetraspanin web regions, where they recruit specific proteins into platforms required for signal transduction, membrane fusion, cell trafficking, and ROS generation. In plant cells, tetraspanins are scaffolding proteins associated with root radial patterning, biotic and abiotic stress responses, cell fate determination, plasmodesmata and hormonal regulation. Some plant tetraspanins, such as *Arabidopsis thaliana* TETRASPANIN 8 and TETRASPANIN 9 (AtTET8 and AtTET9) are associated with exosomes during inter-kingdom communication. In this study, a homolog of *AtTET8*, *PvTET8-1*, in common bean (*Phaseolus vulgaris L.* var. Negro Jamapa) was examined in roots during interactions with *Rhizobium tropici* and *Rhizophagus irregularis*. The promoter of *PvTET8-1* contained several *cis-*acting regulatory DNA elements potentially related to mutualistic interactions, and *PvTET8-1* was transcriptionally activated during AM fungal and rhizobial associations. Silencing it decreased the size and number of nodules, nitrogen fixation, and mycorrhizal arbuscule formation, whereas overexpressing it increased the size and number of nodules, and mycorrhizal arbuscule formation but decreased nitrogen fixation. *PvTET8-1* appears to be an important element in both of these mutualistic interactions, perhaps through its interaction with NADPH oxidase and the generation of ROS during the infection processes.

## Introduction

Arbuscular mycorrhizal (AM) symbiosis is widespread in land plants including liverworts, some of the closest living relatives when plants colonized land 500 million years ago (MYA; (Rich et al., 2021). Later, the symbiotic interaction between rhizobia and legume plants occurred. Both mutualistic interactions require a molecular dialogue that involves the exchange of specific signaling molecules. Legumes secrete particular flavonoids or strigolactones that are specifically recognized by rhizobia or AM fungi, respectively (Fisher and Long, 1992). These molecules induce the expression of specific genes, which encode proteins involved in the synthesis and secretion of Nod factors (NFs) from rhizobia, or the Myc factors from AM fungi (Oldroyd, 2013; Ferguson et al., 2019). NFs or Myc factors are lipochitin-oligosaccharides that can be recognized by specific receptors in the plant root which activate a signal pathway that induces several responses, such as ionic changes, membrane depolarization, cytoskeleton rearrangements, reactive oxygen species (ROS) generation, and altered gene expression (Denarie et al., 1996; Cardenas et al., 2000; Cárdenas and Quinto, 2008; Zepeda et al., 2014). Soon after the rhizobia reach the root hair, there are profound morphological changes that involve cytoskeleton and Ca^2+^ changes in the tip of root hair cells in a NF-dependent manner (Cárdenas et al., 1999; Miller et al., 1999; Shaw and Long, 2003; Yokota et al., 2009). Then, the plasma membrane invaginates forming a tunnel-like structure named the infection thread, which allows the bacteria to navigate through the root hair (Oldroyd, 2013). Simultaneously, cortical cells divide to form nodule primordia that the rhizobia colonize in structures named symbiosomes. Once mature, the nodule is able to fix atmospheric nitrogen (Oldroyd et al., 2011). AM symbiosis shares many of the signal pathways with nodulation. In fact, nodulation may have recruited many of the ancient molecular mechanisms developed for mycorrhizal association (Kistner and Parniske, 2002; Parniske, 2008). However, there are some differences, such as AM fungi inducing the formation of the hyphopodia and then colonizing the cortical cells, resulting in arbuscule formation (Luginbuehl and Oldroyd, 2017). Both bacterial colonization through the infection thread and AM arbuscule formation require active vesicular trafficking, endocytosis and exocytosis in order to increase the membranal surface required for symbiosome and arbuscule formation (Heck et al., 2016; Russo et al., 2019). Therefore, an efficient communication is required between the symbiont and host plant to coordinate rhizobial and AM infection processes.

Tetraspanins are widely distributed in animals, fungi, and plants, and are considered to have co-evolved with multicellular organisms (Reimann et al., 2017). In animal cells, tetraspanins are typically localized at the cell–cell interface in tetraspanin-enriched microdomains (TEMs), where they associate with each other and other membrane-bound molecules, including cholesterol, and build important molecular platforms for cell–cell interactions (Wang et al., 2012; Zimmerman et al., 2016; Cunha et al., 2018; Huang et al., 2018). Tetraspanins have four transmembrane domains with the N and C tails localized on the cytoplasmic side of the membrane. The four transmembrane domains allow for the formation of two extracellular loops, one small and one large. The large loop has highly conserved cysteine residues, which could act as redox and pH sensors or promote specific protein-protein interactions (Boavida et al., 2013; Cunha et al., 2018). The small loop in plant tetraspanins contains a cysteine residue, but this residue is absent in animal tetraspanins (Zöller, 2009). Although tetraspanins in animal cells have been involved in various biological functions, such as cell motility, morphology, signaling, plasma membrane dynamics, and protein trafficking, their role in plant cell have been described as scaffolding proteins associated with root radial patterning, biotic and abiotic stress responses, cell fate determination, plasmodesmata and hormonal regulation (Boavida et al., 2013; Mani et al., 2015; Wang et al., 2015; Reimann et al., 2017). Furthermore, tetraspanins have been described recently in plant cells as key components of exosomes, which are vesicles derived from the exocytic multivesicular bodies (MVB) that carry important molecules, such as lipids, proteins, messenger RNAs, and microRNAs. These exosomes play important roles in cell-to-cell communication in animal cells, and during pathogenic interactions in plant cells (Cai et al., 2018; Jimenez-Jimenez et al., 2019a).Recently the key roles for extracellular vessicles during mycorrhizal interactions has been elegantly addressed (Roth et al., 2019; Holland and Roth, 2023).

Tetraspanins may also be important in generating ROS. Exoskeleton development of the nematode *Caenorhabditis elegans*, requires the ROS-dependent cross-linking of tyrosine residues mediated by a NADPH oxidase, and the tetraspanin TSP-15 is involved (Edens et al., 2001; Moribe et al., 2004; Moribe et al., 2012; Moribe and Mekada, 2013). During plant infection with the pathogenic fungus *Magnaporthe grisea*, there is a requirement for ROS generation which is mediated by the fungal tetraspanin Pls1 and an NADPH oxidase (Clergeot et al., 2001; Veneault-Fourrey et al., 2005; Moribe et al., 2012; Moribe and Mekada, 2013; Siegmund et al., 2013). As tetraspanins are expressed in specialized tissues, such as the quiescent center or the early initial cells that give rise to lateral roots meristems, these proteins may also have specific tissue function or contribute to cell fate determination (Lieber et al., 2011; Boavida et al., 2013; Wang et al., 2015; Reimann et al., 2017; Yamaguchi et al., 2017a; Jimenez-Jimenez et al., 2019b). The meristematic distribution of some tetraspanins suggests that these proteins might be involved in regulating meristematic activity, which is highly dependent on ROS accumulation generated by NADPH oxidase activity, with superoxide-promoting meristematic activity and H_2_O_2_-promoting cell differentiation (Tsukagoshi et al., 2010; Zeng et al., 2017). On the other hand, during nodule and arbuscule development, there is an intensive NADPH-oxidase-derived ROS generation (Montiel et al., 2012; Arthikala et al., 2014; Montiel et al., 2016). It is possible that the molecular mechanism that maintains meristematic activity in the root is involved in nodule meristem development, and tetraspanins have similar functions during nodule development as in lateral root formation in *Arabidopsis* (Wang et al., 2015). This idea is strengthened by a recent report describing that a tetraspanin gene both regulates the auxin response in orchids to increase perianth size due to larger and more cells in the perianth, and enhances the efficiency of the auxin response to affect anther dehiscence, drought tolerance, and lateral root formation (Chen et al., 2019).

On the other hand, our knowledge for the role of tetraspanins in mutualistic interaction is limited. It is clear that mutualistic association requires a cross-talk communication between the host and AM fungi or rhizobia, and that this signaling can occur before the hypha come in contact with the host or the rhizobia start navigating the infection thread. It is unknown if TETRASPANIN 8, a component of MVB in *A. thaliana*, plays a role during these mutualistic interactions (Choi et al., 2012; Cai et al., 2018). This work describes the *Phaseolus vulgaris TETRASPANIN 8-1* (*PvTET8-1*), which we previously reported to be highly expressed in the root meristematic region and during the early stages of primordium nodule development (Jimenez-Jimenez et al., 2019b). *PvTET8-1* was highly induced during nodulation and AM symbiosis, which suggests that it plays a role during mutualistic interactions. Furthermore, the subcellular localization of *PvTET8-1* at the apical plasma membrane of *P. vulgaris* root hairs suggests that these proteins could function in polar growth, a central process during infection thread formation and rhizobia migration. Silencing and overexpression of *PvTET8-1* showed to have an effect on the number, size, and nitrogen fixation capacity of the nodules, as well as on arbuscule development. This suggests that tetraspanins contribute to the establishment of mutualistic interactions.

## Results

### *P. vulgaris* TETRASPANIN 8-1 and 8-2 grouped with *A. thaliana* TETRASPANIN 7/8/9 and increased its transcript accumulation under rhizobia inoculation

**Figure 1:**
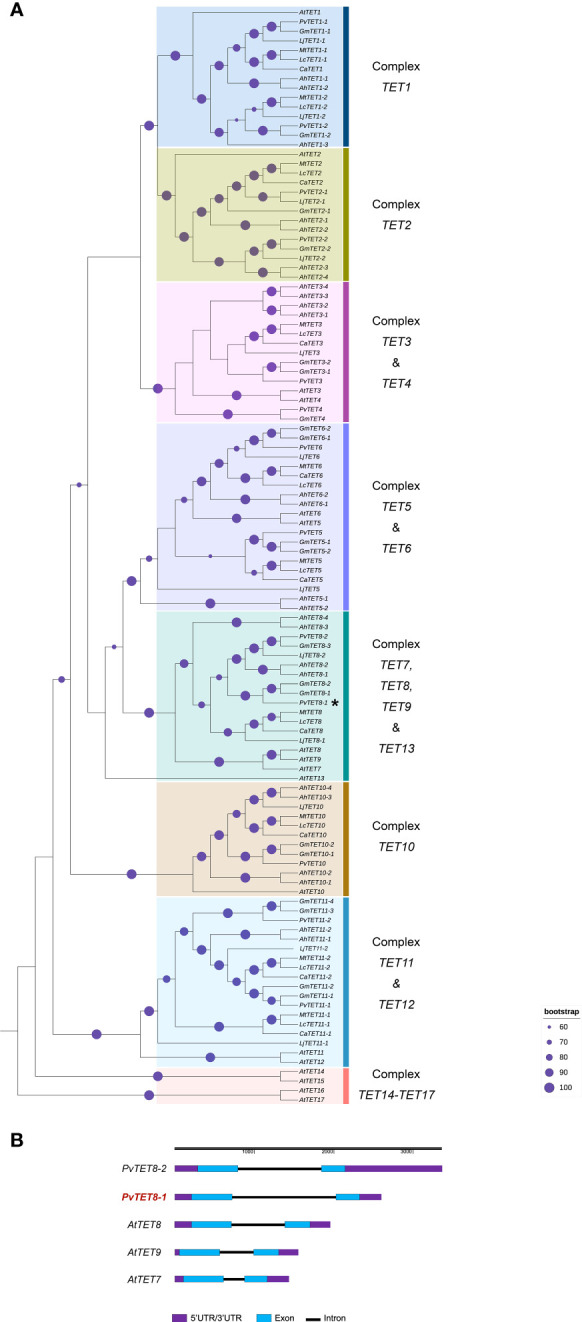
Phylogenetic analysis of tetraspanins in *A. thaliana* and seven legume species (*P. vulgaris, Glycine max*, *Arachis hypogea, Lens culinaris, Cicer arietinum, Lotus japonicus* and *Medicago truncatula)*. **(A)** The evolutionary history was inferred using 116 amino acid sequences in the Maximum likelihood method. The value of the consensus tree was (log(L) =-24190.1765) inferred using JTT+I+G4 mutation model. The identifiers of all sequences are reported in **Supplementary Table 1**. The percentage of replicate trees in which the associated taxa clustered together in the bootstrap test (1,000 replicates) are shown on the branches (purple circle). The tree is rooted on the earliest diverging TET from *A. thaliana*. The duplication events are presented in *G. max* and *A. hypogea*. Duplication events that result in the two *PvTET8* copies in *P. vulgaris* are indicated as PvTET8-1 and PvTET8-2. **(B)** Gene structure for selected members of the Arabidopsis and common bean TET family, using the online tool Gene Structure Display Server (GSDS; http://gsds.cbi.pku.edu.cn/ ). Blue boxes, exons; black lines, introns; purple boxes, untranslated regions (UTR).

To determine the number and phylogenetic relationships of the TETRASPANIN (TET) members in common bean (*P. vulgaris L*. var. Negro Jamapa), we searched the database of NCBI using BLASTP (Protein-protein BLAST) and Phytozome (https://phytozome.jgi.doe.gov/pz/portal.html) using as reference the seventeen TETs that have been described in *Arabidopsis* (Boavida et al., 2013; Mani et al., 2015; Jimenez-Jimenez et al., 2019b). Seven legume species from the Fabaceae family were included in this phylogenetic analysis; *G. max, L. japonicus, M. truncatula, C. arietinum, A. hypogea, L. culinaris* and *P. vulgaris*. In *P. vulgaris* has been identified 13 putative TET sequences (**Supplementary Figure 1**). In our analysis 8 clusters were formed and the basal complex was formed by TET14-TET17 (**Figure 1**). The tetraspanins identified in the legume group were named according to the *e-value* and the percentage of identity with respect to those identified in *A. thaliana*. Based on their highest matches, Phvul.003G151800.1 with 69% and 3.3*e-*114 with respect to AtTET8 was assigned PvTET8-1. On the other hand, Phvul.009G207500.1 with 62% identity and 3.9*e-*96 with AtTET8 was assigned PvTET8-2 (**Supplementary Figure 1** and **Supplementary Table 1**). Some duplications events are observed in cluster 10, for example in *A. hypogea* and *G. max*. Legume homologues corresponding to AtTET8 were identified in complex 7, 8, 9 and 13. In *P. vulgaris* we identified two copies homologue to AtTET8 which we named as PvTET8-1 and PvTET8*-2*. In complex 5 and 6, we identified the corresponding homologues to PvTET5 and PvTET6, respectively. Cluster 3 and 4 form a complex, and some duplication events are identified. Regarding cluster 2, some duplication events are also observed in *P. vulgaris* and *G. max*. Finally in cluster 1, AtTET1 is grouped with its corresponding homologues in legumes. In this cluster, some duplication events are also observed in six of the seven species included in this analysis (**Figure 1A**). Both those clusters in *Phaseolus* and *Arabidopsis* have the same number of exons and introns (**Figure 1** and **Supplementary Table 1**).

By using the information of temporal and spatial expression in different tissues as well as during nodulation at different stages in public transcriptome reports, we were able to identify the pattern of expression of *PvTET* genes at different nodulation stages in *P. vulgaris* (**Supplementary Figure 2A**). Interestingly, most of *PvTET* genes respond to the rhizobia infection, in which four (*PvTET1-2, PvTET5, PvTET6* and *PvTET8-1*) of the 13 genes are expressed at some stages during the nodule development. However, *PvTET8-1* is the only one that experiment a clear accumulation in all stages of the nodule development. It is noteworthy to mention that inoculation with *R. giardini*, a rhizobium able to nodulate but no fixing nitrogen (Nod+, Fix-), *PvTET8-1* experiment a highest values of transcript accumulation (**Supplementary Figures 2A, B**). These increased expression patterns are consistent with different rhizobia strain inoculation (**Supplementary Figure 2A**) and suggest a role for tetraspanins in nodule development and plant microbe interaction. Then, a close inspection of the reported expression values in the atlas of expression data base, depict that *PvTET8-1* appears to be the only one expressed during the nodule development and no detected expression for *PvTET8-2* (**Supplementary Figure 2B**). To complement the bioinformatic data, and previous data on *PvTET8-1* in *P. vulgaris* during rhizobial colonization (Jimenez-Jimenez et al., 2019b), we did a RT-qPCR to determine the *PvTET8-1* transcript accumulation during the nodulation process in *P. vulgaris* roots inoculated with *R. tropici*. We collected the infection response zone of the plant root at 3- and 5-days post inoculation (dpi) since no nodules can be observed at this time after the infection and collected the dissected nodules from 7-, 14-, 18-, 21-, 25- and 30-dpi. The transcript accumulation levels during nodule development were compared to uninoculated roots (the same equivalent region where nodules usually appear). We found that in inoculated roots *PvTET8-1* significantly increases its transcript accumulation during the nodule development, reaching the highest level at 25 dpi and start to decrease at 30 dpi (**Supplementary Figure 2C**). As a control we used *PvENOD40a* as an early nodulin and *PvLeghemoglobin* as a later nodulin to validate its expression during the nodule development. As expected, *PvENOD40a* has an earlier expression as compared to *PvLeghemoglobin* (**Supplementary Figure 2C**). Taken together, the results suggest that *PvTET8-1* is highly expressed during rhizobia colonization, cell division and nodule maturation. We observed that *PvTET8-1* transcript accumulation in uninoculated conditions also increase, however these changes were lower and never reached those levels observed in nodules (**Supplementary Figure 2C**).

### The *PvTET8-1* promoter is activated in *P. vulgaris* roots during mutualistic interactions

**Figure 2:**
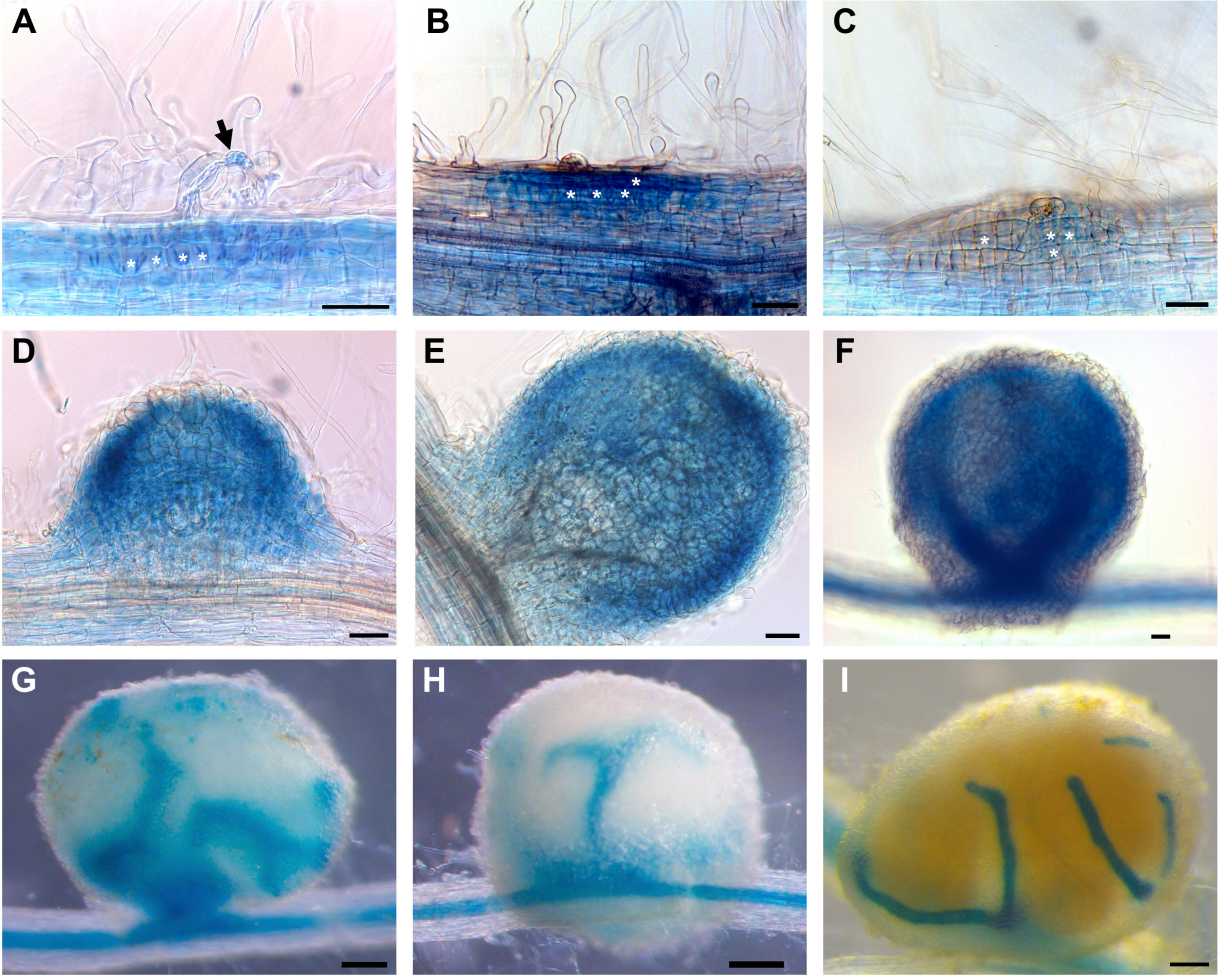
*PvTET8-1* promoter activity during nodule development in *P. vulgaris*. **(A-I)** Transgenic hairy roots expressing *pPvTET8-1::GFP-GUS* inoculated with *R. tropici*. **(A)** Infected root hair (black arrow), **(B)** curled root hair and cellular division, **(C)** nodule primordium formation at 7 dpi, **(D)** nodule meristem, **(E-H)** young nodules at 14 dpi, **(I)** mature nodule at 21 dpi. White asterisks mark cell division. Scale bars in **(A-F)** correspond to 50 μm and in **(G-I)** represent 200 μm.

An *in silico* analysis of the *PvTET8-1* promoter region of 1 kb revealed several *cis*-acting regulatory DNA elements potentially related to mutualistic interactions (**Supplementary Figure 3A**). These *cis* elements are involved in nodulation and mycorrhization, such as *NODCON1GM* (AAAGAT), *NODCON2GM* (CTCTT), which have been described in leghemoglobin genes and during arbuscule formation (Vieweg et al., 2004; Fehlberg et al., 2005). Another *cis* element is *PHO* (CACGTG), also described as a *G-Box* or *CACGTGMOTIF* that can be found in the promoters of phosphate (Pi) transporter genes in *Oryza sativa* (*OsPT4* and *OsPT11* as previously described (Hatorangan et al., 2009). Five *WRKY71OS cis* elements also known as W-Box which is a binding site for WRKY transcription factor involved in stress, Pi transporter, pathogen, and wound responses (Eulgem et al., 1999). The *ARR1AT* (NGATT) *cis* element was also found, which is important in the regulation by cytokines and Pi transporter genes (Oka et al., 2002; Hatorangan et al., 2009). For *in vivo* promoter analysis during nodulation and mycorrhization, the 1kb promoter region was fused to the green fluorescent protein (GFP) and β-glucuronidase (GUS) reporter genes. Thus, the transcriptional fusion *pPvTET8-1::GFP-GUS* was generated. The GUS histochemical analysis of composite plants expressing *pPvTET8-1::GFP-GUS* depict clear *PvTET8-1* promoter activity in the root apical meristems, lateral root meristems, and vascular bundles under non-inoculated control conditions (**Supplementary FigureS 3B-D**). However, following *R. tropici* inoculation, the promoter was highly active in the root hairs harboring the infection thread (**Figure 2A**, see arrow) as previously reported (Jimenez-Jimenez et al., 2019b). However, a close inspection depict that the promoter was highly active in the dividing cells that will later on form nodule primordia (See asterisks in **Figure 2A–C**). It was also highly active later on in the well-developed nodule (**Figures 2D–F**), and then this activity was observed in vascular bundles of older nodules (**Figures 2G–I**). No GUS signal was observed in nodule primordia and nodules of transgenic roots transformed with the empty vector pBGWFS7 (**Supplementary Figures 4A-C**).

**Figure 3:**
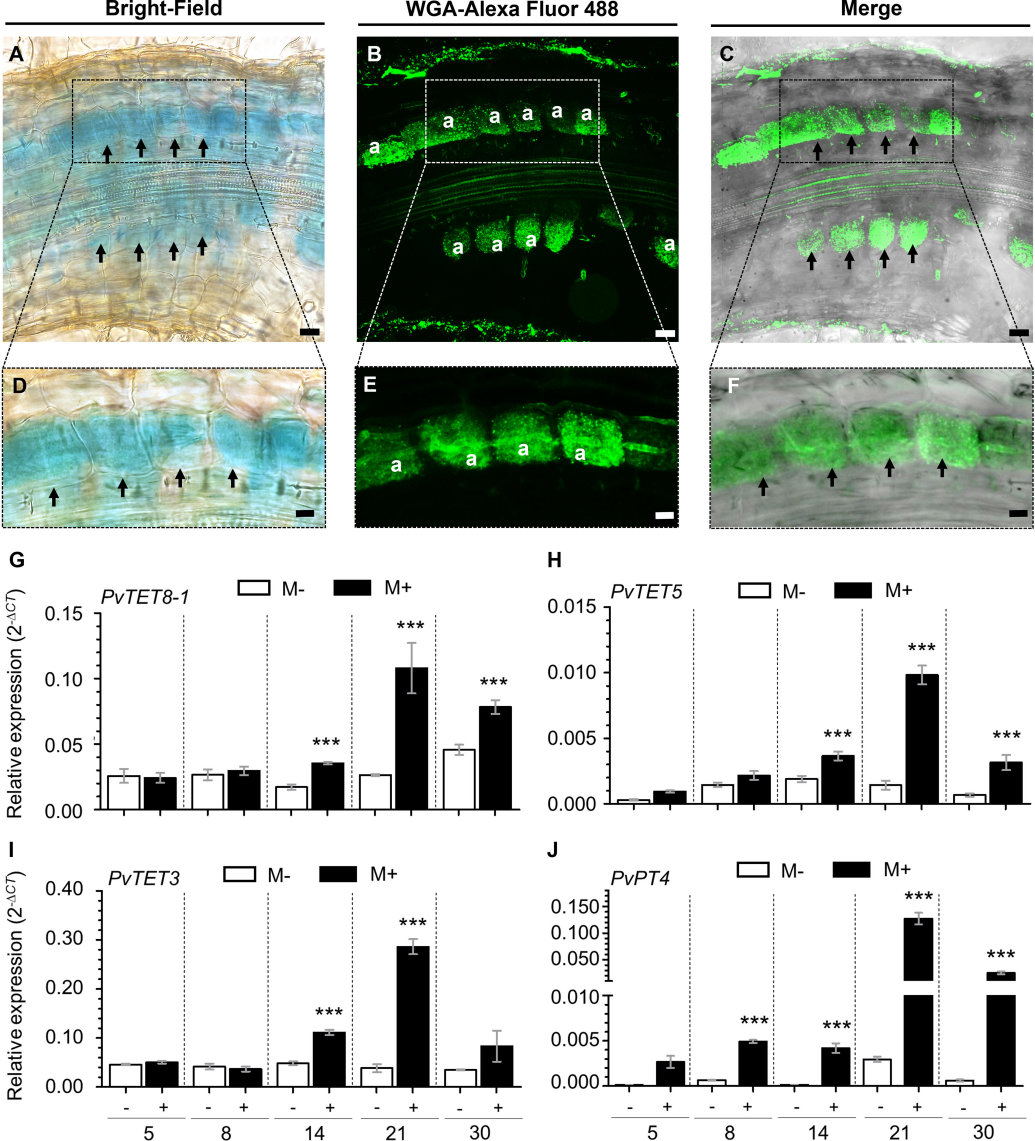
Expression profile analysis of *P. vulgaris* tetraspanins in mycorrhizal symbiosis. **(A)** Promoter activity of *PvTET8-1* visualized by histochemical staining for GUS activity in transgenic hairy roots carrying the *pPvTET8-1::GFP-GUS* construct at 14 dpi with *R. irregularis.* The GUS assay was counterstained with WGA-Alexa Fluor 488 to mark AM fungi. **(B)** WGA-Alexa Fluor 488-labeled mycorrhizal colonization of the root shown in **(A-C)** Co-localization of GUS activity (indicated by dark grey, from the confocal micrograph) with arbuscle formation (green fluorescence). **(D-F)** High magnification of **(A-C)**, respectively. Black arrows indicate cells containing arbuscules **(A)**. Scale bars: 20 μm **(A-C)** and 10 μm **(D-F)**. Relative expression of *PvTET8-1***(G)**, *PvTET5***(H)**, *PvTET3***(I)**, and *PvPT4* **(J)** in wild type *P. vulgaris* roots under mycorrhization conditions. Plants were inoculated with *R. irregularis* and the roots were evaluated by RT-qPCR at 5, 8, 14, 21 and 30 dpi. The elongation factor *PvEF1α* was used as endogenous reference gene to normalize expression levels. Bars represent mean ± standard error of the mean (SEM) of at least three independent biological replicates (*n*=3) with three technical repeats; white bars: non-inoculated roots (M-); black bars: roots inoculated with *R. irregularis* (M+). Asterisks indicate significant differences in relative expression compared with non-inoculated roots, ***: *P<*0.001 (Student’s t-test).

Composite plants expressing *pPvTET8-1::GFP-GUS* were inoculated with *Rhizophagus irregularis* and examined at 14 days post-inoculation (dpi) as it was considered to be the best time to look for arbuscules by labeling them with the fluorescent-wheat germ agglutinin conjugate (WGA-Alexa Fluor 488). This probe binds to *N-*acetylglucosamine residues, a key component of the fungal cell wall. Therefore, to visualize the AM fungi, mycorrhizal roots were stained with WGA-Alexa Fluor^®^ 488. GUS histochemical analysis showed *PvTET8-1* promoter activity in roots at 14 dpi with *R. irregularis* (**Figures 3A, D**), and arbuscule formation was observed as indicated by the fluorescent probe WGA-Alexa 488 (**Figures 3B, E**). The merged images revealed that the promoter was highly active in the region where the arbuscules were being formed indicating that *PvTET8-1* expression co-localizes with the arbuscules (**Figures 3C, F**). Transgenic roots transformed with the empty vector pBGWFS7 did not show GUS signal in cells colonized by *R. irregularis* (**Supplementary Figures 4 D-F**). RT-qPCR analysis revealed an increased accumulation of *PvTET8-1* transcripts from roots at 14 dpi with *R. irregularis* and higher accumulation at 21 dpi (**Figure 3G**). In addition to *PvTET8-1*, the expression of *PvTET5* (**Figure 3H**) and *PvTET3* (**Figure 3I**) increased under mycorrhization conditions as did the expression of *Phaseolus vulgaris Phosphate Transporter 4* (*PvPT4*), the inducible Pi transporter in arbuscule-containing cells (**Figure 3J**).

### Overexpression of *PvTET8-1* gene depict the PvTET8-1 subcellular localization and results in an increased number of larger nodules with a reduced nitrogen fixation

**Figure 4:**
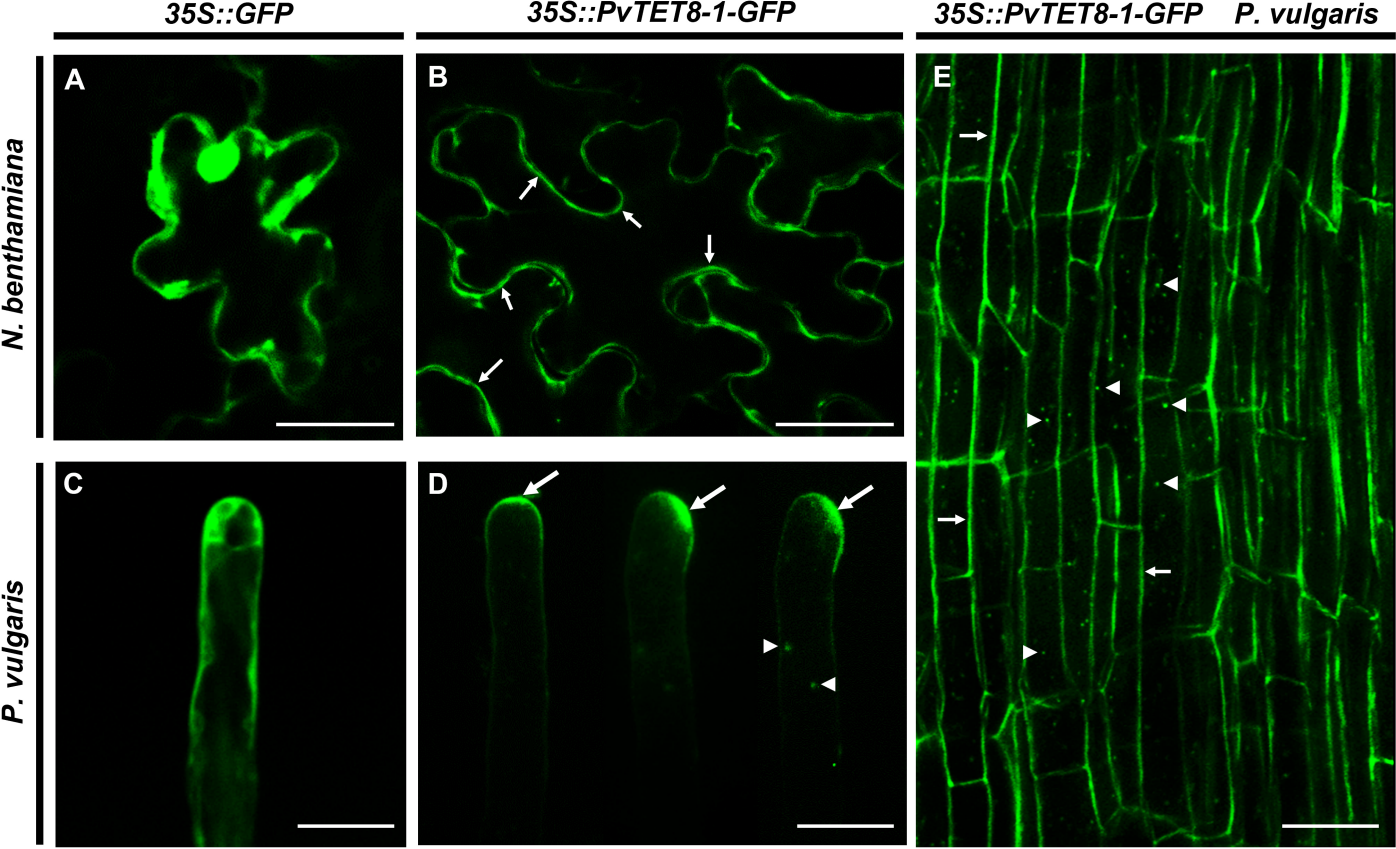
Subcellular localization of PvTET8-1-GFP in *N. benthamiana* pavement cells and *P. vulgaris* root hair and root cortical cells. **(A)** and **(C)** Subcellular localization of the control *35S::GFP* in pavement cells and root hair cells, respectively. Subcellular localization of PvTET8-1-GFP in pavement cells **(B)** and *P. vulgaris* root hair cells **(D)** and root cortical cells **(E)**. Arrows indicate the plasma membrane localization of PvTET8-1-GFP and arrowheads the localization of PvTET8-1-GFP in dynamic puncta in the cytoplasm. Scale bars correspond to 20 μm.

An overexpression of *PvTET8-1* construct was generated with the 35S promoter and the coding sequence (CDS) of *PvTET8-1* gene fused to GFP (*35S::PvTET8-1-GFP*). In transformed *Nicotiana benthamiana* pavement cells there was a clear plasma membrane localization of *PvTET8-1-GFP* (**Figure 4B**, see arrows) as compared to the control expressing the cytoplasmic GFP (**Figure 4A**). In addition, some puncta in the cytoplasm were observed which experience a clear dynamic (see Supplementary Video 1).

**Figure 5:**
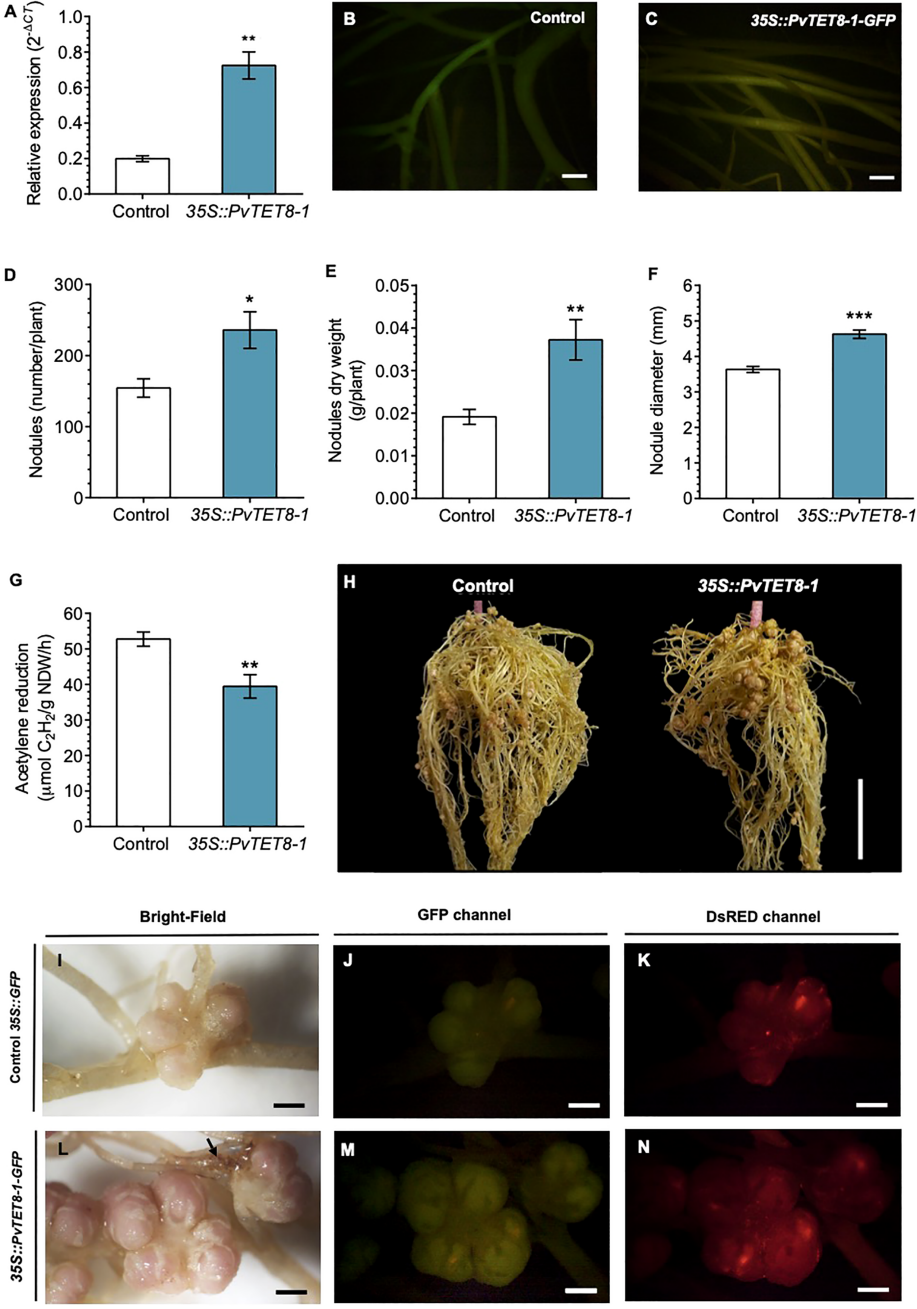
*PvTET8-1* overexpression in *P. vulgaris* generates more and larger nodules. **(A)** *PvTET8-1* relative expression in non-inoculated transgenic hairy roots carrying the control *35S::GFP* (white bar) or the *35S::PvTET8-1-GFP* construct (blue bar). Transcript accumulation data were normalized to the reference gene *PvEF1α*. Bars represent mean ± SEM of at least three independent biological replicates (composite plants, *n*=3) with three technical repeats. **(B, C)** *P. vulgaris* transgenic hairy roots expressing the control *35S::GFP* and the *35S::PvTET8-1-GFP* construct, respectively, as determined by GFP fluorescence. Quantitative analysis of number **(D)**, dry weight **(E)**, diameter **(F)** and nitrogenase activity **(G)** of nodules in *P. vulgaris* transgenic hairy roots carrying the *35S::GFP* control (white bars) and the *35S::PvTET8-1-GFP* construct (blue bars) at 21 dpi with *R. tropici*. Bars represent mean ± SEM of 10 independent biological replicates (composite plants, *n*=10). **(H)** Representative transgenic hairy roots at 21 dpi with *R. tropici*. Nodule morphology in transgenic roots expressing the control *35S::*GFP **(I-K)** and the *35S::PvTET8-1-GFP* construct **(L-N)** inoculated with *R. tropici* DsRED **(K, N)**. The black arrow points to a necrotic area. **(J, M)** Green fluorescence from transgenic hairy roots expressing GFP for selection. Data set used in panel A were analyzed by Mann-Whitney U test. The statistical analysis in panels D to G were carried out by a Student’s t-test. Asterisks indicate significant differences, *: *P<*0.05, **: *P<*0.01 and ***: *P<*0.001. Scale bars correspond to 200 μm **(B, C)**, 2 cm **(H)** and 2 mm **(I-N)**.

Expression of *35S::PvTET8-1-GFP* in *P. vulgaris* hairy roots showed that the fusion protein was localized in the apical dome of the growing root hair (**Figure 4D**, see arrows), which was different to the control cytoplasmic localization of 35S:GFP (**Figure 4C**). Note that when the root hair re-direct the polar growth to the site of the tip, the signal accumulates to the site of re-direction (**Figure 4D**). Some puncta were also observed that experienced a clear dynamic with the cytoplasmic streaming (See arrow heads in **Figure 4D** and **Supplementary Video 2**). In addition, in the root cortical cells the localization of the PvTET8-1-GFP is also very clear in the plasma membrane and cytoplasmic vesicles (**Figure 4E**, see arrows and arrow heads, respectively). A general analysis of the subcellular localization of PvTET8-1-GFP during the early stages of colonization depict a clear localization in infected cells where the infection thread is being formed (**Supplementary Figures 5A-D**). It also appears a major colonization in composite plants overexpressing the PvTET8-1 as compared to control. Even though the overexpressing composite and control plants were analyzed at the same time, note the higher colonization and cortical cell division activity under PvTET8-1 overexpression condition (**Supplementary Figure 5A-D**). Furthermore, in well-developed nodules of 14 dpi show that overexpressing condition has bigger and more colonized number of cells (**Supplementary Figure 6A, B**). As expected, hairy roots overexpressing the *35S::PvTET8-1-GFP* presented a transcript accumulation 2.6 times higher compared to the control, which expressed *35S::GFP* (**Figure 5A**). The GFP florescence from the control and *35S::PvTET8-1-GFP* overexpressing constructs (**Figures 5B, C**, respectively) were monitored to select the fluorescent transgenic hairy roots from *P. vulgaris* composite plants, which were separated from untransformed roots by removing the nonfluorescent ones.

*P. vulgaris* composite plants expressing the *35S::GFP* as a control and *35S::PvTET8-1-GFP* constructs were inoculated with *R. tropici* and evaluated the phenotype at 21 dpi. We chose this point since we have a good size to see any effect on the root nodule development such as number, size, weight and nitrogen fixation. As depicted in **Figure 5**, the overexpressing condition had a 53% increase in nodule number (**Figure 5D**), 95% higher nodule dry weight (**Figure 5E**), and 27% larger nodule diameter compared to the control (**Figure 5F**). Furthermore, nitrogenase activity was determined by the widely used acetylene reduction assay. Nitrogenase, in addition to reducing atmospheric nitrogen to ammonia can reduce acetylene to ethylene and thus constitute an indirect method to measure nitrogenase activity. In nodules overexpressing the *PvTET8-1*, acetylene reduction was decreased by 25% compared to the control, indicating a reduction in nitrogenase activity (**Figure 5G**). The phenotype of the overexpressing composite plants is depicted in **Figure 5H**, note the increased nodule number in the upper part of the root as compared to control. Nodule morphology appeared normal in the overexpressing hairy roots with pink color, but the nodules were bigger with big lenticels that appeared more well developed with some brown spots in the root that seemed necrotic (**Figure 5L**, see arrow), which were not observed in the control hairy roots (**Figure 5I**). On the other hand, the colonization degree is similar in the overexpressing condition (**Figures 5M, N**) as compared to control (**Figures 5J, K**) as depicted by colonization with *R. tropici* expressing the red fluorescent protein. Root dry weigh and stem length in the overexpressing and control composite plants at 21 dpi were not significantly different (**Supplementary Figures 7A, B**). However, leaf dry weight was 89% higher in the overexpressing compared to the control plants (**Supplementary Figures 7C, D**).

### Silencing of *PvTET8-1* gene in *P. vulgaris* reduces the nodule number and results in small nodules with reduced nitrogen fixation

**Figure 6:**
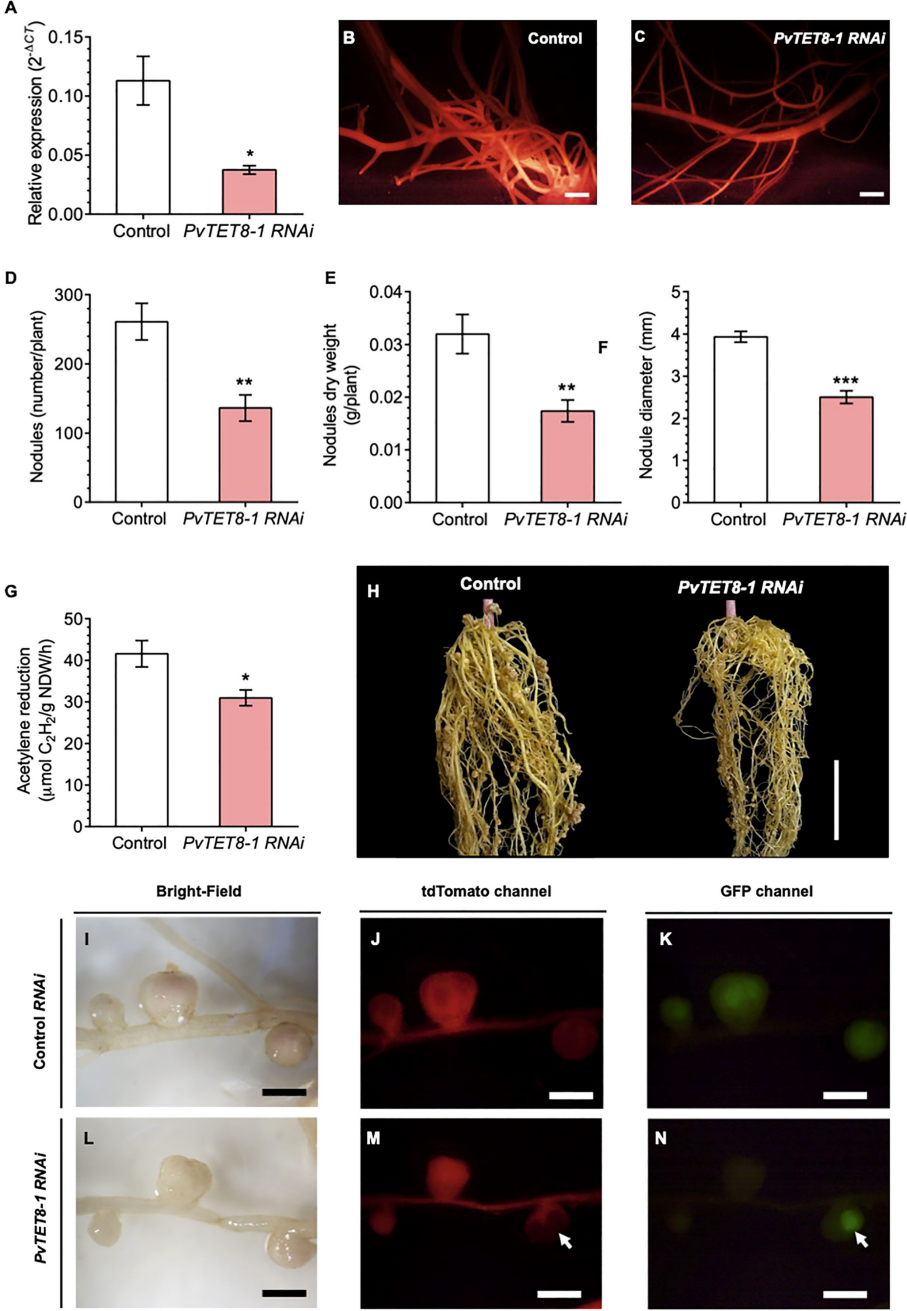
*PvTET8-1* silencing impairs *R. tropici* colonization in transgenic hairy roots of *P. vulgaris*. **(A)** *PvTET8-1* transcript accumulation in non-inoculated transgenic hairy roots expressing the *pTdT* control (white bar) and the *PvTET8-1 RNAi* construct (pink bar). All data were normalized to the reference gene *PvEF1α*. Bars represent mean ± SEM of at least three independent biological replicates (composite plants, *n*=3) with three technical repeats. **(B, C)** *P. vulgaris* transgenic hairy roots expressing the *pTdT* control and the *PvTET8-1 RNAi* construct, respectively, as determined by red fluorescence from the molecular marker tdTomato. Quantitative analysis of number **(D)**, dry weight **(E)**, diameter **(F)** and nitrogenase activity **(G)** of nodules in transgenic roots under silencing conditions at 21 dpi with *R. tropici.* Bars represent mean ± SEM of 10 independent biological replicates (composite plants, *n*=10). **(H)** Representative transgenic hairy roots expressing the *pTdT* control and the construct *PvTET8-1 RNAi* at 21 dpi with *R. tropici*. Nodule phenotype in transgenic roots carrying the *pTdT* control **(I-K)** and the *PvTET8-1 RNAi* construct **(L-N)** inoculated with *R. tropici* expressing GFP **(K, N)**. **(J, M)** Red fluorescence from transgenic hairy roots expressing the fluorescent marker tdTomato for selection. The white arrows points to a nodule that did not express the *PvTET8-1 RNAi* silencing construct and was colonized with rhizobia. Data set used in panel **(A)** were analyzed by Mann-Whitney U test. The statistical analysis in the panels **(D, G)** were carried out by a Student’s t-test. Asterisks indicate significant differences, *: *P<*0.05, **: *P<*0.01 and ***: *P<*0.001. Scale bars correspond to 200 μm **(B, C)**, 2 cm **(H)** and 2 mm **(I-N)**.

For silencing *PvTET8-1*, a *PvTET8-1 RNA interference* (*RNAi*) was created with a 231 bp fragment from the 5’UTR of *PvTET8-1* gene and transferred to *A. rhizogenes* to generate transgenic *P. vulgaris* hairy roots. Composite plants expressing the silencing construction were selected by the red fluorescence from the molecular marker tdTomato, whose gene sequence is included in the *pTdT* control and *PvTET8-1 RNAi* constructs. For the *PvTET8-1 RNAi* hairy roots, there was 67% less *PvTET8-1* transcripts compared to control hairy roots at 10 days post transformation as measured by RT-qPCR (**Figure 6A**). The decreased transcript accumulation corresponded to fluorescent red transgenic hairy roots (**Figures 6B, C**).

For the *PvTET8-1 RNAi* roots inoculated with *R. tropici*, there were 48% fewer nodules (**Figure 6D**), 46% less nodule dry weight (**Figure 6E**), 36% smaller nodule diameter (**Figure 6F**), and 25% less acetylene reduction compared to control roots (**Figure 6G**). However, there were no significant differences in root dry weight, stem length and leaf dry weight between the control and *PvTET8-1 RNAi* composite plants (**Supplementary Figures 8A–D**). An examination of the root nodules showed a clear reduction in nodule number and size (**Figure 6H**). The nodules in the silenced roots were smaller and more whitish compared with the control roots (**Figures 6I, L**). They also showed less bacterial colonization as indicated by the fluorescence of GFP–transformed *R. tropici* compared to control roots (**Figures 6J, K, M, N**). In the roots, there were some areas where part of the root was transformed and some not, and bacterial colonization drastically increased in the nodules that did not express the silencing construct (**Figures 6M, N**, see arrows).

### Overexpression of *PvTET8-1* positively regulates mycorrhizal colonization in *P. vulgaris* by increasing the number of arbuscules

**Figure 7:**
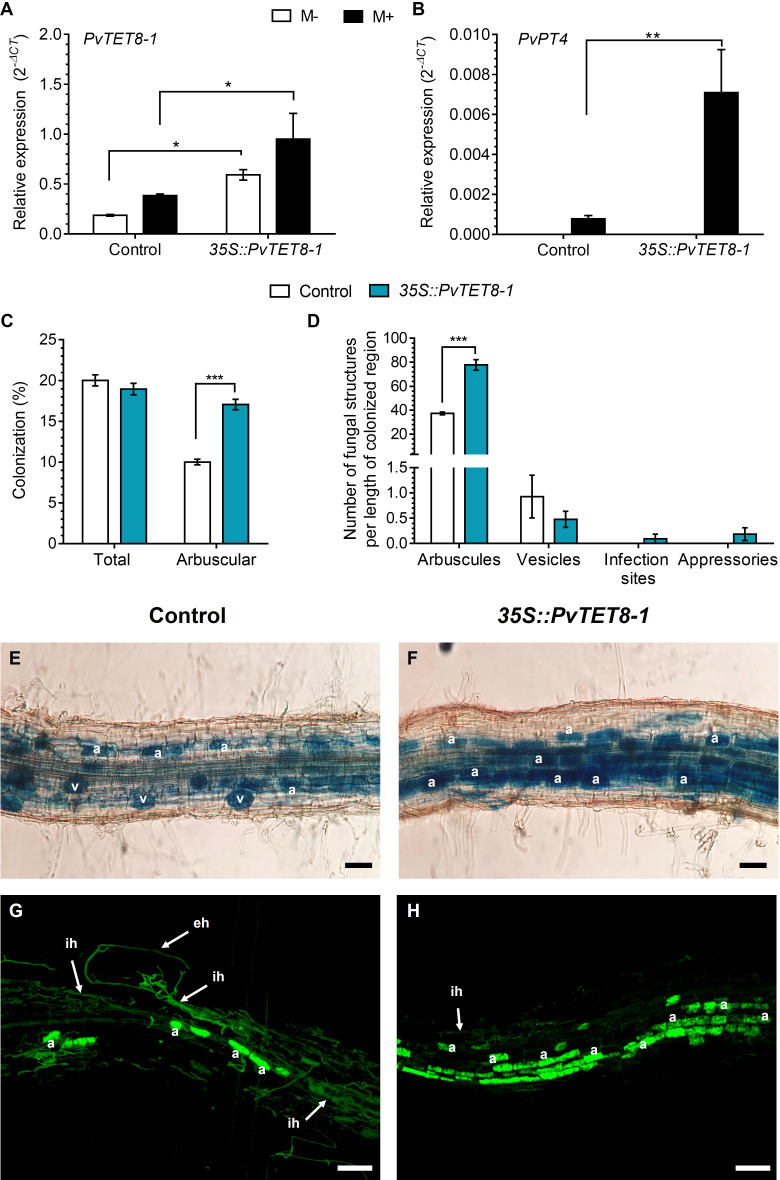
*PvTET8-1* overexpression increases mycorrhizal colonization in *P. vulgaris*. Relative expression of *PvTET8-1* **(A)** and *PvPT4* **(B)** in *P. vulgaris* transgenic hairy roots expressing the control *35S::GFP* and the *35S::PvTET8-1-GFP* construct at 30 dpi with *R. irregularis*. Transcript accumulation data were normalized to the reference gene *PvEF1α*. Bars represent mean ± SEM of at least three independent biological replicates (composite plants, *n*=3) with three technical repeats; white bars: non-inoculated roots (M-); black bars: roots inoculated with *R. irregularis* (M+). **(C)** Percentage of total colonization by measuring any AM fungal structure (vesicles, hyphae, arbuscules, etc.) and comparing with arbuscular colonization. **(D)** Quantification of AM fungal structures (arbuscules, vesicles, infection sites and appressoria). Bars represent mean ± SEM of 30 root fragments (1 cm) from six independent biological replicates (*n*=6). Data set used in panels **(A)** and **(B)** were analyzed by Mann-Whitney U test. The statistical analysis in panels **(C)** to **(D)** were carried out by a Student’s t-test. Asterisks indicate significant differences, *: *P*<0.05 and **: *P*<0.01 and ***: *P*<0.001. **(E)** and **(F)** Representative images of trypan blue-stained mycorrhizal colonization of hairy roots expressing the *35S::GFP* (control) and *35S::PvTET8-1-GFP* constructs, respectively. **(G)** and **(H)** Representative images of mycorrhizal hairy roots expressing the *35S::GFP* and *35S::PvTET8-1* constructs, respectively, stained with WGA-Alexa 488. a, arbuscule; eh, extracellular hypha; ih, intraradical hypha; v, vesicle. Scale bars correspond to 50 μm **(E, F)** and 100 μm **(G, H)**.

At 30 dpi with *R. irregularis*, the *35S::PvTET8-1-GFP* hairy roots showed an increase in transcript accumulation of *PvTET8-1* as compared to control (**Figure 7A**). In the overexpression condition, increased transcript accumulation was also detected for the mycorrhizal Pi-specific transporter, *PvPT4*, which is widely used as a marker of AM symbiosis (**Figure 7B**). As expected, in non-colonized overexpressing and control plants, *PvPT4* transcript accumulation was not detected (**Figure 7B**). In *35S::PvTET8-1-GFP* hairy roots colonized by *R. irregularis* no significant differences in total colonization were observed, interestingly, a significant increase in the number of arbuscules was observed in the overexpressing composite plants compared to the control (**Figure 7C**). However, there were no differences in the number of vesicles, appressoria or hyphal proliferation compared to the control (**Figure 7D**). There were no significant differences in root dry weight and stem length (**Supplementary Figures 9A, B**). However, leaf dry weight was significantly increased in the *35S::PvTET8-1-GFP* composite plants compared to the control (**Supplementary Figure 9C**). A representative image of each *P. vulgaris* composite plants expressing the *35S::GFP* and *35S::PvTET8-1-GFP* constructs is given (**Supplementary Figure 9D**). Mycorrhizal colonization stained with trypan blue and WGA-Alexa 488 showed an increased frequency of arbuscules in the overexpressing hairy roots (**Figures 7F, H**) compared to the control (**Figures 7E, G**).

### Silencing *PvTET8-1* expression in *P. vulgaris* results in roots with decreased mycorrhizal colonization and low *PT4* gene expression

**Figure 8:**
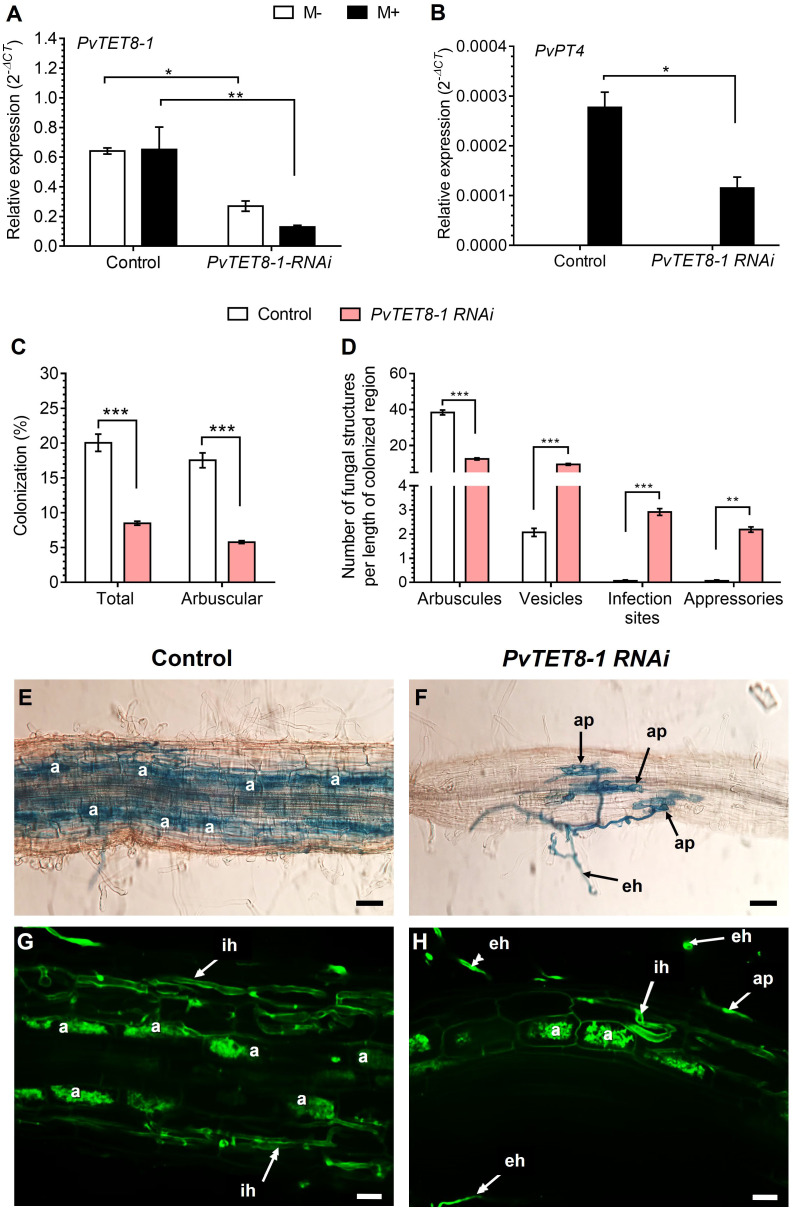
*PvTET8-1* silencing reduces mycorrhizal colonization. Relative expression of *PvTET8-1* **(A)** and *PvPT4* **(B)** in *P. vulgaris* transgenic hairy roots expressing the *pTdT* control and the *PvTET8-1 RNAi* construct at 30 dpi with *R. irregularis*. Transcript accumulation data were normalized to the reference gene *PvEF1α*. Bars represent mean ± SEM of at least three independent biological replicates (composite plants, *n*=3) with three technical repeats; white bars: non-inoculated roots (M-); black bars: roots inoculated with *R. irregularis* (M+). **(C)** Percentage of total colonization by measuring any AM fungal structure (vesicles, hyphae, arbuscules, etc.) and comparing with arbuscular colonization. **(D)** Quantification of AM fungal structures (arbuscules, vesicles, infection sites and appressoria). Bars represent mean ± SEM of 30 root fragments (1 cm) from six independent biological replicates (*n*=6). Data set used in panels **(A)** and **(B)** were analyzed by Mann-Whitney U test. The statistical analysis in panels **(C)** to **(D)** were carried out by a Student’s t-test. Asterisks indicate significant differences, *: *P*<0.05, **: *P*<0.01 and ***: *P*<0.001. **(E)** and **(F)** Representative images of trypan blue-stained mycorrhizal colonization of hairy roots expressing the *pTdT* control and *PvTET8-1 RNAi* constructs, respectively. **(G)** and **(H)** Representative images of mycorrhizal hairy roots expressing the *pTdT* control and the *PvTET8-1 RNAi* construct, respectively, stained with WGA-Alexa 488. a, arbuscule; ap, appressorium; eh, extracellular hypha; ih, intraradical hypha. Scale bars correspond to 50 μm **(E, F)** and 20 μm **(G, H)**.

The *PvTET8-1 RNAi* roots had ~70% less *PvTET8-1* transcript accumulation compared to the control (**Figure 8A**). Also, transcript accumulation of *PvPT4* was decreased 58% in the *PvTET8-1 RNAi* roots inoculated with *R. irregularis* compared to the control (**Figure 8B**). In non-colonized *PvTET8-1 RNAi* and control roots, *PvPT4* transcript accumulation was not detected (**Figure 8B**). For *P. vulgaris PvTET8-1 RNAi* hairy roots at 30 dpi with *R. irregularis*, a significant decrease of 67% occurred in total and arbuscule colonization compared to control roots (**Figure 8C**). While arbuscule formation at 30 dpi with *R. irregularis* was decreased in *PvTET8-1 RNAi* hairy roots by 67%, the formation of vesicles increased four times, infection sites increased 40 times and appressoria increased 30 times compared to control roots (**Figure 8D**). However, there were no significant differences in stem length and leaf dry weight between all conditions (**Supplementary Figure 10B, C**), except for increased root dry weight (**Supplementary Figure 10A, D**). Microscopy analysis showed a decreased arbuscule formation and massive hyphae proliferation on the surface of the *PvTET8-1 RNAi* roots (**Figures 8F, H**) compared to control roots (**Figures 8E, G**).

### Overexpression and downregulation of *PvTET8-1* affect the local level of superoxide in *P. vulgaris* hairy roots

**Figure 9:**
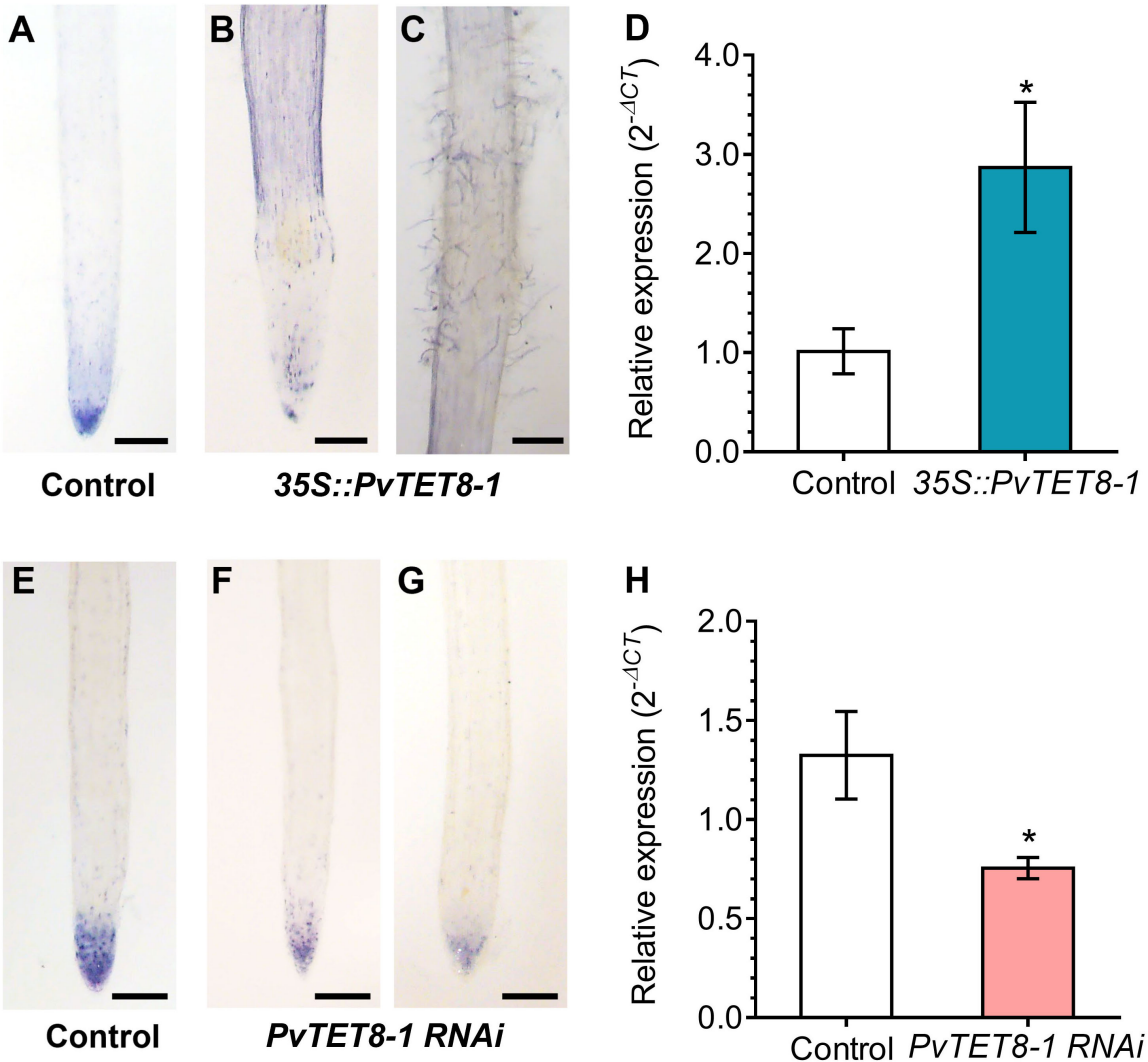
Superoxide accumulation in *P. vulgaris* transgenic hairy roots overexpressing or silencing the *PvTET8-1* gene. 15-day-old hairy roots expressing the *35S::GFP* control **(A)**, *35S::PvTET8-1-GFP* **(B, C)**, *pTdT* control **(E)** and *PvTET8-1 RNAi* **(F, G)** constructs stained with NBT. Representative images from ten independent events (transgenic hairy roots, *n*=10). **(D, H)** *RbohB* transcript accumulation in transgenic hairy roots overexpressing and silencing the *PvTET8-1* gene, respectively. Data were normalized to the reference gene *PvEF1α*. Bars represent mean ± SEM of at least three independent biological replicates (composite plants, *n*=3) with three technical repeats. Asterisks indicate significant differences, *: *P*<0.05 and **: *P*<0.01 (Mann-Whitney U test). Scale bars correspond to 200 μm.

Cellular ROS levels were evaluated based on superoxide levels detected by NBT staining. In the control hairy roots, the typical distribution of superoxide at the tip in the meristem region was observed (**Figure 9A**). However, in the *35S::PvTET8-1-GFP* hairy roots, there was less superoxide at the tip but an increase in the epidermal region at the elongating (**Figure 9B**) and root hair zone (**Figure 9C**). Transcript accumulation for *PvRbohB* also increased in the transgenic hairy roots expressing *35S::PvTET8-1-GFP* (**Figure 9D**). In the *PvTET8-1 RNAi* hairy roots, there was a decrease of superoxide in the meristem (**Figures 9F, G**) compared to the meristems of control roots (**Figure 9E**). Transcript accumulation for *PvRbohB* decreased as well in the *PvTET8-1 RNAi* roots compared to control roots (**Figure 9H**).

## Discussion

In *Arabidopsis AtTET7*, *AtTET8* and *AtTET9* form a clade, which together with the *AtTET10* clade have homologs in mosses, indicating that these are ancestral tetraspanins and already existed before the divergence of mosses and vascular plants (Wang et al., 2012). *AtTET7-9* have been described as duplicated genes, which have overlapping expression patterns, but also specific expressions in plant development and reproductive organs (Wang et al., 2012; Boavida et al., 2013; Wang et al., 2015; Reimann et al., 2017). In beans, only two homologs, *PvTET8-1* and *PvTET8-2*, clustered with *AtTET7*, *AtTET8* and *AtTET9* group of tetraspanins from *Arabidopsis*, and they all contain similar structural features in their introns/exons, suggesting a similar organization.

*PvTET8-1* expression was induced during the mutualistic interactions of nodulation and arbuscular mycorrhizal (AM) infection. The finding that *PvTET8-2* does not seem to have transcript accumulation during the mutualistic interaction, suggest that it might have another role. *PvTET8-1* is the most similar tetraspanin in beans to *AtTET8*. In *Arabidopsis*, *AtTET8* and *AtTET9* are associated with exosomes (Cai et al., 2018) and its expression is upregulated upon treatment with pathogen elicitors (Wang et al., 2015). A *TET8* homolog in wild peanut (*Arachis* spp) roots was also induced with nematodes (Guimaraes et al., 2015). The finding that *PvTET8-1* is induced when plants are infected with rhizobia and AM fungi expand its role to mutualistic interactions. One role for exosomes in *Arabidopsis* is as shuttles for several active molecules, such as lipids, proteins, messenger RNA, and microRNAs, that play important roles in cell-cell communication between *Arabidopsis* and pathogenic fungi that can silence *Botrytis cinerea* genes critical for pathogenicity (Hansen and Nielsen, 2017; Cai et al., 2018). The activity of the *PvTET8-1* promoter where arbuscules were being formed and infection thread was developing suggests that tetraspanins could play a role in mutualistic interactions as previously suggested (Holland and Roth, 2023). In silico *PvTET8-1* promoter analysis showed several regulatory elements described for Pi transporters, which may also explain why this promoter is active where arbuscules were being formed. Furthermore, the expression of *PvTET3* and *PvTET5* also increased in mycorrhizal roots showing that multiple tetraspanins are involved. These data complement the previous results regarding the *PvTET3* which shows that at 6 wpi experiment a decrease in transcript accumulation, probably anticipating the arbuscle senescence (Jimenez-Jimenez et al., 2019b). Some plant tetraspanins preferentially accumulate at the apical domain of pollen tubes and root hairs, while others are associated with cytoplasmic vesicles, suggesting a role in polar growth and cell trafficking (Berditchevski and Odintsova, 2007; Boavida et al., 2013; Jimenez-Jimenez et al., 2019a; Jimenez-Jimenez et al., 2019b). For instance, tetraspanins could be required for vesicular traffic to the root tip, and apical tetraspanin enriched membranes may recruit the molecular machinery required to maintain polar growth (Ovecka et al., 2005; Veneault-Fourrey et al., 2006; Lambou et al., 2008; Ovecka et al., 2010). The release of the rhizobia from the infection thread or the formation of arbuscule also requires a coordination with the secretory system from the host cells involving exocytosis of components needed for cell membrane remodeling, including additional peribacteroidal and periarbuscular membranes (Ivanov et al., 2012; Harrison and Ivanov, 2017; Limpens, 2019). This process could be regulated by PvTET8-1.

*PvTET8-1* overexpressing plants also increased AM colonization and increased the expression of the *PvPT4* Pi transporter indicating more efficient P assimilation. On the other hand, silencing *PvTET8-1* decreased the expression of the *PvPT4* Pi transporter, indicating less efficient P assimilation. Silencing *PvTET8-1* also decreased arbuscule development but increased the number of fungal vesicles, infection sites and appressoria. *AtTET8* in Arabidopsis plays important roles in vesicle formation and vesicle trafficking (Cai et al., 2018; Liu et al., 2020), and AM development requires considerable creation of plant membranes to form the periarbuscular membrane but also vesicles (Harrison and Ivanov, 2017; Limpens, 2019; Roth et al., 2019; Holland and Roth, 2023). Perhaps *PvTET8-1* silencing reduced arbuscule development due to poor periarbuscular membrane generation. This could reduce nutrient transport for both the plant and AM fungus, and increased vesicle formation and increased infection sites were fungal compensation mechanisms for reduce nutrient transport. However, further studies are needed to test this hypothesis. This is the first time that a tetraspanins has been shown to be involved in root mutualistic interactions for both rhizobial and AM associations.

*PvTET8-1* silencing decreases and *PvTET8-1* overexpression increases ROS accumulation in specific plant root regions, such as the elongation and root hair zone. Increased expression of *PvTET8-1* in root hairs where the infection thread forms during early cell division in cortical cells could be related to the NADPH-oxidase-mediated ROS generation that is required for meristematic activity. On the other hand, the root hairs tip region also requires NADPH oxidase activity for the generation of ROS, which regulates polar growth (Foreman et al., 2003; Takeda et al., 2008). Another example is the accumulation of tetraspanins at the site of female gametophyte differentiation, which is a ROS-dependent process (Boavida et al., 2013). Therefore, it is tempting to speculate that tetraspanins also play a role in recruiting the ROS-generating machinery to regulate growth at specific cellular locations in plant-symbiont interactions. The co-occurrence of tetraspanin and NADPH oxidase in the apical root hair cells, early infection thread and nodule primordia could be related to the need for ROS generation in those stages of the interaction (Montiel et al., 2012; Arthikala et al., 2013; Arthikala et al., 2014). Tetraspanins also regulate protein recruitment to specific tetraspanins enriched microdomains (tetraspanin web) (Hemler, 2003; Berditchevski and Odintsova, 2007; Andreu and Yanez-Mo, 2014; van Deventer et al., 2017). Thus, the link between tetraspanin and ROS could be that NADPH oxidases are among the proteins recruited by tetraspanins. In *C. elegans*, the cuticle composed of collagen is tyrosine cross-linked in a NADPH oxidase (BLI-3) generated ROSs and the participation of tetraspanin TSP-15, has been suggested to allow the recruitment of the NADPH oxidase (Edens et al., 2001; Moribe et al., 2004; Moribe et al., 2012; Moribe and Mekada, 2013). In the fungi *M. grisea* and *B. cinerea*, a tetraspanin PLS1 drives the plant infection to reestablish appressorium polarity (Clergeot et al., 2001; Veneault-Fourrey et al., 2005; Lambou et al., 2008; Moribe et al., 2012; Moribe and Mekada, 2013; Siegmund et al., 2013). Therefore, tetraspanins and the ROS-generating machinery seems to be connected, and this work indicates a connection in plant-symbiont interactions.

*PvTET8-1* overexpression increases the nodule size can be related to the role of tetraspanins with plant hormones controlling growth and development. *AtTET9* from the TET7/8/9 group interacted with auxin and jasmonic acid to regulate the size of orchid flowers organs and other developmental processes (Chen et al., 2019). The flower organs with higher expression of this tetraspanin were larger, and the authors suggested that it controlled cell division in the meristem or cell expansion of the flower. Furthermore, when *AtTET9* was ectopically expressed in *Arabidopsis* under the 35S promoter, the flowers and seeds show increased size by increasing the efficiency of the auxin response (Chen et al., 2019). Nodulation also involves substantial crosstalk between NFs and auxin signaling. For instance, in *Medicago truncatula*, there is a high accumulation of auxin at the site of nodule meristem formation (Herrbach et al., 2017). NFs and auxin induce two tetraspanin genes with homology to *PvTET3* (Medtr4g061010) and *PvTET1A* (Medtr8g101600; (Herrbach et al., 2017). Furthermore, *PIN1* expression is reduced in the *Arabidopsis trn2-1*(*AtTET1*) mutant, which has compromised auxin transport activity during the transition from floral meristem termination to gynoecium development (Yamaguchi et al., 2017b). In addition to altering auxin transport, biosynthesis, and homeostasis, NFs could modulate the expression of specific tetraspanins that influence hormone levels, linking tetraspanin and auxin homeostasis during cell division (Mathesius et al., 1998; Finet and Jaillais, 2012; Larrainzar et al., 2015). Therefore, NFs signaling has a profound impact on *TETRASPANIN* genes expression by affecting auxin levels and coordinating nodule primordium or arbuscule development (Jimenez-Jimenez et al., 2019b). In *Arabidopsis* and *P. vulgaris*, lateral roots formation is mediated by changes in hormone levels (Malamy and Benfey, 1997; Dubrovsky et al., 2008), and overexpression of *AtTET9* has a profound effect on the number and early lateral root formation, which could be explained by increased sensitivity to auxin (Chen et al., 2019).

The overexpression of *PvTET8-1* in *P. vulgaris* hairy roots increased number of nodules with a higher weight, larger diameter, but decreased nitrogen fixation. Silencing *PvTET8-1* decreased the number of nodules and nodule size, but also decreased nitrogen fixation similar to the overexpressing condition. These results points to a role in regulating several aspects of nodule development and nitrogen fixation. It may be that there is an optimal range of *PvTET8-1* expression, and either increased or decreased levels will somehow disrupt nitrogen fixation. Necrotic areas on several nodules were observed with the overexpressing *PvTET8-1* roots, which could indicate premature senescence, at least partially explaining why nitrogen fixation was decreased. Increased nodule weight and size in the overexpressing transgenic roots could be a consequence of the reduced nitrogen fixation. Previous studies have shown that reduced nitrogen fixation in nodules can stimulate nodule expansion *via* a systemic signal acting as a compensatory mechanism to increase nitrogen fixation (Jeudy et al., 2010; Laguerre et al., 2012). However, more work is needed to determine if this or another mechanism can explain the reduced nitrogen fixation. The larger nodules in the *PvTET8-1* overexpressing roots also had larger and more developed lenticels. These structures allow air exchange regulating permeability of nodules and appear as white stripes on the nodule surface usually flanking or near vascular bundles. Under low oxygen or water-logged conditions, they develop more extensively, whereas they collapse or develop very little with insufficient water or high oxygen pressure. Because lenticel development on the nodule surface is accompanied with the nodule vascular bundle, growth regulators supplied from the vascular system, such as auxin, will likely facilitate lenticel development. This also supports a strong connection between tetraspanins, auxin and lenticel formation (James and Crawford, 1998; Takanashi et al., 2011).

Tetraspanins have several transmembrane domains, although their subcellular localization can be diverse. PvTET8-1 was observed in the plasma membrane when transiently expressed in *N. benthamiana* pavement cells, although some puncta were also observed in the cytoplasm. In *P. vulgaris* hairy roots, PvTET8-1 subcellular localization was at the apical dome of growing root hair cells, but also with some cytoplasmic puncta similar to those found in *N. benthamiana* pavement cells. These puncta are dynamic structures whose size does not correspond to typical vesicles, and thus could be more related to larger structures, such as small vacuoles or multivesicular bodies. In addition, the subcellular localization of PvTET8-1 is at the tip of the growing root hair, which can be reorganized to the side when lateral growth occurs, suggest a connection with coordination of apical growth. However, more study is required to determine the nature of the localization of PvTET8-1 and its impact on its functions. Therefore, in summary tetraspanin PvTET8-1 specifically induced during nodule development and Mycorrhizal interactions seems to be involve in regulation of ROS-generating machinery to regulate plant-symbiont interactions.

## Materials and methods

### Phylogenetic and gene structure analysis

For phylogenetic analysis, the 17 amino acid sequences of AtTET were used as reference to identify the sequences of tetraspanins in seven species of the Fabaceae family (*P. vulgaris, L. japonicus, G. max, M. truncatula, L. culinaris, C. arietinum, and A. hypogea*) (**Supplementary Figure 1**; **Supplementary Table 1**). All sequences were downloaded from Phytozome data base (https://phytozome.jgi.doe.gov/pz/portal.html). Characteristic domains and motifs of TET sequences were identified in Meme Suite (https://meme-suite.org/meme/) and Interpro-Pfam (https://www.ebi.ac.uk/interpro/). Alignment of the 116 amino acid sequences was performed with MUSCLE in JALVIEW. Then, the analysis of phylogenetic relationships between sequences was performed with IQ-TREE (http://www.iqtree.org) using the maximum likelihood method with 1000 bootstraps. The consensus tree was edited with ITOL (https://itol.embl.de).

Exon–intron structure information for *TETRASPANIN* genes from *A. thaliana* and *P. vulgaris* were obtained using Phytozome data base (https://phytozome.jgi.doe.gov/pz/portal.html). A gene structure schematic diagram was made using Gene Structure Display Server (GSDS; http://gsds.cbi.pku.edu.cn/).

### *In silico* analysis

To determine the expression of tetraspanins under nodulation conditions, the transcriptional profiles of the databases of different tissues and developmental stages of *P. vulgaris* were analyzed. Six stages of nodulation development were considered: 0 dpi (Dalla Via et al., 2015), 5 dpi (O’Rourke et al., 2014), 7 dpi (Fonseca-Garcia et al., 2019), 15 dpi (Nanjareddy et al., 2017), 21 dpi (O’Rourke et al., 2014) and 41 dpi (Silva et al., 2019). Bioinformatics analysis was performed with scripts designed in Unix, Python, and R. Raw data was downloaded. Subsequently, they were aligned with respect to the reference genome of *Phaseolus vulgaris v2.1* (Phytozome), with Bowtie2. Total transcript counts within each condition were performed with eXpress. From these expression lists, the differential expression analysis was carried out on the IDEAMex web server (Integrative Differential Expression Analysis for Multiple Experiments; Jimenez-Jacinto et al, 2019) of the University Unit of Mass Sequencing and Bioinformatics of the Biotechnology Institute-UNAM (Jimenez-Jacinto et al., 2019). Differential expression analysis was performed with four statistical methods included in the Bioconductor platform package: NOISeq, Limma, DESeq, and EdgeR. The statistical parameters of p-value 0.05, FDR: 0.05 and CPM=1 were used to determine the differential expression of the transcripts of each tissue and stage of development. The heatmap graphic was designed in R (unpublished data).

The PLACE database (Higo et al., 1999) was used to analyze cis-acting regulatory DNA elements in the *PvTET8-1* promoter. A 1000 bp fragment upstream of the initiation codon of *PvTET8-1* were used (Jimenez-Jimenez et al., 2019b).

### Seed germination

Seeds of *P. vulgaris* L. cv. Negro Jamapa were surface sterilized with sodium hypochlorite (25%) for 5 min, rinsed five times with sterile water, treated with 95% ethanol for 1 min and rinsed another five times with sterile water (Estrada-Navarrete et al., 2007). Surface sterilized bean seeds were transferred to sterile steel plates lined with wet paper towels with Fahraeus nutrient solution (Fahraeus, 1957), covered with aluminum foil and incubated at 28°C for 2 days under non-light conditions and 30% humidity.

### Vector construction and composite plants

For *PvTET8-1* promoter analysis, *pPvTET8-1::GFP-GUS* was used (Jimenez-Jimenez et al., 2019b). The empty vector pBGWFS7 was used as a control. For overexpression of *PvTET8-1*, the open reading frame was amplified from *P. vulgaris* cDNA using the primers listed in **Supplementary Figure 11** and cloned into the pENTR™/SD/D-TOPO vector (Invitrogen^®^). The Gateway LR reaction was performed between an entry vector (pENTR/SD/D-TOPO-*PvTET8-1*) according to the manufacturer’s instructions (Invitrogen^®^) and inserted into the pH7FWG2 binary vector under the control of the constitutive 35S promoter. The empty vector pH7FWG2, which constitutively expresses GFP, was used as the control. In each step, the presence of the insert was confirmed by Sanger sequencing and PCR. To make the RNAi construct, a 231 bp fragment corresponding to the 3′-untranslated regions of *PvTET8-1* was amplified from *P. vulgaris* cDNA using the primers listed in **Supplementary Figure 11**. The PCR product was cloned into pENTR/D-TOPO vector (Invitrogen^®^). The recombination into the destination vector pTdT-DC-RNAi (Valdes-Lopez et al., 2008) was performed with the LR clonase with the Gateway System (Invitrogen^®^). The appropriate orientation of the insert was confirmed by PCR and Sanger sequencing. As a control, a truncated and irrelevant sequence from *A. thaliana* pre-mir159 (ACAGTTTGCTTATGTCGGATCCATAATATATTTGACAAGATACTTTGTTTTTCG ATAGATCTTGATCTGACGATGGAAGTAGAGCTCTACATCCCGGGTCA), was cloned into the pTdT-DC-RNAi vector and was named *pTdT* control (Montiel et al., 2012). All plasmids were introduced by electroporation into *A. rhizogenes* strain K599 to transform *P. vulgaris* cv. Negro Jamapa as described (Estrada-Navarrete et al., 2007). Transgenic hairy roots from composite plants were observed under epifluorescence microscopy to confirm the presence of the reporter gene (GFP or tdTomato), and non-transformed roots were removed. *Agrobacterium tumefaciens* strain CV3010 was used for transient expression in *Nicotiana benthamiana* leaves.

### Rhizobia inoculation

*Rhizobium tropici* CIAT899 bacteria were grown in 100 mL PY broth supplemented with 7mM CaCl_2_, 50 μg mL^-1^ rifampicin, and 20 μg mL^-1^ nalidixic acid. The broths were incubated at 30˚C with shaking at 250 rpm until the suspension reached an OD600 of 0.8.

For the nodulation assay, bean plants were grown in sterilized vermiculite and inoculated with 1 mL of *R. tropici* suspension at OD600 0.05 in 10 mM MgSO_4_. Plants were grown in a controlled environment chamber (30% humidity, 16 h light/8 h darkness, at 28˚C) and watered twice per week with Fahraeus nutrient solution (Fahraeus, 1957) without nitrate potassium concentration. At 21 days post inoculation (dpi) non-fluorescent roots were removed, and only transgenic hairy roots were evaluated; plant height, root dry weight and leaf dry weight were assessed. Nodule dry weight, number per plant and length were also determined. The nodule diameter was determined by image analysis using ImageJ editing software from micrographs obtained from *PvTET8-1* overexpressing and silenced composite plants. Ten composite plants per condition were evaluated, and two independent experiments were performed.

### Acetylene reduction assay

Acetylene reduction assay was used to quantify the nitrogenase activity. At 21 dpi non-fluorescent roots were removed, and only transgenic hairy roots were evaluated. Nodulated roots were transferred in 100 mL vials with rubber seal stoppers, which were injected with acetylene to a final concentration of 2% of the gas phase. After 60 min at room temperature, ethylene production was determined by gas chromatography (Variant, model 3300; (Ortega-Ortega et al., 2020). Specific activity was expressed as μmol ethylene^-1^ (g nodules dry weight)^-1^·h^-1^.

### *Rhizophagus irregularis* inoculation

*R. irregularis* inoculum was provided by Dra. Dora Trejo Aguilar from Facultad de Ciencias Agrícolas de la Universidad Veracruzana, Veracruz, México. Composite plants were grown in sterile vermiculite and inoculated with 140 spores of *R. irregularis* (M+) that were then homogeneously distributed in the substrate. Controls were non-colonized (M-) plants. Plants were watered twice per week with 30 mL of half-strength Hoagland nutrient solution pH 6.1 (Hougland and Arnon, 1950) containing a low concentration of potassium phosphate (10 µM, K_2_HPO_4_) to favor AM colonization. Plants were grown in a controlled environment chamber at 28°C with a 16 h light/8 h dark photoperiod and 30% humidity for 30 dpi. Plant length, root dry weight and leaf dry weight were measured. Roots were also separated longitudinally into three sections, one for determination of mycorrhizal colonization, another for histochemical staining and another stored in liquid nitrogen for RNA extraction. Ten composite plants per condition were evaluated.

### Staining and quantification of mycorrhizal colonization

*P. vulgaris* transgenic root segments were stained with 0.05% trypan blue in lactoglycerol (Phillips and Hayman, 1970) and observed by inverted microscopy (Nikon TE300) at 10-40X magnification. Total mycorrhizal colonization (intraradical hyphae, vesicles and arbuscules) was calculated according to the line-intersection method (Giovannetti and Mosse, 1980). For each plant, 30 root segments were assessed, and six plants per condition were evaluated. The arbuscular percentage was calculated with MycoCalc software (https://www2.dijon.inrae.fr/mychintec/Mycocalcprg/download.html). The number of infection sites, appressoria, vesicles and arbuscules along the length of each root was determined by image analysis using ImageJ software (ImageJ, version 1.8.0_112) as previously described (Sarmiento-Lopez et al., 2020). For each plant, 30 root segments colonized by *R. irregularis* were assessed. WGA-Alexa Fluor^®^ binds to *N-*acetylglucosamine residues, key component of the fungal cell wall. Therefore, to visualize the AM colonization, mycorrhizal roots were stained with WGA-Alexa Fluor^®^ 488 (Invitrogen^®^) according to Javot et al. (2007) and viewed using confocal laser scanning microscopy (Nikon Eclipse Ti-E). WGA-Alexa Fluor ^®^ 488 (green channel) was excited with an argon ion laser (488 nm), and emitted fluorescence was collected from 521 nm (Javot et al., 2007).

### Promoter activity analysis

Composite plants harboring *pPvTET8-1::GFP-GUS* and empty vector control were collected after 7, 14 and 21 dpi for nodulation and 14 dpi for mycorrhization assays. Histochemical staining for GUS activity was done as described by Jefferson et al. (1987) and images were acquired with an inverted microscope (Nikon Eclipse Ti-E) at 10-40X magnification (Jefferson et al., 1987). For the mycorrhization assay, dual GUS-WGA Alexa Fluor^®^ 488 staining was performed for promoter activity and mycorrhizal colonization, respectively (Kuhn et al., 2010). Colonized roots were fixed with 50% EtOH for 24 h, and then cleared in 20% KOH for 72 h. The fungal cell walls were stained with 0.2 µg/mL WGA-Alexa Fluor^®^ 488 (Invitrogen^®^) according to Manck-Gotzenberger and Requena (2016). For each construct, at least 30 root segments were analyzed (Manck-Gotzenberger and Requena, 2016).

### RT-qPCR assays

Total RNA was isolated from roots using TRIzol reagent (Invitrogen^®^) following the manufacturer´s protocol. To eliminate contaminating genomic DNA, total RNA samples (1 μg in 20 μL) were treated with 1unit DNaseI (RNase-free; Invitrogen^®^) at 37˚C for 30 min and then at 65˚C for 10 min. Two-step RT-qPCR was performed using Maxima SYBR Green/ROX qPCR Master Mix (Thermo Fisher) and quantified on real time PCR thermal cycler (QuantStudioTM 5 System). Each reaction contained 100 ng cDNA as template in 10 μL final volume. Gene-specific primers used in RT-qPCR reactions are showed in **Supplementary Figure 11**. RT-qPCR was performed at 95 °C for 10 min and 40 cycles at 95°C for 15 s and 60°C for 60 s. Primer specificity was verified by regular PCR and melting curve analysis. *P*. *vulgaris* elongation factor 1-α (*PvEF1α*, **Supplementary Figure 11**) was used as a reference gene for normalization, and the quantitative results were evaluated by the 2*^-ΔCT^* method described by Livak & Schmittgen (2001) as previously described (Livak and Schmittgen, 2001). RT-qPCR data are averages of three biological replicates with three technical replicates and two independent experiments were performed.

### Subcellular localization analysis of PvTET8-1 in *Nicotiana benthamiana* and *P. vulgaris*

The *35S:PvTET8-1-GFP* construct was electroporated into *A. tumefaciens* strain CV3010. Preparing *A. tumefaciens* (OD_600_ 0.5) for infiltration into *N. benthamiana* epidermal cells were done on leaves from 4- to 6-week-old wild type plants as previously described (Norkunas et al., 2018). Leaves transformed with the vector harboring the *35S::GFP* was used as the control. Images of transiently infected leaves were observed using confocal laser scanning microscopy (Nikon Eclipse Ti-E) after 72 h of infiltration. The subcellular localization of *PvTET8-1* was determined in *P. vulgaris* root hairs from 4-day-old transgenic hairy roots, generated with *A. rhizogenes* carrying *35S::PvTET8-1-GFP.* Root hairs were observed using confocal laser scanning microscopy (Nikon Eclipse Ti-E).

### ROS determination

Hairy roots were grown in glass tubes (15 cm) containing Fahraeus nutrient solution for 15 days. *In situ* O_2_^-^ was estimated using the nitroblue tetrazolium (NBT) staining method (Montiel et al., 2012). Samples were incubated in 50 mM NaH_2_PO_4_ (pH 7.5) with 1% NBT for 1 h in darkness at room temperature, then roots were cleared in 96% ethanol for 1 h and rehydrated (40 to 10% ethanol). Roots were placed in a 50% glycerol solution, and the presence of the insoluble blue formazan precipitate was examined in images from a stereomicroscope (Olympus SZX7, Germany).

### Statistical analysis

Data were processed to obtain the central tendency and measures of dispersion (means and standard deviations) and were examined for normal distributions using Shapiro-Wilk’s test. Comparative analyses were carried out using Student’s t-test, Mann-Whitney U test and ANOVA *post-hoc* Tukey test. Statistically significant differences are represented by the number of asterisks. Simple (*) *P*< 0.05, double (**) *P*<0.01 and triple (***) *P*<0.001. All statistical analyses were performed using the software GraphPad Prism version 6.00 for Windows (GraphPad Software).

## Data availability statement

The original contributions presented in the study are included in the article/**Supplementary Material**. Further inquiries can be directed to the corresponding author.

## Author contributions

TP-A, EP-M, LS-L and LC generated the molecular constructs, data and phenotypic analysis. TP-A and LS-L steered the mycorrhizal phenotype analysis under overexpressing and silencing conditions. OS and JO contributed to the nodulation and mycorrhization assays. JO conducted the expression of the fusion proteins. AQ-H and AC-M conducted RT-qPCR analysis of mycorrhizal and nodulation kinetics. JP-M performed all the bioinformatics analysis. TP-A, SJ-J and LC conceived, designed the research and wrote the manuscript. All authors contributed to the article and approved the submitted version.
