# Supplementary material for: TETRASPANIN 8-1 from Phaseolus vulgaris plays a key role during mutualistic interactions
Source: Front Plant Sci. 2023 Jul 3;14:1152493. doi: 10.3389/fpls.2023.1152493 (PMC10352089; doi:10.3389/fpls.2023.1152493)
Supplement: Supplementary file 1 [file DataSheet_1.zip › Supplementary Information.DOCX]

***Supplementary Material***

**TETRASPANIN 8 from *Phaseolus vulgaris* plays a key role during mutualistic interactions**

**Thelma Jacqueline Parra-Aguilar^1^, Luis Gerardo Sarmiento-López^2^, Olivia Santana Estrada^1^, Juan Elías Olivares Grajales^1^, Edgar Pascual-Morales^1^, Saul Jiménez-Jiménez^1^,** **Andrea Quero-Hostos^1^, Janet Palacios-Martínez^1^, Ana I. Chávez-Martínez^1^ and Luis Cárdenas^1*^**

**^1^** Departamento de Biología Molecular de Plantas, Instituto de Biotecnología, Universidad Nacional Autónoma de México, Cuernavaca, Morelos, México.

^2^Departamento de Biotecnología Agrícola, Centro Interdisciplinario de Investigación para el Desarrollo Integral Regional Unidad Sinaloa-Instituto Politécnico Nacional, Guasave, Sinaloa, México.

**^*^Corresponding author**

Email: luis.cardenas@ibt.unam.mx

**Supplementary Figure 1.** Identifiers of the amino acid sequences of the tetraspanins used for the phylogenetic analysis, obtained from Phytozome date base (https://phytozome.jgi.doe.gov/pz/portal.html).

**Supplementary Figure 2.** Expression patterns of tetraspanins in *Phaseolus vulgaris*. (**A**) Heatmap representing expression profile of *P. vulgaris* tetraspanin genes in root formation and during nodulation. Data were obtained from Phytozome transcriptome repositories with RNA-Seq analysis of nodulation at different developmental stages of *P. vulgaris*. (**B**) Data of the transcriptional landscape of *PvTET* at different nodulation stages inoculated with *R. tropici* (Nod+, Fix+) and *R. giardini* (Nod+, Fix -). (**C**) Expression profile analysis of *PvTET8-1* during *Rhizobium* interaction. Relative expression of *PvENOD40a*, *PvLeghemoglobin* and *PvTET8-1* genes in wild-type *P. vulgaris* roots under nodulation conditions. Plants were inoculated with *R*. *tropici* and the roots were evaluated by RT-qPCR at 3, 5, 7, 14, 18, 21, 25 and 30 dpi. The elongation factor *PvEF1α* was used as endogenous reference gene to normalize expression levels. Bars represent mean ± standard error of the mean (SEM) of at least three independent biological replicates (*n*=3) with three technical repeats; white bars: uninoculated roots; black bars: inoculated roots with *R. tropici*. Asterisks indicate significant differences in relative expression compared with non-inoculated roots, *: *P<*0.05, **: *P<*0.01 and ***: *P<*0.001 (Student’s t-test).

**Supplementary Figure 3.** *PvTET8* promoter activity in transgenic *P*. *vulgaris* roots. **(A)** Analysis of cis-acting regulatory DNA elements in 1000 pb of the *PvTET8* promoter region. *OSE1ROOTNODULE* (AAAGAT), *OSE2ROOTNODULE* (CTCTT), *WRKY71OS* (TGAC), *GBOX* (CACGTG), *ARR1AT* (NGATT). **(B-D)** *PvTET8* promoter activity in *P. vulgaris* roots visualized by GUS staining in non-inoculated transgenic hairy roots expressing *pPvTET8::GFP-GUS*. **(B)** Root apical meristem, **(C)** vascular bundle, **(D)** lateral root meristem. Scale bars correspond to 50 µm.

**Supplementary Figure 4.** GUS histochemical analysis of transgenic hairy roots expressing the empty vector pBGWFS7 (control) inoculated with *R. tropici* and *R. irregularis.* (**A**) Histochemical analysis for nodule primordia at 7 dpi. (**B** and **C**) Nodules at 15 and 21 dpi, respectively. (**D**) Histochemical analysis for GUS in transgenic hairy roots carrying the empty vector at 14 dpi with *R. irregularis*. The GUS assay was counterstained with WGA-Alexa Fluor 488 to mark AM fungi. (**E**) WGA-Alexa Fluor 488-labeled mycorrhizal colonization of the root shown in **(D)**. **(F)** Merge of no GUS signal with arbuscle formation (green fluorescence). Scale bars correspond to 50 μm (**A-B**), 200 μm **(C)** and 20 μm (**D-F**).

**Supplementary Figure 5.** Subcellular localization of PvTET8-1-GFP in *P*. *vulgaris* inoculated with *R. tropici*. **(A)** Transgenic hairy root expressing *35S::GFP* as a control in early stages of infection thread formation at 5 dpi. **(B)** Rhizobia colonization on the surface of the responsive area in roots overexpressing PvTET8-1-GFP at 5 dpi. Note the increased bacterial colonization and root hair responses. **(C-D)** Roots overexpressing PvTET8-1-GFP during the infection thread formation. Note the increase bacterial colonization and cortical cell divisions. In green the root overexpressing *35S::PvTET8-1-GFP*. Red the Rhizobia expressing the DsRFP and merge of the two images. Last panel represent an inset of the merge image. Asterisks indicates the infection thread formation in root hairs. Scale bars correspond to 20 μm.

**Supplementary Figure 6.** Subcellular localization of PvTET8-1-GFP in *P*. *vulgaris* nodules. **(A)** Cross section of fresh transgenic nodules overexpressing *35S::GFP* as a control at 14 dpi depict the GFP in green, *R. tropici* expressing the DsRFP in red and colocalization in yellow. **(B)** Transversal section of a nodule overexpressing *35S::PvTET8-1-GFP*. In green the root overexpressing *35S::PvTET8-1-GFP*. Red the Rhizobia expressing the DsRFP and merge of the two images. Last panel represent an inset of the merge image. Note the increase nodule size in the overexpressing condition. Scale bars correspond to 20 μm.

**Supplementary Figure 7.** Changes in root dry weight, stem length, and leaf dry weight in *P. vulgaris* composite plants overexpressing the *PvTET8-1* gene during nodulation conditions at 21 dpi with *R. tropici*. **(A)** Root dry weight. **(B)** Stem length. **(C)** Leaf dry weight. *35S::GFP* construct was used as control and *35S::PvTET8-1-GFP* was used for overexpression. Bars represent mean ± SEM of 10 independent biological replicates (composite plants, *n*=10). Asterisks indicate significant differences, *: *P<*0.05, **: *P<*0.01 and ***: *P<*0.001 (Student’s t-test). **(D)** Representative composite plants carrying the *35S::GFP* or *35S::PvTET8-1-GFP* constructs. Scale bars correspond to 5 cm.

**Supplementary Figure 8.** Measurement of root dry weight, stem length, and leaf dry weight in *P. vulgaris* composite plants under silencing conditions at 21 dpi with *R. tropici*. **(A)** Root dry weight. **(B)** Stem length. **(C)** Leaf dry weight. *pTdT* vector was used as control and *PvTET8-1 RNAi* was used for silencing. Bars represent mean ± SEM of 10 independent biological replicates (composite plants, *n*=10). There were no significant differences *P<*0.05 (Student’s t-test). **(D)** Representative composite plants carrying the *pTdT* control or the *PvTET8-1 RNAi* construct. Scale bars correspond to 5 cm.

**Supplementary Figure 9.** Analysis of root dry weight, stem length, and leaf dry weight in *P. vulgaris* composite plants expressing the *35S::PvTET8-1-GFP* construct under mycorrhizal conditions. **(A)** Root dry weight. **(B)** Stem length. **(C)** Leaf dry weight. *35S::GFP* construct was used as control and *35S::PvTET8-1-GFP* was used for overexpression. Composite plants were colonized with *R. irregularis* (M+, filled bars) and non-colonized (M-, empty bars), and evaluated at 30 dpi. Bars represent mean ± SEM of 10 independent biological replicates (composite plants, *n*=10). Asterisks indicate significant differences, *: *P<*0.05, **: *P<*0.01 and ***: P<0.001, ANOVA post-hoc Tukey test. **(D)** Representative composite plants carrying the *35S::GFP* or *35S::PvTET8-1-GFP* constructs. Scale bars correspond to 5 cm.

**Supplementary Figure 10.** Measurement of root dry weight, stem length, and leaf dry weight in *P. vulgaris* composite plants downregulated in *PvTET8-1* expression and under mycorrhizal conditions. **(A)** Root dry weight. **(B)** Stem length. **(C)** Leaf dry weight. *pTdT* vector was used as control and *PvTET8-1 RNAi* was used for silencing. Composite plants were colonized with *R. irregularis* (M+, filled bars) and non-colonized (M-, empty bars), and evaluated at 30 dpi. Bars represent mean ± SEM of 10 independent biological replicates (composite plants, *n*=10). Asterisks indicate significant differences, *: *P*<0.05 and **: *P*<0.01, ANOVA post-hoc Tukey test. **(D)** Representative composite plants carrying the *pTdT* control or the *PvTET8-1 RNAi* construct. Scale bars correspond to 5 cm.

**Supplementary Figure 11.** Primers used for vectors construction and RT-qPCR assays.

**Supplementary Table 1**. Identifiers of the amino acid sequences of the tetraspanins used for the phylogenetic analysis, obtained from Phytozome v.13 database (https://phytozome.jgi.doe.gov/pz/portal.html). The percentages of identity and the associated e-values are shown. As well as the identifiers with their respective labels resulting from the phylogenetic analysis.

**Supplementary Video 1.** Localization of PvTET8-1-GFP at dynamic puncta in the cytoplasm in *N. benthamiana* pavement cells.

**Supplementary Video 2.** Localization of PvTET8-1-GFP at dynamic puncta in the cytoplasm in *P. vulgaris* root hair cells.

**Complementary figure 1** just for the revision. Root autofluorescence without expressing GFP.

**Complementary figure 2** just for the revision. Root depicting the *PvTET8* promoter activity, arbuscle localization and Transmitted light.
