# Supplementary material for: TETRASPANIN 8-1 from Phaseolus vulgaris plays a key role during mutualistic interactions
Source: Front Plant Sci. 2023 Jul 3;14:1152493. doi: 10.3389/fpls.2023.1152493 (PMC10352089; doi:10.3389/fpls.2023.1152493)
Supplement: Supplementary file 1 [file DataSheet_1.zip › Supplementary Table 1.PDF]

TET identity					
**Gene ID**	**Species**	**E-value**	**% identity**	**Align len**	**Query ID**
Lj2g0015394.1	L.japonicus Lj1.0v1	1.45E-119	70	269	AtTET1|AT5G46700
Glyma.09G205400.1.p	G.max Wm82.a4.v1	1.37E-116	70	269	AtTET1|AT5G46700
Medtr8g066790.1	M.truncatula Mt4.0v1	2.16E-114	70	269	AtTET1|AT5G46700
Lcu.2RBY.7g030120.1	L.culinaris v1	3.35E-112	67	269	AtTET1|AT5G46700
Glyma.01G095700.1.p	G.max Wm82.a4.v1	1.15E-111	68	269	AtTET1|AT5G46700
Ca_11504	C.arietinum v1.0	3.29E-111	67	269	AtTET1|AT5G46700
Medtr5g030070.1	M.truncatula Mt4.0v1	1.01E-110	68	269	AtTET1|AT5G46700
Phvul.002G140500.1.p	P.vulgaris v2.1	3.37E-110	68	269	AtTET1|AT5G46700
Lj4g0026647.1	L.japonicus Lj1.0v1	1.7E-109	67	269	AtTET1|AT5G46700
Lcu.2RBY.5g015480.1	L.culinaris v1	7.86E-109	67	269	AtTET1|AT5G46700
Phvul.003G284700.1.p	P.vulgaris v2.1	1.43E-106	64	269	AtTET1|AT5G46700
arahy.Tifrunner.gnm1.ann1.QC8A1V.1	A.hypogaea v1.0	2.32E-80	57	269	AtTET1|AT5G46700
arahy.Tifrunner.gnm1.ann1.EEBB9D.1	A.hypogaea v1.0	2.32E-80	57	269	AtTET1|AT5G46700
Ca_05887	C.arietinum v1.0	2.43E-75	49	269	AtTET1|AT5G46700
Lj4g0027264.1	L.japonicus Lj1.0v1	2.39E-73	50	269	AtTET1|AT5G46700
Phvul.002G304100.1.p	P.vulgaris v2.1	7.17E-73	49	269	AtTET1|AT5G46700
Glyma.08G029100.1.p	G.max Wm82.a4.v1	7.91E-72	50	269	AtTET1|AT5G46700
arahy.Tifrunner.gnm1.ann1.EM0Y56.1	A.hypogaea v1.0	8.25E-72	49	267	AtTET1|AT5G46700
arahy.Tifrunner.gnm1.ann1.N28W6S.1	A.hypogaea v1.0	1.44E-71	49	267	AtTET1|AT5G46700
Lcu.2RBY.4g078330.1	L.culinaris v1	2.42E-70	48	269	AtTET1|AT5G46700
Medtr8g101600.1	M.truncatula Mt4.0v1	4.04E-70	48	269	AtTET1|AT5G46700
Phvul.004G171300.1.p	P.vulgaris v2.1	3.46E-68	49	269	AtTET1|AT5G46700
Phvul.011G072100.1.p	P.vulgaris v2.1	7.49E-64	46	273	AtTET1|AT5G46700
arahy.Tifrunner.gnm1.ann1.B859HK.1	A.hypogaea v1.0	7.87E-64	46	265	AtTET1|AT5G46700
arahy.Tifrunner.gnm1.ann1.LZE6LN.1	A.hypogaea v1.0	8.68E-62	44	269	AtTET1|AT5G46700
arahy.Tifrunner.gnm1.ann1.PNW690.1	A.hypogaea v1.0	1.36E-61	47	231	AtTET1|AT5G46700
Glyma.07G129100.1.p	G.max Wm82.a4.v1	2.33E-61	47	273	AtTET1|AT5G46700
Glyma.13G091000.1.p	G.max Wm82.a4.v1	7.91E-61	46	261	AtTET1|AT5G46700
Phvul.003G151800.1.p	P.vulgaris v2.1	4.26E-60	46	260	AtTET1|AT5G46700
arahy.Tifrunner.gnm1.ann1.AUDB51.1	A.hypogaea v1.0	5.11E-60	45	273	AtTET1|AT5G46700
Glyma.12G072100.1.p	G.max Wm82.a4.v1	6.21E-60	46	273	AtTET1|AT5G46700
arahy.Tifrunner.gnm1.ann1.JZ8XUN.1	A.hypogaea v1.0	7.47E-60	45	273	AtTET1|AT5G46700
Glyma.11G152124.1.p	G.max Wm82.a4.v1	4.59E-59	46	273	AtTET1|AT5G46700
Lcu.2RBY.7g075810.1	L.culinaris v1	1.6E-58	45	265	AtTET1|AT5G46700
Lcu.2RBY.4g032690.1	L.culinaris v1	1.95E-58	47	235	AtTET1|AT5G46700

Lj2g0020248.1	L.japonicus Lj1.0v1	2.18E-58	48	226	AtTET1|AT5G46700
Ca_14606	C.arietinum v1.0	2.37E-58	45	273	AtTET1|AT5G46700
Medtr4g129670.1	M.truncatula Mt4.0v1	6E-58	46	267	AtTET1|AT5G46700
arahy.Tifrunner.gnm1.ann1.9I36N1.1	A.hypogaea v1.0	9.84E-58	46	260	AtTET1|AT5G46700
arahy.Tifrunner.gnm1.ann1.V2Z0GB.1	A.hypogaea v1.0	9.84E-58	46	260	AtTET1|AT5G46700
Lj4g0025241.1	L.japonicus Lj1.0v1	1.05E-57	45	262	AtTET1|AT5G46700
Medtr4g061010.1	M.truncatula Mt4.0v1	1.32E-57	47	235	AtTET1|AT5G46700
Glyma.17G069300.1.p	G.max Wm82.a4.v1	1.9E-57	45	262	AtTET1|AT5G46700
Lj3g0001285.1	L.japonicus Lj1.0v1	2.45E-56	45	274	AtTET1|AT5G46700
Glyma.13G328700.1.p	G.max Wm82.a4.v1	1.18E-55	42	238	AtTET1|AT5G46700
Lj6g0020177.1	L.japonicus Lj1.0v1	4.81E-54	46	260	AtTET1|AT5G46700
Medtr6g033335.1	M.truncatula Mt4.0v1	4.42E-53	43	257	AtTET1|AT5G46700
Phvul.005G137500.1.p	P.vulgaris v2.1	1.7E-52	44	237	AtTET1|AT5G46700
Lj2g0026181.1	L.japonicus Lj1.0v1	1.17E-51	43	257	AtTET1|AT5G46700
arahy.Tifrunner.gnm1.ann1.0ZGE19.1	A.hypogaea v1.0	1.9E-51	41	269	AtTET1|AT5G46700
Glyma.20G178700.1.p	G.max Wm82.a4.v1	2.35E-50	43	264	AtTET1|AT5G46700
arahy.Tifrunner.gnm1.ann1.K1NV0H.1	A.hypogaea v1.0	6.26E-50	61	127	AtTET1|AT5G46700
Lj5g0000955.1	L.japonicus Lj1.0v1	8E-50	43	264	AtTET1|AT5G46700
Glyma.10G212400.1.p	G.max Wm82.a4.v1	9.51E-50	43	264	AtTET1|AT5G46700
Lcu.2RBY.2g046220.1	L.culinaris v1	1.71E-49	43	254	AtTET1|AT5G46700
arahy.Tifrunner.gnm1.ann1.Z4U2NE.1	A.hypogaea v1.0	1.74E-49	42	266	AtTET1|AT5G46700
Phvul.004G071900.1.p	P.vulgaris v2.1	4.1E-49	44	254	AtTET1|AT5G46700
Phvul.007G093400.1.p	P.vulgaris v2.1	2.36E-48	42	264	AtTET1|AT5G46700
arahy.Tifrunner.gnm1.ann1.Z77BB1.1	A.hypogaea v1.0	3.38E-48	42	266	AtTET1|AT5G46700
Glyma.16G145400.1.p	G.max Wm82.a4.v1	6.84E-48	42	254	AtTET1|AT5G46700
Phvul.009G207500.1.p	P.vulgaris v2.1	5E-47	42	255	AtTET1|AT5G46700
Ca_08501	C.arietinum v1.0	7.82E-47	41	264	AtTET1|AT5G46700
Glyma.04G147100.1.p	G.max Wm82.a4.v1	6.34E-46	42	255	AtTET1|AT5G46700
Glyma.02G063700.1.p	G.max Wm82.a4.v1	2.31E-45	41	254	AtTET1|AT5G46700
Lcu.2RBY.1g046720.1	L.culinaris v1	2.78E-45	40	264	AtTET1|AT5G46700
Medtr1g095530.1	M.truncatula Mt4.0v1	5.33E-45	42	264	AtTET1|AT5G46700
Phvul.002G287500.1.p	P.vulgaris v2.1	7.15E-44	39	246	AtTET1|AT5G46700
Glyma.05G205600.1.p	G.max Wm82.a4.v1	8.49E-43	39	246	AtTET1|AT5G46700
arahy.Tifrunner.gnm1.ann1.8QTZ72.1	A.hypogaea v1.0	1.04E-42	37	255	AtTET1|AT5G46700
Lcu.2RBY.4g075230.1	L.culinaris v1	1.39E-41	38	256	AtTET1|AT5G46700
arahy.Tifrunner.gnm1.ann1.XJXR2L.1	A.hypogaea v1.0	2.18E-41	38	253	AtTET1|AT5G46700
Ca_03295	C.arietinum v1.0	6.7E-41	44	243	AtTET1|AT5G46700

Glyma.08G012500.2.p	G.max Wm82.a4.v1	8.92E-41	38	246	AtTET1|AT5G46700
arahy.Tifrunner.gnm1.ann1.2A0H1U.1	A.hypogaea v1.0	1.43E-40	38	253	AtTET1|AT5G46700
Medtr8g097280.1	M.truncatula Mt4.0v1	1.51E-40	38	218	AtTET1|AT5G46700
Phvul.003G180300.1.p	P.vulgaris v2.1	3.2E-40	35	260	AtTET1|AT5G46700
Glyma.05G016200.1.p	G.max Wm82.a4.v1	1.99E-39	34	261	AtTET1|AT5G46700
Glyma.17G084000.1.p	G.max Wm82.a4.v1	4.42E-39	33	261	AtTET1|AT5G46700
arahy.Tifrunner.gnm1.ann1.A2QH6A.1	A.hypogaea v1.0	3.31E-38	37	255	AtTET1|AT5G46700
Lj4g0013226.1	L.japonicus Lj1.0v1	1.87E-37	38	247	AtTET1|AT5G46700
arahy.Tifrunner.gnm1.ann1.T23S8A.1	A.hypogaea v1.0	2.9E-37	35	272	AtTET1|AT5G46700
Medtr4g112570.1	M.truncatula Mt4.0v1	5.73E-36	36	255	AtTET1|AT5G46700
arahy.Tifrunner.gnm1.ann1.4R894K.1	A.hypogaea v1.0	6.61E-36	39	254	AtTET1|AT5G46700
arahy.Tifrunner.gnm1.ann1.Z53AND.1	A.hypogaea v1.0	7.01E-36	34	272	AtTET1|AT5G46700
arahy.Tifrunner.gnm1.ann1.H32RIC.1	A.hypogaea v1.0	1.26E-35	40	166	AtTET1|AT5G46700
Lcu.2RBY.7g063730.1	L.culinaris v1	1.7E-34	34	260	AtTET1|AT5G46700
Ca_06587	C.arietinum v1.0	3.8E-34	36	255	AtTET1|AT5G46700
arahy.Tifrunner.gnm1.ann1.D9WPRA.1	A.hypogaea v1.0	4.52E-34	39	254	AtTET1|AT5G46700
Ca_05724	C.arietinum v1.0	1.24E-33	34	246	AtTET1|AT5G46700
Lj4g0018138.1	L.japonicus Lj1.0v1	1.59E-33	35	254	AtTET1|AT5G46700
Glyma.04G144500.1.p	G.max Wm82.a4.v1	8.71E-32	32	258	AtTET1|AT5G46700
Glyma.15G207300.1.p	G.max Wm82.a4.v1	1.46E-31	33	258	AtTET1|AT5G46700
arahy.Tifrunner.gnm1.ann1.8RU08H.1	A.hypogaea v1.0	3.59E-29	39	166	AtTET1|AT5G46700
Lj1g0003076.1	L.japonicus Lj1.0v1	3.63E-29	31	274	AtTET1|AT5G46700
Medtr2g056130.1	M.truncatula Mt4.0v1	9.53E-29	35	220	AtTET1|AT5G46700
Ca_23646	C.arietinum v1.0	1.9E-28	41	159	AtTET1|AT5G46700
Glyma.17G069300.2.p	G.max Wm82.a4.v1	1.42E-27	43	167	AtTET1|AT5G46700
Phvul.009G191200.1.p	P.vulgaris v2.1	2.21E-25	31	259	AtTET1|AT5G46700
Ca_25832	C.arietinum v1.0	9.19E-25	34	232	AtTET1|AT5G46700
Glyma.08G029100.1.p	G.max Wm82.a4.v1	1.14E-107	75	252	AtTET2|AT2G19580
Phvul.002G304100.1.p	P.vulgaris v2.1	7.59E-104	71	252	AtTET2|AT2G19580
arahy.Tifrunner.gnm1.ann1.N28W6S.1	A.hypogaea v1.0	1.03E-103	71	252	AtTET2|AT2G19580
arahy.Tifrunner.gnm1.ann1.EM0Y56.1	A.hypogaea v1.0	1.06E-103	71	252	AtTET2|AT2G19580
Ca_05887	C.arietinum v1.0	1.96E-103	71	252	AtTET2|AT2G19580
Lcu.2RBY.4g078330.1	L.culinaris v1	2.67E-103	71	252	AtTET2|AT2G19580
Medtr8g101600.1	M.truncatula Mt4.0v1	1.07E-102	71	252	AtTET2|AT2G19580
Glyma.07G129100.1.p	G.max Wm82.a4.v1	2.65E-102	68	255	AtTET2|AT2G19580
Lj4g0027264.1	L.japonicus Lj1.0v1	8.45E-100	69	252	AtTET2|AT2G19580
Phvul.004G171300.1.p	P.vulgaris v2.1	1.35E-99	67	252	AtTET2|AT2G19580

arahy.Tifrunner.gnm1.ann1.B859HK.1	A.hypogaea v1.0	4.1E-99	68	248	AtTET2|AT2G19580
arahy.Tifrunner.gnm1.ann1.PNW690.1	A.hypogaea v1.0	2.93E-93	71	214	AtTET2|AT2G19580
Lj2g0020248.1	L.japonicus Lj1.0v1	1.59E-90	70	209	AtTET2|AT2G19580
Lj2g0015394.1	L.japonicus Lj1.0v1	1.99E-79	56	251	AtTET2|AT2G19580
Glyma.01G095700.1.p	G.max Wm82.a4.v1	3.55E-79	55	251	AtTET2|AT2G19580
Lcu.2RBY.5g015480.1	L.culinaris v1	7.6E-79	58	251	AtTET2|AT2G19580
Ca_11504	C.arietinum v1.0	1.57E-77	57	251	AtTET2|AT2G19580
Glyma.09G205400.1.p	G.max Wm82.a4.v1	1.72E-76	58	251	AtTET2|AT2G19580
Medtr8g066790.1	M.truncatula Mt4.0v1	2.29E-74	53	251	AtTET2|AT2G19580
Medtr5g030070.1	M.truncatula Mt4.0v1	3.99E-74	59	251	AtTET2|AT2G19580
Phvul.003G284700.1.p	P.vulgaris v2.1	6.86E-74	53	251	AtTET2|AT2G19580
Lcu.2RBY.7g030120.1	L.culinaris v1	3.75E-72	53	249	AtTET2|AT2G19580
Lj4g0026647.1	L.japonicus Lj1.0v1	7.27E-72	53	251	AtTET2|AT2G19580
Phvul.002G140500.1.p	P.vulgaris v2.1	1.21E-70	55	251	AtTET2|AT2G19580
arahy.Tifrunner.gnm1.ann1.QC8A1V.1	A.hypogaea v1.0	6.51E-54	46	251	AtTET2|AT2G19580
arahy.Tifrunner.gnm1.ann1.EEBB9D.1	A.hypogaea v1.0	6.51E-54	46	251	AtTET2|AT2G19580
Ca_14606	C.arietinum v1.0	1.72E-53	46	247	AtTET2|AT2G19580
Phvul.011G072100.1.p	P.vulgaris v2.1	2.15E-53	45	247	AtTET2|AT2G19580
Medtr4g061010.1	M.truncatula Mt4.0v1	1.98E-52	46	248	AtTET2|AT2G19580
arahy.Tifrunner.gnm1.ann1.LZE6LN.1	A.hypogaea v1.0	1E-51	43	252	AtTET2|AT2G19580
Glyma.12G072100.1.p	G.max Wm82.a4.v1	1.86E-51	45	247	AtTET2|AT2G19580
Glyma.11G152124.1.p	G.max Wm82.a4.v1	1.22E-49	45	248	AtTET2|AT2G19580
Glyma.13G091000.1.p	G.max Wm82.a4.v1	1.27E-48	45	246	AtTET2|AT2G19580
Lj3g0001285.1	L.japonicus Lj1.0v1	1.91E-48	45	249	AtTET2|AT2G19580
Lj4g0025241.1	L.japonicus Lj1.0v1	4.91E-48	44	245	AtTET2|AT2G19580
Lcu.2RBY.7g075810.1	L.culinaris v1	6.51E-48	44	245	AtTET2|AT2G19580
Lcu.2RBY.4g032690.1	L.culinaris v1	2.3E-47	44	248	AtTET2|AT2G19580
arahy.Tifrunner.gnm1.ann1.JZ8XUN.1	A.hypogaea v1.0	8.06E-47	43	247	AtTET2|AT2G19580
Lj6g0020177.1	L.japonicus Lj1.0v1	1.54E-46	43	246	AtTET2|AT2G19580
arahy.Tifrunner.gnm1.ann1.9I36N1.1	A.hypogaea v1.0	1.58E-46	45	245	AtTET2|AT2G19580
arahy.Tifrunner.gnm1.ann1.V2Z0GB.1	A.hypogaea v1.0	1.58E-46	45	245	AtTET2|AT2G19580
arahy.Tifrunner.gnm1.ann1.AUDB51.1	A.hypogaea v1.0	1.96E-46	43	247	AtTET2|AT2G19580
Lj5g0000955.1	L.japonicus Lj1.0v1	6.88E-46	43	242	AtTET2|AT2G19580
Phvul.003G151800.1.p	P.vulgaris v2.1	1.12E-45	44	245	AtTET2|AT2G19580
arahy.Tifrunner.gnm1.ann1.K1NV0H.1	A.hypogaea v1.0	3.33E-45	60	128	AtTET2|AT2G19580
Glyma.16G145400.1.p	G.max Wm82.a4.v1	9.8E-45	45	239	AtTET2|AT2G19580
Glyma.17G069300.1.p	G.max Wm82.a4.v1	1.23E-44	43	246	AtTET2|AT2G19580

arahy.Tifrunner.gnm1.ann1.Z4U2NE.1	A.hypogaea v1.0	1.47E-44	41	242	AtTET2|AT2G19580
Medtr4g129670.1	M.truncatula Mt4.0v1	2.49E-44	44	250	AtTET2|AT2G19580
Glyma.13G328700.1.p	G.max Wm82.a4.v1	9.14E-44	39	244	AtTET2|AT2G19580
arahy.Tifrunner.gnm1.ann1.Z77BB1.1	A.hypogaea v1.0	1.47E-43	41	242	AtTET2|AT2G19580
Glyma.10G212400.1.p	G.max Wm82.a4.v1	6.09E-43	42	242	AtTET2|AT2G19580
Medtr1g095530.1	M.truncatula Mt4.0v1	6.09E-43	40	249	AtTET2|AT2G19580
Medtr6g033335.1	M.truncatula Mt4.0v1	3.67E-42	40	242	AtTET2|AT2G19580
Ca_08501	C.arietinum v1.0	6.32E-42	41	242	AtTET2|AT2G19580
Lj2g0026181.1	L.japonicus Lj1.0v1	7.21E-42	41	242	AtTET2|AT2G19580
Glyma.20G178700.1.p	G.max Wm82.a4.v1	9.9E-42	40	249	AtTET2|AT2G19580
Lcu.2RBY.1g046720.1	L.culinaris v1	2.07E-41	43	239	AtTET2|AT2G19580
Glyma.02G063700.1.p	G.max Wm82.a4.v1	3.07E-41	42	239	AtTET2|AT2G19580
Phvul.007G093400.1.p	P.vulgaris v2.1	3.07E-41	40	242	AtTET2|AT2G19580
Glyma.04G147100.1.p	G.max Wm82.a4.v1	4.7E-41	42	240	AtTET2|AT2G19580
Phvul.004G071900.1.p	P.vulgaris v2.1	6.34E-41	41	239	AtTET2|AT2G19580
arahy.Tifrunner.gnm1.ann1.0ZGE19.1	A.hypogaea v1.0	1.6E-40	38	252	AtTET2|AT2G19580
Lcu.2RBY.2g046220.1	L.culinaris v1	6.07E-40	41	239	AtTET2|AT2G19580
Phvul.009G207500.1.p	P.vulgaris v2.1	1.49E-38	42	240	AtTET2|AT2G19580
Phvul.005G137500.1.p	P.vulgaris v2.1	5.05E-38	38	244	AtTET2|AT2G19580
Ca_03295	C.arietinum v1.0	1.32E-37	44	238	AtTET2|AT2G19580
Medtr8g097280.1	M.truncatula Mt4.0v1	1.08E-36	37	212	AtTET2|AT2G19580
arahy.Tifrunner.gnm1.ann1.2A0H1U.1	A.hypogaea v1.0	2.33E-36	35	239	AtTET2|AT2G19580
Phvul.003G180300.1.p	P.vulgaris v2.1	7.2E-36	34	243	AtTET2|AT2G19580
Glyma.17G084000.1.p	G.max Wm82.a4.v1	7.21E-36	33	244	AtTET2|AT2G19580
Medtr4g112570.1	M.truncatula Mt4.0v1	7.69E-36	34	240	AtTET2|AT2G19580
Phvul.002G287500.1.p	P.vulgaris v2.1	7.71E-36	36	239	AtTET2|AT2G19580
arahy.Tifrunner.gnm1.ann1.XJXR2L.1	A.hypogaea v1.0	7.82E-36	35	239	AtTET2|AT2G19580
Lcu.2RBY.7g063730.1	L.culinaris v1	1.05E-35	32	239	AtTET2|AT2G19580
Lj4g0018138.1	L.japonicus Lj1.0v1	1.2E-35	33	246	AtTET2|AT2G19580
Glyma.05G205600.1.p	G.max Wm82.a4.v1	1.94E-35	35	239	AtTET2|AT2G19580
Lcu.2RBY.4g075230.1	L.culinaris v1	6.51E-35	34	239	AtTET2|AT2G19580
Glyma.08G012500.2.p	G.max Wm82.a4.v1	1.62E-34	35	239	AtTET2|AT2G19580
Glyma.05G016200.1.p	G.max Wm82.a4.v1	6.86E-34	32	244	AtTET2|AT2G19580
arahy.Tifrunner.gnm1.ann1.T23S8A.1	A.hypogaea v1.0	9.01E-34	35	234	AtTET2|AT2G19580
Ca_06587	C.arietinum v1.0	1.02E-33	33	234	AtTET2|AT2G19580
arahy.Tifrunner.gnm1.ann1.Z53AND.1	A.hypogaea v1.0	1.63E-33	34	235	AtTET2|AT2G19580
arahy.Tifrunner.gnm1.ann1.8QTZ72.1	A.hypogaea v1.0	7.38E-33	40	242	AtTET2|AT2G19580

Lj4g0013226.1	L.japonicus Lj1.0v1	1.24E-31	36	239	AtTET2|AT2G19580
arahy.Tifrunner.gnm1.ann1.A2QH6A.1	A.hypogaea v1.0	1.04E-30	36	239	AtTET2|AT2G19580
arahy.Tifrunner.gnm1.ann1.4R894K.1	A.hypogaea v1.0	4.12E-30	37	239	AtTET2|AT2G19580
Glyma.04G144500.1.p	G.max Wm82.a4.v1	4.4E-29	34	243	AtTET2|AT2G19580
Medtr2g056130.1	M.truncatula Mt4.0v1	8.8E-29	31	223	AtTET2|AT2G19580
Glyma.15G207300.1.p	G.max Wm82.a4.v1	9.26E-29	34	243	AtTET2|AT2G19580
Ca_23646	C.arietinum v1.0	1.1E-27	41	148	AtTET2|AT2G19580
arahy.Tifrunner.gnm1.ann1.H32RIC.1	A.hypogaea v1.0	4.77E-27	38	133	AtTET2|AT2G19580
Ca_25832	C.arietinum v1.0	5.41E-27	32	229	AtTET2|AT2G19580
arahy.Tifrunner.gnm1.ann1.D9WPRA.1	A.hypogaea v1.0	5.93E-26	36	239	AtTET2|AT2G19580
Lcu.2RBY.2g064540.1	L.culinaris v1	5.95E-26	33	224	AtTET2|AT2G19580
Lj1g0003076.1	L.japonicus Lj1.0v1	1.86E-25	30	258	AtTET2|AT2G19580
Ca_05724	C.arietinum v1.0	1.79E-24	35	162	AtTET2|AT2G19580
Phvul.009G191200.1.p	P.vulgaris v2.1	3.05E-22	32	244	AtTET2|AT2G19580
arahy.Tifrunner.gnm1.ann1.8RU08H.1	A.hypogaea v1.0	7.21E-20	34	133	AtTET2|AT2G19580
Medtr4g061010.1	M.truncatula Mt4.0v1	3.23E-143	78	285	AtTET3|AT3G45600
Phvul.011G072100.1.p	P.vulgaris v2.1	2E-141	76	285	AtTET3|AT3G45600
Ca_14606	C.arietinum v1.0	2.93E-140	78	285	AtTET3|AT3G45600
Lcu.2RBY.4g032690.1	L.culinaris v1	1.21E-138	75	285	AtTET3|AT3G45600
Lj3g0001285.1	L.japonicus Lj1.0v1	2.53E-138	78	286	AtTET3|AT3G45600
Glyma.12G072100.1.p	G.max Wm82.a4.v1	1.03E-137	76	285	AtTET3|AT3G45600
Glyma.11G152124.1.p	G.max Wm82.a4.v1	2.57E-137	76	285	AtTET3|AT3G45600
arahy.Tifrunner.gnm1.ann1.LZE6LN.1	A.hypogaea v1.0	3.99E-135	74	285	AtTET3|AT3G45600
arahy.Tifrunner.gnm1.ann1.AUDB51.1	A.hypogaea v1.0	2.22E-134	74	285	AtTET3|AT3G45600
arahy.Tifrunner.gnm1.ann1.JZ8XUN.1	A.hypogaea v1.0	3.07E-133	73	285	AtTET3|AT3G45600
Glyma.13G328700.1.p	G.max Wm82.a4.v1	2.86E-121	70	281	AtTET3|AT3G45600
arahy.Tifrunner.gnm1.ann1.0ZGE19.1	A.hypogaea v1.0	2.14E-118	69	285	AtTET3|AT3G45600
Phvul.005G137500.1.p	P.vulgaris v2.1	8.92E-108	62	281	AtTET3|AT3G45600
Lcu.2RBY.7g075810.1	L.culinaris v1	2.68E-72	49	234	AtTET3|AT3G45600
Medtr4g129670.1	M.truncatula Mt4.0v1	1.01E-71	49	235	AtTET3|AT3G45600
Glyma.17G069300.1.p	G.max Wm82.a4.v1	5.05E-70	47	236	AtTET3|AT3G45600
Phvul.003G151800.1.p	P.vulgaris v2.1	6.1E-70	49	235	AtTET3|AT3G45600
Glyma.13G091000.1.p	G.max Wm82.a4.v1	8.07E-70	48	236	AtTET3|AT3G45600
Glyma.01G095700.1.p	G.max Wm82.a4.v1	3.6E-69	47	235	AtTET3|AT3G45600
Lj4g0025241.1	L.japonicus Lj1.0v1	9.34E-69	46	235	AtTET3|AT3G45600
Lcu.2RBY.5g015480.1	L.culinaris v1	1.68E-67	46	235	AtTET3|AT3G45600
arahy.Tifrunner.gnm1.ann1.9I36N1.1	A.hypogaea v1.0	3.22E-67	47	234	AtTET3|AT3G45600

arahy.Tifrunner.gnm1.ann1.V2Z0GB.1	A.hypogaea v1.0	3.22E-67	47	234	AtTET3|AT3G45600
Lj5g0000955.1	L.japonicus Lj1.0v1	1E-66	45	279	AtTET3|AT3G45600
Lj2g0015394.1	L.japonicus Lj1.0v1	1.07E-66	46	235	AtTET3|AT3G45600
Lcu.2RBY.7g030120.1	L.culinaris v1	3.27E-66	45	235	AtTET3|AT3G45600
Phvul.007G093400.1.p	P.vulgaris v2.1	3.81E-66	46	279	AtTET3|AT3G45600
Ca_11504	C.arietinum v1.0	4.7E-65	45	235	AtTET3|AT3G45600
Phvul.003G284700.1.p	P.vulgaris v2.1	9.39E-65	44	235	AtTET3|AT3G45600
Lj4g0026647.1	L.japonicus Lj1.0v1	1.33E-64	45	235	AtTET3|AT3G45600
Glyma.09G205400.1.p	G.max Wm82.a4.v1	1.53E-64	45	235	AtTET3|AT3G45600
Glyma.20G178700.1.p	G.max Wm82.a4.v1	3.71E-64	44	279	AtTET3|AT3G45600
Glyma.10G212400.1.p	G.max Wm82.a4.v1	3.87E-64	45	279	AtTET3|AT3G45600
Medtr6g033335.1	M.truncatula Mt4.0v1	5.08E-64	44	279	AtTET3|AT3G45600
Lj2g0026181.1	L.japonicus Lj1.0v1	8.13E-64	44	279	AtTET3|AT3G45600
Phvul.009G207500.1.p	P.vulgaris v2.1	9.2E-64	47	235	AtTET3|AT3G45600
Lcu.2RBY.1g046720.1	L.culinaris v1	2.29E-63	44	279	AtTET3|AT3G45600
Glyma.04G147100.1.p	G.max Wm82.a4.v1	4.32E-63	48	234	AtTET3|AT3G45600
Phvul.002G140500.1.p	P.vulgaris v2.1	5.45E-63	43	235	AtTET3|AT3G45600
Ca_08501	C.arietinum v1.0	7.59E-63	44	279	AtTET3|AT3G45600
arahy.Tifrunner.gnm1.ann1.Z4U2NE.1	A.hypogaea v1.0	1.38E-62	44	279	AtTET3|AT3G45600
Glyma.16G145400.1.p	G.max Wm82.a4.v1	3.72E-62	43	279	AtTET3|AT3G45600
Lj6g0020177.1	L.japonicus Lj1.0v1	4.84E-62	48	235	AtTET3|AT3G45600
Glyma.02G063700.1.p	G.max Wm82.a4.v1	5.04E-62	45	279	AtTET3|AT3G45600
arahy.Tifrunner.gnm1.ann1.Z77BB1.1	A.hypogaea v1.0	5.11E-62	44	279	AtTET3|AT3G45600
Medtr1g095530.1	M.truncatula Mt4.0v1	7.36E-62	46	279	AtTET3|AT3G45600
Phvul.004G071900.1.p	P.vulgaris v2.1	5.2E-61	41	279	AtTET3|AT3G45600
Medtr8g066790.1	M.truncatula Mt4.0v1	3.33E-60	45	235	AtTET3|AT3G45600
Ca_05887	C.arietinum v1.0	3.84E-60	44	235	AtTET3|AT3G45600
Lcu.2RBY.2g046220.1	L.culinaris v1	4.31E-60	42	279	AtTET3|AT3G45600
arahy.Tifrunner.gnm1.ann1.N28W6S.1	A.hypogaea v1.0	5.12E-59	43	233	AtTET3|AT3G45600
arahy.Tifrunner.gnm1.ann1.EM0Y56.1	A.hypogaea v1.0	6.29E-59	43	233	AtTET3|AT3G45600
arahy.Tifrunner.gnm1.ann1.PNW690.1	A.hypogaea v1.0	2.95E-58	41	235	AtTET3|AT3G45600
Medtr8g101600.1	M.truncatula Mt4.0v1	4.13E-58	43	235	AtTET3|AT3G45600
Lcu.2RBY.4g078330.1	L.culinaris v1	7.33E-58	43	235	AtTET3|AT3G45600
arahy.Tifrunner.gnm1.ann1.B859HK.1	A.hypogaea v1.0	8.99E-57	40	235	AtTET3|AT3G45600
Medtr5g030070.1	M.truncatula Mt4.0v1	5.04E-56	46	235	AtTET3|AT3G45600
arahy.Tifrunner.gnm1.ann1.8QTZ72.1	A.hypogaea v1.0	1.18E-53	42	236	AtTET3|AT3G45600
arahy.Tifrunner.gnm1.ann1.4R894K.1	A.hypogaea v1.0	2.5E-53	42	276	AtTET3|AT3G45600

Phvul.002G304100.1.p	P.vulgaris v2.1	2.57E-53	43	235	AtTET3|AT3G45600
Lj4g0027264.1	L.japonicus Lj1.0v1	9.66E-53	43	235	AtTET3|AT3G45600
arahy.Tifrunner.gnm1.ann1.A2QH6A.1	A.hypogaea v1.0	2.61E-52	42	238	AtTET3|AT3G45600
Ca_03295	C.arietinum v1.0	1.86E-51	45	211	AtTET3|AT3G45600
Lj2g0020248.1	L.japonicus Lj1.0v1	2.66E-51	38	231	AtTET3|AT3G45600
arahy.Tifrunner.gnm1.ann1.D9WPRA.1	A.hypogaea v1.0	4.32E-51	41	279	AtTET3|AT3G45600
Glyma.08G029100.1.p	G.max Wm82.a4.v1	9.78E-51	43	235	AtTET3|AT3G45600
Lcu.2RBY.4g075230.1	L.culinaris v1	7.46E-50	35	234	AtTET3|AT3G45600
Medtr8g097280.1	M.truncatula Mt4.0v1	2.37E-49	34	234	AtTET3|AT3G45600
Phvul.004G171300.1.p	P.vulgaris v2.1	4.95E-49	39	236	AtTET3|AT3G45600
arahy.Tifrunner.gnm1.ann1.QC8A1V.1	A.hypogaea v1.0	3.78E-48	39	235	AtTET3|AT3G45600
arahy.Tifrunner.gnm1.ann1.EEBB9D.1	A.hypogaea v1.0	3.78E-48	39	235	AtTET3|AT3G45600
Glyma.07G129100.1.p	G.max Wm82.a4.v1	5.23E-47	39	240	AtTET3|AT3G45600
Phvul.002G287500.1.p	P.vulgaris v2.1	1.45E-46	34	234	AtTET3|AT3G45600
Glyma.08G012500.2.p	G.max Wm82.a4.v1	1.25E-45	33	234	AtTET3|AT3G45600
Glyma.05G205600.1.p	G.max Wm82.a4.v1	1.31E-45	33	234	AtTET3|AT3G45600
arahy.Tifrunner.gnm1.ann1.XJXR2L.1	A.hypogaea v1.0	1.79E-45	34	232	AtTET3|AT3G45600
arahy.Tifrunner.gnm1.ann1.2A0H1U.1	A.hypogaea v1.0	6.85E-45	34	232	AtTET3|AT3G45600
Lcu.2RBY.7g063730.1	L.culinaris v1	2.74E-44	36	235	AtTET3|AT3G45600
Glyma.17G084000.1.p	G.max Wm82.a4.v1	2.92E-44	36	237	AtTET3|AT3G45600
Lj4g0018138.1	L.japonicus Lj1.0v1	6.26E-44	37	235	AtTET3|AT3G45600
arahy.Tifrunner.gnm1.ann1.Z53AND.1	A.hypogaea v1.0	6.85E-44	38	202	AtTET3|AT3G45600
Glyma.17G069300.2.p	G.max Wm82.a4.v1	8.72E-44	48	171	AtTET3|AT3G45600
arahy.Tifrunner.gnm1.ann1.T23S8A.1	A.hypogaea v1.0	2.55E-43	37	203	AtTET3|AT3G45600
Medtr4g112570.1	M.truncatula Mt4.0v1	1.45E-41	34	235	AtTET3|AT3G45600
Lj4g0013226.1	L.japonicus Lj1.0v1	1.03E-40	34	232	AtTET3|AT3G45600
Phvul.003G180300.1.p	P.vulgaris v2.1	1.25E-39	35	236	AtTET3|AT3G45600
Glyma.05G016200.1.p	G.max Wm82.a4.v1	1.9E-39	35	237	AtTET3|AT3G45600
Ca_06587	C.arietinum v1.0	2.81E-39	34	236	AtTET3|AT3G45600
Lj1g0003076.1	L.japonicus Lj1.0v1	1.89E-36	32	238	AtTET3|AT3G45600
Glyma.04G144500.1.p	G.max Wm82.a4.v1	5.57E-35	34	236	AtTET3|AT3G45600
Glyma.15G207300.1.p	G.max Wm82.a4.v1	8.5E-35	34	236	AtTET3|AT3G45600
Medtr2g056130.1	M.truncatula Mt4.0v1	1.56E-34	35	235	AtTET3|AT3G45600
Lcu.2RBY.2g064540.1	L.culinaris v1	3.08E-34	35	237	AtTET3|AT3G45600
Ca_05724	C.arietinum v1.0	9.75E-33	29	234	AtTET3|AT3G45600
Phvul.009G191200.1.p	P.vulgaris v2.1	3.53E-31	31	236	AtTET3|AT3G45600
Ca_25832	C.arietinum v1.0	1.96E-28	34	236	AtTET3|AT3G45600

arahy.Tifrunner.gnm1.ann1.H32RIC.1	A.hypogaea v1.0	4.56E-28	31	241	AtTET3|AT3G45600
Ca_23646	C.arietinum v1.0	7.06E-27	39	171	AtTET3|AT3G45600
Phvul.011G072100.1.p	P.vulgaris v2.1	4.08E-131	73	277	AtTET4|AT5G60220
Glyma.11G152124.1.p	G.max Wm82.a4.v1	1.31E-129	73	277	AtTET4|AT5G60220
Glyma.12G072100.1.p	G.max Wm82.a4.v1	1.85E-129	73	277	AtTET4|AT5G60220
Medtr4g061010.1	M.truncatula Mt4.0v1	9.35E-129	73	276	AtTET4|AT5G60220
arahy.Tifrunner.gnm1.ann1.AUDB51.1	A.hypogaea v1.0	1.02E-127	71	277	AtTET4|AT5G60220
Ca_14606	C.arietinum v1.0	1.32E-126	72	276	AtTET4|AT5G60220
arahy.Tifrunner.gnm1.ann1.JZ8XUN.1	A.hypogaea v1.0	1.54E-126	71	277	AtTET4|AT5G60220
Lj3g0001285.1	L.japonicus Lj1.0v1	5.31E-126	73	278	AtTET4|AT5G60220
arahy.Tifrunner.gnm1.ann1.LZE6LN.1	A.hypogaea v1.0	1.45E-125	71	277	AtTET4|AT5G60220
Lcu.2RBY.4g032690.1	L.culinaris v1	9.57E-122	69	276	AtTET4|AT5G60220
Glyma.13G328700.1.p	G.max Wm82.a4.v1	3.86E-120	67	279	AtTET4|AT5G60220
arahy.Tifrunner.gnm1.ann1.0ZGE19.1	A.hypogaea v1.0	3.46E-109	65	277	AtTET4|AT5G60220
Phvul.005G137500.1.p	P.vulgaris v2.1	1.56E-104	59	278	AtTET4|AT5G60220
Lcu.2RBY.7g075810.1	L.culinaris v1	1.15E-71	47	262	AtTET4|AT5G60220
Glyma.13G091000.1.p	G.max Wm82.a4.v1	1.92E-71	48	264	AtTET4|AT5G60220
Glyma.17G069300.1.p	G.max Wm82.a4.v1	1.42E-70	47	264	AtTET4|AT5G60220
Phvul.003G151800.1.p	P.vulgaris v2.1	6.27E-70	47	263	AtTET4|AT5G60220
Medtr4g129670.1	M.truncatula Mt4.0v1	9.89E-70	47	263	AtTET4|AT5G60220
Lj5g0000955.1	L.japonicus Lj1.0v1	2.81E-68	44	277	AtTET4|AT5G60220
Phvul.007G093400.1.p	P.vulgaris v2.1	4.78E-68	44	277	AtTET4|AT5G60220
Lj4g0025241.1	L.japonicus Lj1.0v1	2.4E-67	45	259	AtTET4|AT5G60220
Glyma.10G212400.1.p	G.max Wm82.a4.v1	3.13E-67	44	277	AtTET4|AT5G60220
Glyma.20G178700.1.p	G.max Wm82.a4.v1	1.69E-66	43	277	AtTET4|AT5G60220
arahy.Tifrunner.gnm1.ann1.9I36N1.1	A.hypogaea v1.0	1.99E-66	46	264	AtTET4|AT5G60220
arahy.Tifrunner.gnm1.ann1.V2Z0GB.1	A.hypogaea v1.0	1.99E-66	46	264	AtTET4|AT5G60220
Glyma.16G145400.1.p	G.max Wm82.a4.v1	5.67E-66	45	278	AtTET4|AT5G60220
Lj2g0026181.1	L.japonicus Lj1.0v1	1.19E-64	45	277	AtTET4|AT5G60220
Glyma.02G063700.1.p	G.max Wm82.a4.v1	2.38E-64	44	278	AtTET4|AT5G60220
Medtr6g033335.1	M.truncatula Mt4.0v1	8.41E-64	44	277	AtTET4|AT5G60220
arahy.Tifrunner.gnm1.ann1.Z4U2NE.1	A.hypogaea v1.0	3.94E-63	42	278	AtTET4|AT5G60220
arahy.Tifrunner.gnm1.ann1.Z77BB1.1	A.hypogaea v1.0	4.94E-63	42	278	AtTET4|AT5G60220
Glyma.04G147100.1.p	G.max Wm82.a4.v1	1.04E-62	45	262	AtTET4|AT5G60220
Glyma.01G095700.1.p	G.max Wm82.a4.v1	2.42E-62	45	263	AtTET4|AT5G60220
Phvul.004G071900.1.p	P.vulgaris v2.1	2.57E-62	43	278	AtTET4|AT5G60220
Lj6g0020177.1	L.japonicus Lj1.0v1	4.75E-62	46	260	AtTET4|AT5G60220

Lj2g0015394.1	L.japonicus Lj1.0v1	5.09E-62	42	262	AtTET4|AT5G60220
Ca_08501	C.arietinum v1.0	7.32E-62	43	277	AtTET4|AT5G60220
Medtr8g066790.1	M.truncatula Mt4.0v1	6.22E-61	44	262	AtTET4|AT5G60220
Lcu.2RBY.7g030120.1	L.culinaris v1	9.27E-61	44	262	AtTET4|AT5G60220
Phvul.003G284700.1.p	P.vulgaris v2.1	1.7E-59	42	262	AtTET4|AT5G60220
Lcu.2RBY.2g046220.1	L.culinaris v1	2.4E-59	40	277	AtTET4|AT5G60220
Lj4g0026647.1	L.japonicus Lj1.0v1	2.72E-58	44	235	AtTET4|AT5G60220
Lcu.2RBY.1g046720.1	L.culinaris v1	3.48E-58	42	277	AtTET4|AT5G60220
Ca_05887	C.arietinum v1.0	4E-58	44	263	AtTET4|AT5G60220
Lj4g0027264.1	L.japonicus Lj1.0v1	1.55E-57	44	263	AtTET4|AT5G60220
Phvul.009G207500.1.p	P.vulgaris v2.1	4.75E-57	44	262	AtTET4|AT5G60220
Lcu.2RBY.5g015480.1	L.culinaris v1	4.81E-57	42	262	AtTET4|AT5G60220
Ca_11504	C.arietinum v1.0	9.48E-57	43	262	AtTET4|AT5G60220
Phvul.002G140500.1.p	P.vulgaris v2.1	2.06E-56	43	262	AtTET4|AT5G60220
arahy.Tifrunner.gnm1.ann1.B859HK.1	A.hypogaea v1.0	7.18E-56	38	265	AtTET4|AT5G60220
Glyma.09G205400.1.p	G.max Wm82.a4.v1	8.06E-56	44	262	AtTET4|AT5G60220
Lcu.2RBY.4g078330.1	L.culinaris v1	2.87E-55	45	262	AtTET4|AT5G60220
Medtr8g101600.1	M.truncatula Mt4.0v1	5.02E-55	43	263	AtTET4|AT5G60220
Medtr1g095530.1	M.truncatula Mt4.0v1	5.15E-55	44	277	AtTET4|AT5G60220
arahy.Tifrunner.gnm1.ann1.4R894K.1	A.hypogaea v1.0	1.85E-54	40	280	AtTET4|AT5G60220
Ca_03295	C.arietinum v1.0	1.99E-54	46	239	AtTET4|AT5G60220
Phvul.002G304100.1.p	P.vulgaris v2.1	2.33E-54	43	262	AtTET4|AT5G60220
arahy.Tifrunner.gnm1.ann1.8QTZ72.1	A.hypogaea v1.0	3.55E-54	40	242	AtTET4|AT5G60220
arahy.Tifrunner.gnm1.ann1.PNW690.1	A.hypogaea v1.0	3.7E-54	39	235	AtTET4|AT5G60220
arahy.Tifrunner.gnm1.ann1.N28W6S.1	A.hypogaea v1.0	5.04E-54	43	259	AtTET4|AT5G60220
arahy.Tifrunner.gnm1.ann1.EM0Y56.1	A.hypogaea v1.0	5.55E-54	43	259	AtTET4|AT5G60220
Medtr5g030070.1	M.truncatula Mt4.0v1	5.72E-53	44	262	AtTET4|AT5G60220
Glyma.08G029100.1.p	G.max Wm82.a4.v1	1.37E-51	44	263	AtTET4|AT5G60220
arahy.Tifrunner.gnm1.ann1.D9WPRA.1	A.hypogaea v1.0	1.41E-50	39	277	AtTET4|AT5G60220
Phvul.004G171300.1.p	P.vulgaris v2.1	1.25E-49	39	263	AtTET4|AT5G60220
arahy.Tifrunner.gnm1.ann1.A2QH6A.1	A.hypogaea v1.0	1.35E-48	40	235	AtTET4|AT5G60220
Lcu.2RBY.4g075230.1	L.culinaris v1	5.21E-47	35	224	AtTET4|AT5G60220
Phvul.002G287500.1.p	P.vulgaris v2.1	1.48E-46	36	257	AtTET4|AT5G60220
Glyma.07G129100.1.p	G.max Wm82.a4.v1	1.63E-46	39	262	AtTET4|AT5G60220
Medtr8g097280.1	M.truncatula Mt4.0v1	2.46E-46	35	224	AtTET4|AT5G60220
Lj2g0020248.1	L.japonicus Lj1.0v1	1.12E-45	40	233	AtTET4|AT5G60220
Glyma.08G012500.2.p	G.max Wm82.a4.v1	2.12E-45	35	257	AtTET4|AT5G60220

Glyma.05G205600.1.p	G.max Wm82.a4.v1	5.02E-45	35	257	AtTET4|AT5G60220
arahy.Tifrunner.gnm1.ann1.T23S8A.1	A.hypogaea v1.0	1.1E-44	38	237	AtTET4|AT5G60220
arahy.Tifrunner.gnm1.ann1.QC8A1V.1	A.hypogaea v1.0	1.78E-44	38	235	AtTET4|AT5G60220
arahy.Tifrunner.gnm1.ann1.EEBB9D.1	A.hypogaea v1.0	1.78E-44	38	235	AtTET4|AT5G60220
arahy.Tifrunner.gnm1.ann1.Z53AND.1	A.hypogaea v1.0	2.65E-44	38	237	AtTET4|AT5G60220
Glyma.17G084000.1.p	G.max Wm82.a4.v1	2.89E-44	36	236	AtTET4|AT5G60220
Glyma.05G016200.1.p	G.max Wm82.a4.v1	3.21E-44	36	236	AtTET4|AT5G60220
Phvul.003G180300.1.p	P.vulgaris v2.1	3.96E-44	35	235	AtTET4|AT5G60220
arahy.Tifrunner.gnm1.ann1.XJXR2L.1	A.hypogaea v1.0	2.96E-43	35	228	AtTET4|AT5G60220
Lcu.2RBY.7g063730.1	L.culinaris v1	3.2E-43	37	201	AtTET4|AT5G60220
arahy.Tifrunner.gnm1.ann1.2A0H1U.1	A.hypogaea v1.0	3.94E-43	35	228	AtTET4|AT5G60220
Medtr4g112570.1	M.truncatula Mt4.0v1	5.19E-42	37	201	AtTET4|AT5G60220
Glyma.17G069300.2.p	G.max Wm82.a4.v1	1.01E-41	46	171	AtTET4|AT5G60220
Lj4g0018138.1	L.japonicus Lj1.0v1	1.84E-41	37	201	AtTET4|AT5G60220
Ca_06587	C.arietinum v1.0	1.47E-39	34	235	AtTET4|AT5G60220
Lj4g0013226.1	L.japonicus Lj1.0v1	1.16E-38	34	257	AtTET4|AT5G60220
Medtr2g056130.1	M.truncatula Mt4.0v1	7.61E-38	36	231	AtTET4|AT5G60220
Glyma.04G144500.1.p	G.max Wm82.a4.v1	7.91E-36	34	267	AtTET4|AT5G60220
Glyma.15G207300.1.p	G.max Wm82.a4.v1	2.1E-35	34	267	AtTET4|AT5G60220
Lcu.2RBY.2g064540.1	L.culinaris v1	2.2E-35	33	240	AtTET4|AT5G60220
Ca_25832	C.arietinum v1.0	3.17E-32	35	203	AtTET4|AT5G60220
Lj1g0003076.1	L.japonicus Lj1.0v1	4.22E-32	33	238	AtTET4|AT5G60220
Phvul.009G191200.1.p	P.vulgaris v2.1	6.02E-32	31	235	AtTET4|AT5G60220
Ca_23646	C.arietinum v1.0	9.7E-29	40	169	AtTET4|AT5G60220
Ca_05724	C.arietinum v1.0	1.75E-27	29	253	AtTET4|AT5G60220
arahy.Tifrunner.gnm1.ann1.H32RIC.1	A.hypogaea v1.0	2.56E-26	30	265	AtTET4|AT5G60220
Lj5g0000955.1	L.japonicus Lj1.0v1	2.21E-119	59	279	AtTET5|AT4G23410
Glyma.10G212400.1.p	G.max Wm82.a4.v1	3.41E-117	59	279	AtTET5|AT4G23410
Ca_08501	C.arietinum v1.0	2.75E-116	59	281	AtTET5|AT4G23410
Phvul.007G093400.1.p	P.vulgaris v2.1	1.07E-115	58	281	AtTET5|AT4G23410
Glyma.20G178700.1.p	G.max Wm82.a4.v1	1.47E-114	58	279	AtTET5|AT4G23410
Glyma.16G145400.1.p	G.max Wm82.a4.v1	5.34E-114	58	281	AtTET5|AT4G23410
Phvul.004G071900.1.p	P.vulgaris v2.1	6.22E-114	58	281	AtTET5|AT4G23410
Glyma.02G063700.1.p	G.max Wm82.a4.v1	1.47E-113	58	281	AtTET5|AT4G23410
Lj2g0026181.1	L.japonicus Lj1.0v1	6.61E-111	59	280	AtTET5|AT4G23410
Lcu.2RBY.1g046720.1	L.culinaris v1	1.93E-110	59	281	AtTET5|AT4G23410
Medtr6g033335.1	M.truncatula Mt4.0v1	1.02E-109	56	281	AtTET5|AT4G23410

arahy.Tifrunner.gnm1.ann1.Z4U2NE.1	A.hypogaea v1.0	1.35E-106	57	275	AtTET5|AT4G23410
arahy.Tifrunner.gnm1.ann1.Z77BB1.1	A.hypogaea v1.0	2E-106	57	275	AtTET5|AT4G23410
Lcu.2RBY.2g046220.1	L.culinaris v1	4.17E-106	55	280	AtTET5|AT4G23410
Medtr1g095530.1	M.truncatula Mt4.0v1	1.94E-100	58	281	AtTET5|AT4G23410
arahy.Tifrunner.gnm1.ann1.D9WPRA.1	A.hypogaea v1.0	1.03E-93	54	268	AtTET5|AT4G23410
arahy.Tifrunner.gnm1.ann1.4R894K.1	A.hypogaea v1.0	2.18E-92	55	262	AtTET5|AT4G23410
Glyma.13G091000.1.p	G.max Wm82.a4.v1	4E-68	42	269	AtTET5|AT4G23410
Ca_23646	C.arietinum v1.0	1.92E-67	54	169	AtTET5|AT4G23410
Lcu.2RBY.7g075810.1	L.culinaris v1	1.92E-66	42	263	AtTET5|AT4G23410
Phvul.003G151800.1.p	P.vulgaris v2.1	2.56E-65	41	263	AtTET5|AT4G23410
Glyma.17G069300.1.p	G.max Wm82.a4.v1	6.56E-64	41	269	AtTET5|AT4G23410
arahy.Tifrunner.gnm1.ann1.LZE6LN.1	A.hypogaea v1.0	6.4E-63	41	276	AtTET5|AT4G23410
arahy.Tifrunner.gnm1.ann1.9I36N1.1	A.hypogaea v1.0	3.52E-62	43	261	AtTET5|AT4G23410
arahy.Tifrunner.gnm1.ann1.V2Z0GB.1	A.hypogaea v1.0	3.52E-62	43	261	AtTET5|AT4G23410
Lj4g0025241.1	L.japonicus Lj1.0v1	9.26E-61	42	264	AtTET5|AT4G23410
Glyma.04G147100.1.p	G.max Wm82.a4.v1	2.53E-60	42	259	AtTET5|AT4G23410
Lj6g0020177.1	L.japonicus Lj1.0v1	5.4E-60	42	263	AtTET5|AT4G23410
Phvul.011G072100.1.p	P.vulgaris v2.1	1.1E-58	39	280	AtTET5|AT4G23410
Medtr4g129670.1	M.truncatula Mt4.0v1	4.79E-57	42	259	AtTET5|AT4G23410
Phvul.009G207500.1.p	P.vulgaris v2.1	2E-54	42	264	AtTET5|AT4G23410
arahy.Tifrunner.gnm1.ann1.0ZGE19.1	A.hypogaea v1.0	5.27E-54	38	274	AtTET5|AT4G23410
Glyma.13G328700.1.p	G.max Wm82.a4.v1	1.16E-52	38	282	AtTET5|AT4G23410
Medtr4g061010.1	M.truncatula Mt4.0v1	1.25E-51	41	280	AtTET5|AT4G23410
Lj2g0015394.1	L.japonicus Lj1.0v1	1.56E-51	37	259	AtTET5|AT4G23410
Ca_14606	C.arietinum v1.0	2.18E-50	39	280	AtTET5|AT4G23410
Glyma.11G152124.1.p	G.max Wm82.a4.v1	4.99E-50	39	280	AtTET5|AT4G23410
arahy.Tifrunner.gnm1.ann1.JZ8XUN.1	A.hypogaea v1.0	5.99E-50	39	280	AtTET5|AT4G23410
arahy.Tifrunner.gnm1.ann1.AUDB51.1	A.hypogaea v1.0	7.03E-50	39	280	AtTET5|AT4G23410
arahy.Tifrunner.gnm1.ann1.N28W6S.1	A.hypogaea v1.0	1.01E-49	40	257	AtTET5|AT4G23410
arahy.Tifrunner.gnm1.ann1.EM0Y56.1	A.hypogaea v1.0	1.36E-49	40	257	AtTET5|AT4G23410
Medtr8g066790.1	M.truncatula Mt4.0v1	4.93E-49	37	259	AtTET5|AT4G23410
arahy.Tifrunner.gnm1.ann1.8QTZ72.1	A.hypogaea v1.0	5.07E-49	37	261	AtTET5|AT4G23410
Ca_03295	C.arietinum v1.0	1.57E-48	43	236	AtTET5|AT4G23410
Lcu.2RBY.4g032690.1	L.culinaris v1	2.05E-48	40	280	AtTET5|AT4G23410
Glyma.12G072100.1.p	G.max Wm82.a4.v1	2.23E-48	39	280	AtTET5|AT4G23410
Lj4g0027264.1	L.japonicus Lj1.0v1	2.26E-48	40	254	AtTET5|AT4G23410
Phvul.005G137500.1.p	P.vulgaris v2.1	2.85E-48	37	280	AtTET5|AT4G23410

Lj3g0001285.1	L.japonicus Lj1.0v1	6.21E-48	39	286	AtTET5|AT4G23410
Phvul.002G304100.1.p	P.vulgaris v2.1	7.15E-48	42	254	AtTET5|AT4G23410
Glyma.17G084000.1.p	G.max Wm82.a4.v1	2.08E-47	35	265	AtTET5|AT4G23410
Glyma.05G016200.1.p	G.max Wm82.a4.v1	2.13E-47	35	265	AtTET5|AT4G23410
Lcu.2RBY.7g030120.1	L.culinaris v1	3.26E-46	37	266	AtTET5|AT4G23410
arahy.Tifrunner.gnm1.ann1.A2QH6A.1	A.hypogaea v1.0	1.14E-45	38	258	AtTET5|AT4G23410
Phvul.002G287500.1.p	P.vulgaris v2.1	3.35E-45	34	254	AtTET5|AT4G23410
Glyma.05G205600.1.p	G.max Wm82.a4.v1	1.77E-44	33	256	AtTET5|AT4G23410
Phvul.003G284700.1.p	P.vulgaris v2.1	2.47E-44	36	260	AtTET5|AT4G23410
arahy.Tifrunner.gnm1.ann1.XJXR2L.1	A.hypogaea v1.0	3E-44	33	257	AtTET5|AT4G23410
Medtr8g101600.1	M.truncatula Mt4.0v1	2.9E-43	36	254	AtTET5|AT4G23410
arahy.Tifrunner.gnm1.ann1.B859HK.1	A.hypogaea v1.0	2.93E-43	36	256	AtTET5|AT4G23410
Glyma.01G095700.1.p	G.max Wm82.a4.v1	3.63E-43	36	259	AtTET5|AT4G23410
Glyma.08G029100.1.p	G.max Wm82.a4.v1	3.83E-43	38	254	AtTET5|AT4G23410
Ca_05887	C.arietinum v1.0	6.19E-43	39	254	AtTET5|AT4G23410
Phvul.003G180300.1.p	P.vulgaris v2.1	8.98E-43	35	260	AtTET5|AT4G23410
Glyma.09G205400.1.p	G.max Wm82.a4.v1	1.69E-42	39	254	AtTET5|AT4G23410
arahy.Tifrunner.gnm1.ann1.2A0H1U.1	A.hypogaea v1.0	2.39E-42	33	257	AtTET5|AT4G23410
Lcu.2RBY.4g075230.1	L.culinaris v1	9.74E-42	33	256	AtTET5|AT4G23410
Glyma.08G012500.2.p	G.max Wm82.a4.v1	1.33E-41	32	256	AtTET5|AT4G23410
Lcu.2RBY.4g078330.1	L.culinaris v1	3.03E-41	37	254	AtTET5|AT4G23410
arahy.Tifrunner.gnm1.ann1.T23S8A.1	A.hypogaea v1.0	3.28E-41	34	264	AtTET5|AT4G23410
Phvul.004G171300.1.p	P.vulgaris v2.1	1.28E-40	39	258	AtTET5|AT4G23410
Medtr5g030070.1	M.truncatula Mt4.0v1	2.53E-40	38	254	AtTET5|AT4G23410
arahy.Tifrunner.gnm1.ann1.Z53AND.1	A.hypogaea v1.0	5.46E-40	33	264	AtTET5|AT4G23410
Ca_11504	C.arietinum v1.0	1.36E-39	39	254	AtTET5|AT4G23410
Medtr2g056130.1	M.truncatula Mt4.0v1	3.05E-39	34	267	AtTET5|AT4G23410
arahy.Tifrunner.gnm1.ann1.PNW690.1	A.hypogaea v1.0	5.27E-39	36	227	AtTET5|AT4G23410
Lcu.2RBY.5g015480.1	L.culinaris v1	5.67E-39	37	254	AtTET5|AT4G23410
Lj4g0013226.1	L.japonicus Lj1.0v1	1.58E-38	32	259	AtTET5|AT4G23410
Lj4g0026647.1	L.japonicus Lj1.0v1	2.19E-38	36	259	AtTET5|AT4G23410
Medtr8g097280.1	M.truncatula Mt4.0v1	3.78E-38	33	249	AtTET5|AT4G23410
Glyma.07G129100.1.p	G.max Wm82.a4.v1	1.75E-36	38	258	AtTET5|AT4G23410
Lcu.2RBY.7g063730.1	L.culinaris v1	3.58E-36	33	266	AtTET5|AT4G23410
Phvul.002G140500.1.p	P.vulgaris v2.1	3.79E-36	36	253	AtTET5|AT4G23410
Lcu.2RBY.2g064540.1	L.culinaris v1	4.1E-36	33	261	AtTET5|AT4G23410
Ca_06587	C.arietinum v1.0	7.23E-36	32	258	AtTET5|AT4G23410

Medtr4g112570.1	M.truncatula Mt4.0v1	1.41E-35	32	266	AtTET5|AT4G23410
Lj4g0018138.1	L.japonicus Lj1.0v1	1.27E-34	32	267	AtTET5|AT4G23410
Ca_25832	C.arietinum v1.0	3.24E-34	33	274	AtTET5|AT4G23410
Phvul.009G191200.1.p	P.vulgaris v2.1	1.32E-33	33	260	AtTET5|AT4G23410
Glyma.04G144500.1.p	G.max Wm82.a4.v1	3.15E-33	33	258	AtTET5|AT4G23410
Glyma.15G207300.1.p	G.max Wm82.a4.v1	6.86E-33	33	258	AtTET5|AT4G23410
arahy.Tifrunner.gnm1.ann1.H32RIC.1	A.hypogaea v1.0	9.01E-33	34	177	AtTET5|AT4G23410
Lj1g0003076.1	L.japonicus Lj1.0v1	1.13E-32	34	279	AtTET5|AT4G23410
Ca_05724	C.arietinum v1.0	7.49E-32	26	259	AtTET5|AT4G23410
Lj2g0020248.1	L.japonicus Lj1.0v1	5.05E-31	37	224	AtTET5|AT4G23410
Glyma.17G069300.2.p	G.max Wm82.a4.v1	1.66E-29	43	169	AtTET5|AT4G23410
arahy.Tifrunner.gnm1.ann1.QC8A1V.1	A.hypogaea v1.0	1.84E-28	33	252	AtTET5|AT4G23410
arahy.Tifrunner.gnm1.ann1.EEBB9D.1	A.hypogaea v1.0	1.84E-28	33	252	AtTET5|AT4G23410
arahy.Tifrunner.gnm1.ann1.8RU08H.1	A.hypogaea v1.0	1.57E-27	31	175	AtTET5|AT4G23410
Glyma.10G212400.1.p	G.max Wm82.a4.v1	6.17E-135	71	284	AtTET6|AT3G12090
Glyma.20G178700.1.p	G.max Wm82.a4.v1	6.08E-134	70	284	AtTET6|AT3G12090
Lj5g0000955.1	L.japonicus Lj1.0v1	4.51E-133	70	284	AtTET6|AT3G12090
Phvul.007G093400.1.p	P.vulgaris v2.1	1.95E-132	70	284	AtTET6|AT3G12090
Lj2g0026181.1	L.japonicus Lj1.0v1	3.91E-127	68	282	AtTET6|AT3G12090
Ca_08501	C.arietinum v1.0	4.73E-127	68	284	AtTET6|AT3G12090
Phvul.004G071900.1.p	P.vulgaris v2.1	3.1E-126	67	284	AtTET6|AT3G12090
Glyma.16G145400.1.p	G.max Wm82.a4.v1	2.51E-125	67	284	AtTET6|AT3G12090
Medtr6g033335.1	M.truncatula Mt4.0v1	2E-124	64	284	AtTET6|AT3G12090
Lcu.2RBY.1g046720.1	L.culinaris v1	1.89E-123	68	284	AtTET6|AT3G12090
Glyma.02G063700.1.p	G.max Wm82.a4.v1	2.56E-123	66	284	AtTET6|AT3G12090
arahy.Tifrunner.gnm1.ann1.Z77BB1.1	A.hypogaea v1.0	4.21E-123	65	269	AtTET6|AT3G12090
arahy.Tifrunner.gnm1.ann1.Z4U2NE.1	A.hypogaea v1.0	1.47E-122	65	269	AtTET6|AT3G12090
Lcu.2RBY.2g046220.1	L.culinaris v1	4.93E-121	63	282	AtTET6|AT3G12090
Medtr1g095530.1	M.truncatula Mt4.0v1	1.07E-116	67	284	AtTET6|AT3G12090
arahy.Tifrunner.gnm1.ann1.4R894K.1	A.hypogaea v1.0	7.49E-111	63	263	AtTET6|AT3G12090
arahy.Tifrunner.gnm1.ann1.D9WPRA.1	A.hypogaea v1.0	1.17E-109	63	264	AtTET6|AT3G12090
Phvul.003G151800.1.p	P.vulgaris v2.1	2.44E-73	46	261	AtTET6|AT3G12090
Lj4g0025241.1	L.japonicus Lj1.0v1	1.64E-72	46	261	AtTET6|AT3G12090
Glyma.13G091000.1.p	G.max Wm82.a4.v1	1.2E-71	46	261	AtTET6|AT3G12090
arahy.Tifrunner.gnm1.ann1.9I36N1.1	A.hypogaea v1.0	3.03E-71	46	261	AtTET6|AT3G12090
arahy.Tifrunner.gnm1.ann1.V2Z0GB.1	A.hypogaea v1.0	3.03E-71	46	261	AtTET6|AT3G12090
arahy.Tifrunner.gnm1.ann1.LZE6LN.1	A.hypogaea v1.0	5.33E-71	45	276	AtTET6|AT3G12090

Lcu.2RBY.7g075810.1	L.culinaris v1	6.41E-70	45	261	AtTET6|AT3G12090
Glyma.17G069300.1.p	G.max Wm82.a4.v1	1.78E-68	45	262	AtTET6|AT3G12090
Phvul.011G072100.1.p	P.vulgaris v2.1	9.19E-68	44	280	AtTET6|AT3G12090
Medtr4g061010.1	M.truncatula Mt4.0v1	9.44E-67	45	280	AtTET6|AT3G12090
Medtr4g129670.1	M.truncatula Mt4.0v1	1.47E-66	44	259	AtTET6|AT3G12090
Ca_14606	C.arietinum v1.0	3.5E-66	44	280	AtTET6|AT3G12090
Lcu.2RBY.4g032690.1	L.culinaris v1	1.76E-65	44	280	AtTET6|AT3G12090
Lj6g0020177.1	L.japonicus Lj1.0v1	1.02E-64	45	261	AtTET6|AT3G12090
arahy.Tifrunner.gnm1.ann1.AUDB51.1	A.hypogaea v1.0	1.69E-64	44	280	AtTET6|AT3G12090
arahy.Tifrunner.gnm1.ann1.JZ8XUN.1	A.hypogaea v1.0	2.81E-64	44	280	AtTET6|AT3G12090
Glyma.11G152124.1.p	G.max Wm82.a4.v1	6.9E-64	43	280	AtTET6|AT3G12090
Glyma.12G072100.1.p	G.max Wm82.a4.v1	8.38E-64	43	280	AtTET6|AT3G12090
Lj3g0001285.1	L.japonicus Lj1.0v1	2.24E-63	43	281	AtTET6|AT3G12090
Glyma.04G147100.1.p	G.max Wm82.a4.v1	5.53E-63	42	264	AtTET6|AT3G12090
arahy.Tifrunner.gnm1.ann1.0ZGE19.1	A.hypogaea v1.0	1.76E-62	43	274	AtTET6|AT3G12090
Glyma.13G328700.1.p	G.max Wm82.a4.v1	5.74E-62	42	283	AtTET6|AT3G12090
Phvul.009G207500.1.p	P.vulgaris v2.1	1.47E-61	43	259	AtTET6|AT3G12090
Lj2g0015394.1	L.japonicus Lj1.0v1	8.25E-61	42	253	AtTET6|AT3G12090
Phvul.005G137500.1.p	P.vulgaris v2.1	4.61E-57	40	282	AtTET6|AT3G12090
arahy.Tifrunner.gnm1.ann1.8QTZ72.1	A.hypogaea v1.0	6.86E-57	39	261	AtTET6|AT3G12090
Glyma.01G095700.1.p	G.max Wm82.a4.v1	8.95E-56	42	254	AtTET6|AT3G12090
Glyma.09G205400.1.p	G.max Wm82.a4.v1	1.88E-55	42	253	AtTET6|AT3G12090
Ca_11504	C.arietinum v1.0	2.04E-54	42	253	AtTET6|AT3G12090
Phvul.003G284700.1.p	P.vulgaris v2.1	3.21E-54	41	254	AtTET6|AT3G12090
Ca_23646	C.arietinum v1.0	7.17E-54	58	173	AtTET6|AT3G12090
Lj4g0026647.1	L.japonicus Lj1.0v1	1.32E-53	42	254	AtTET6|AT3G12090
Glyma.17G084000.1.p	G.max Wm82.a4.v1	1.81E-53	37	268	AtTET6|AT3G12090
arahy.Tifrunner.gnm1.ann1.A2QH6A.1	A.hypogaea v1.0	2.08E-53	39	259	AtTET6|AT3G12090
Lcu.2RBY.7g030120.1	L.culinaris v1	2.63E-53	41	254	AtTET6|AT3G12090
Medtr8g066790.1	M.truncatula Mt4.0v1	6.73E-52	42	254	AtTET6|AT3G12090
Ca_03295	C.arietinum v1.0	8.98E-52	43	230	AtTET6|AT3G12090
Lcu.2RBY.5g015480.1	L.culinaris v1	2.58E-51	40	253	AtTET6|AT3G12090
Ca_05887	C.arietinum v1.0	6.11E-51	41	253	AtTET6|AT3G12090
arahy.Tifrunner.gnm1.ann1.N28W6S.1	A.hypogaea v1.0	6.36E-51	41	257	AtTET6|AT3G12090
arahy.Tifrunner.gnm1.ann1.EM0Y56.1	A.hypogaea v1.0	9.26E-51	41	257	AtTET6|AT3G12090
arahy.Tifrunner.gnm1.ann1.B859HK.1	A.hypogaea v1.0	1.47E-49	40	252	AtTET6|AT3G12090
Glyma.05G016200.1.p	G.max Wm82.a4.v1	3.26E-49	37	268	AtTET6|AT3G12090

Phvul.002G140500.1.p	P.vulgaris v2.1	1.49E-48	40	253	AtTET6|AT3G12090
arahy.Tifrunner.gnm1.ann1.PNW690.1	A.hypogaea v1.0	3.84E-48	39	226	AtTET6|AT3G12090
Medtr8g101600.1	M.truncatula Mt4.0v1	1.36E-47	40	253	AtTET6|AT3G12090
Ca_06587	C.arietinum v1.0	3.08E-47	37	259	AtTET6|AT3G12090
Lcu.2RBY.4g078330.1	L.culinaris v1	3.89E-47	41	253	AtTET6|AT3G12090
Lcu.2RBY.7g063730.1	L.culinaris v1	7.53E-47	36	267	AtTET6|AT3G12090
Medtr4g112570.1	M.truncatula Mt4.0v1	9.43E-47	37	267	AtTET6|AT3G12090
Glyma.17G069300.2.p	G.max Wm82.a4.v1	6.65E-45	44	169	AtTET6|AT3G12090
Phvul.002G287500.1.p	P.vulgaris v2.1	1.21E-44	32	250	AtTET6|AT3G12090
Phvul.003G180300.1.p	P.vulgaris v2.1	1.87E-44	36	267	AtTET6|AT3G12090
Lj4g0027264.1	L.japonicus Lj1.0v1	2.84E-44	42	253	AtTET6|AT3G12090
arahy.Tifrunner.gnm1.ann1.T23S8A.1	A.hypogaea v1.0	2.93E-44	35	265	AtTET6|AT3G12090
Phvul.002G304100.1.p	P.vulgaris v2.1	3.22E-44	40	253	AtTET6|AT3G12090
Medtr2g056130.1	M.truncatula Mt4.0v1	3.81E-44	36	270	AtTET6|AT3G12090
arahy.Tifrunner.gnm1.ann1.XJXR2L.1	A.hypogaea v1.0	4.4E-44	31	260	AtTET6|AT3G12090
Glyma.08G029100.1.p	G.max Wm82.a4.v1	1.59E-43	43	253	AtTET6|AT3G12090
arahy.Tifrunner.gnm1.ann1.Z53AND.1	A.hypogaea v1.0	7.72E-43	33	265	AtTET6|AT3G12090
Medtr5g030070.1	M.truncatula Mt4.0v1	1.47E-42	40	253	AtTET6|AT3G12090
Glyma.05G205600.1.p	G.max Wm82.a4.v1	1.7E-42	30	248	AtTET6|AT3G12090
Lj4g0018138.1	L.japonicus Lj1.0v1	1.72E-42	35	260	AtTET6|AT3G12090
arahy.Tifrunner.gnm1.ann1.2A0H1U.1	A.hypogaea v1.0	4.19E-42	31	260	AtTET6|AT3G12090
Lcu.2RBY.4g075230.1	L.culinaris v1	8.91E-42	31	241	AtTET6|AT3G12090
Glyma.04G144500.1.p	G.max Wm82.a4.v1	1.94E-41	34	274	AtTET6|AT3G12090
Lj2g0020248.1	L.japonicus Lj1.0v1	2.11E-41	40	223	AtTET6|AT3G12090
Glyma.08G012500.2.p	G.max Wm82.a4.v1	2.98E-41	31	244	AtTET6|AT3G12090
Glyma.15G207300.1.p	G.max Wm82.a4.v1	3.38E-41	34	274	AtTET6|AT3G12090
Medtr8g097280.1	M.truncatula Mt4.0v1	6.29E-41	32	218	AtTET6|AT3G12090
Phvul.009G191200.1.p	P.vulgaris v2.1	4.77E-40	34	257	AtTET6|AT3G12090
Phvul.004G171300.1.p	P.vulgaris v2.1	3.18E-39	40	255	AtTET6|AT3G12090
arahy.Tifrunner.gnm1.ann1.QC8A1V.1	A.hypogaea v1.0	3.21E-39	35	252	AtTET6|AT3G12090
arahy.Tifrunner.gnm1.ann1.EEBB9D.1	A.hypogaea v1.0	3.21E-39	35	252	AtTET6|AT3G12090
Lj4g0013226.1	L.japonicus Lj1.0v1	3.42E-38	31	248	AtTET6|AT3G12090
Ca_25832	C.arietinum v1.0	3.7E-38	36	267	AtTET6|AT3G12090
Lcu.2RBY.2g064540.1	L.culinaris v1	2.02E-36	35	260	AtTET6|AT3G12090
Glyma.07G129100.1.p	G.max Wm82.a4.v1	3.47E-36	39	258	AtTET6|AT3G12090
Lj1g0003076.1	L.japonicus Lj1.0v1	4.14E-35	32	279	AtTET6|AT3G12090
arahy.Tifrunner.gnm1.ann1.H32RIC.1	A.hypogaea v1.0	1.25E-25	33	174	AtTET6|AT3G12090

Ca_05724	C.arietinum v1.0	4.04E-25	25	244	AtTET6|AT3G12090
Glyma.13G091000.1.p	G.max Wm82.a4.v1	4.84E-113	66	265	AtTET7|AT4G28050
Phvul.003G151800.1.p	P.vulgaris v2.1	4.89E-110	64	264	AtTET7|AT4G28050
Glyma.17G069300.1.p	G.max Wm82.a4.v1	1.46E-107	64	266	AtTET7|AT4G28050
Lcu.2RBY.7g075810.1	L.culinaris v1	1.21E-106	64	265	AtTET7|AT4G28050
Lj4g0025241.1	L.japonicus Lj1.0v1	4.47E-106	65	261	AtTET7|AT4G28050
arahy.Tifrunner.gnm1.ann1.9I36N1.1	A.hypogaea v1.0	1.56E-105	62	264	AtTET7|AT4G28050
arahy.Tifrunner.gnm1.ann1.V2Z0GB.1	A.hypogaea v1.0	1.56E-105	62	264	AtTET7|AT4G28050
Medtr4g129670.1	M.truncatula Mt4.0v1	3.14E-105	63	263	AtTET7|AT4G28050
Lj6g0020177.1	L.japonicus Lj1.0v1	2.43E-104	65	264	AtTET7|AT4G28050
Phvul.009G207500.1.p	P.vulgaris v2.1	1.6E-101	62	261	AtTET7|AT4G28050
Glyma.04G147100.1.p	G.max Wm82.a4.v1	6.23E-99	62	260	AtTET7|AT4G28050
Ca_03295	C.arietinum v1.0	2.48E-84	62	239	AtTET7|AT4G28050
arahy.Tifrunner.gnm1.ann1.8QTZ72.1	A.hypogaea v1.0	4.23E-82	52	263	AtTET7|AT4G28050
arahy.Tifrunner.gnm1.ann1.A2QH6A.1	A.hypogaea v1.0	5.08E-73	51	262	AtTET7|AT4G28050
arahy.Tifrunner.gnm1.ann1.LZE6LN.1	A.hypogaea v1.0	1.94E-68	44	261	AtTET7|AT4G28050
Phvul.007G093400.1.p	P.vulgaris v2.1	2E-68	43	260	AtTET7|AT4G28050
Glyma.20G178700.1.p	G.max Wm82.a4.v1	2.01E-67	43	260	AtTET7|AT4G28050
Glyma.13G328700.1.p	G.max Wm82.a4.v1	2.35E-67	46	261	AtTET7|AT4G28050
Glyma.10G212400.1.p	G.max Wm82.a4.v1	5.83E-67	43	260	AtTET7|AT4G28050
Glyma.17G069300.2.p	G.max Wm82.a4.v1	1.16E-66	65	169	AtTET7|AT4G28050
Lj2g0015394.1	L.japonicus Lj1.0v1	1.8E-66	44	255	AtTET7|AT4G28050
Lj5g0000955.1	L.japonicus Lj1.0v1	2.19E-66	43	260	AtTET7|AT4G28050
arahy.Tifrunner.gnm1.ann1.Z4U2NE.1	A.hypogaea v1.0	2.46E-66	44	260	AtTET7|AT4G28050
Phvul.011G072100.1.p	P.vulgaris v2.1	8.35E-66	44	263	AtTET7|AT4G28050
Ca_14606	C.arietinum v1.0	2.12E-65	47	263	AtTET7|AT4G28050
arahy.Tifrunner.gnm1.ann1.Z77BB1.1	A.hypogaea v1.0	4.13E-65	44	260	AtTET7|AT4G28050
Phvul.003G284700.1.p	P.vulgaris v2.1	1.16E-64	44	255	AtTET7|AT4G28050
Phvul.005G137500.1.p	P.vulgaris v2.1	1.31E-64	44	260	AtTET7|AT4G28050
Medtr4g061010.1	M.truncatula Mt4.0v1	1.89E-64	46	263	AtTET7|AT4G28050
Medtr6g033335.1	M.truncatula Mt4.0v1	1.99E-64	44	260	AtTET7|AT4G28050
Glyma.12G072100.1.p	G.max Wm82.a4.v1	2.49E-63	47	263	AtTET7|AT4G28050
Lj4g0026647.1	L.japonicus Lj1.0v1	6.33E-63	43	255	AtTET7|AT4G28050
Lcu.2RBY.1g046720.1	L.culinaris v1	6.37E-63	41	264	AtTET7|AT4G28050
Phvul.004G071900.1.p	P.vulgaris v2.1	7.17E-63	43	260	AtTET7|AT4G28050
Lj2g0026181.1	L.japonicus Lj1.0v1	8.4E-63	43	260	AtTET7|AT4G28050
Glyma.09G205400.1.p	G.max Wm82.a4.v1	9.36E-63	44	255	AtTET7|AT4G28050

Glyma.01G095700.1.p	G.max Wm82.a4.v1	9.66E-63	43	255	AtTET7|AT4G28050
arahy.Tifrunner.gnm1.ann1.JZ8XUN.1	A.hypogaea v1.0	1.66E-62	46	263	AtTET7|AT4G28050
Ca_05887	C.arietinum v1.0	1.75E-62	45	257	AtTET7|AT4G28050
Lj3g0001285.1	L.japonicus Lj1.0v1	2.66E-62	46	265	AtTET7|AT4G28050
Phvul.002G140500.1.p	P.vulgaris v2.1	4.17E-62	43	255	AtTET7|AT4G28050
arahy.Tifrunner.gnm1.ann1.AUDB51.1	A.hypogaea v1.0	7.08E-62	45	263	AtTET7|AT4G28050
Lcu.2RBY.7g030120.1	L.culinaris v1	1.01E-61	44	255	AtTET7|AT4G28050
Ca_08501	C.arietinum v1.0	1.25E-61	41	260	AtTET7|AT4G28050
Glyma.16G145400.1.p	G.max Wm82.a4.v1	2.82E-61	42	260	AtTET7|AT4G28050
Glyma.11G152124.1.p	G.max Wm82.a4.v1	7.25E-61	46	260	AtTET7|AT4G28050
Lcu.2RBY.4g032690.1	L.culinaris v1	8.26E-61	45	263	AtTET7|AT4G28050
arahy.Tifrunner.gnm1.ann1.0ZGE19.1	A.hypogaea v1.0	1.44E-60	42	261	AtTET7|AT4G28050
Ca_11504	C.arietinum v1.0	6.85E-60	43	255	AtTET7|AT4G28050
Glyma.17G084000.1.p	G.max Wm82.a4.v1	7.94E-60	39	265	AtTET7|AT4G28050
Lcu.2RBY.5g015480.1	L.culinaris v1	1.24E-59	41	255	AtTET7|AT4G28050
Glyma.02G063700.1.p	G.max Wm82.a4.v1	2.83E-59	41	260	AtTET7|AT4G28050
Medtr8g101600.1	M.truncatula Mt4.0v1	3.63E-59	42	257	AtTET7|AT4G28050
Medtr1g095530.1	M.truncatula Mt4.0v1	7.98E-59	41	260	AtTET7|AT4G28050
Lcu.2RBY.2g046220.1	L.culinaris v1	1.3E-58	41	260	AtTET7|AT4G28050
Medtr8g066790.1	M.truncatula Mt4.0v1	2.55E-58	45	255	AtTET7|AT4G28050
arahy.Tifrunner.gnm1.ann1.B859HK.1	A.hypogaea v1.0	4.1E-58	41	256	AtTET7|AT4G28050
Lcu.2RBY.4g078330.1	L.culinaris v1	2.01E-56	44	257	AtTET7|AT4G28050
arahy.Tifrunner.gnm1.ann1.PNW690.1	A.hypogaea v1.0	3E-56	42	228	AtTET7|AT4G28050
arahy.Tifrunner.gnm1.ann1.Z53AND.1	A.hypogaea v1.0	4E-55	40	264	AtTET7|AT4G28050
Glyma.05G016200.1.p	G.max Wm82.a4.v1	2.66E-54	39	265	AtTET7|AT4G28050
arahy.Tifrunner.gnm1.ann1.T23S8A.1	A.hypogaea v1.0	6.91E-54	39	264	AtTET7|AT4G28050
Lj4g0027264.1	L.japonicus Lj1.0v1	1.04E-52	43	257	AtTET7|AT4G28050
arahy.Tifrunner.gnm1.ann1.N28W6S.1	A.hypogaea v1.0	1.11E-52	41	261	AtTET7|AT4G28050
arahy.Tifrunner.gnm1.ann1.EM0Y56.1	A.hypogaea v1.0	1.34E-52	41	261	AtTET7|AT4G28050
arahy.Tifrunner.gnm1.ann1.XJXR2L.1	A.hypogaea v1.0	1.87E-52	37	260	AtTET7|AT4G28050
Lj4g0018138.1	L.japonicus Lj1.0v1	2.86E-52	38	262	AtTET7|AT4G28050
arahy.Tifrunner.gnm1.ann1.2A0H1U.1	A.hypogaea v1.0	3.32E-52	37	260	AtTET7|AT4G28050
arahy.Tifrunner.gnm1.ann1.4R894K.1	A.hypogaea v1.0	4.05E-52	37	260	AtTET7|AT4G28050
Phvul.003G180300.1.p	P.vulgaris v2.1	8.85E-52	39	264	AtTET7|AT4G28050
arahy.Tifrunner.gnm1.ann1.D9WPRA.1	A.hypogaea v1.0	1.57E-51	37	260	AtTET7|AT4G28050
Medtr5g030070.1	M.truncatula Mt4.0v1	5.75E-51	44	255	AtTET7|AT4G28050
Medtr4g112570.1	M.truncatula Mt4.0v1	1.49E-50	39	262	AtTET7|AT4G28050

Ca_06587	C.arietinum v1.0	2.06E-50	38	262	AtTET7|AT4G28050
Phvul.002G287500.1.p	P.vulgaris v2.1	1.85E-49	36	251	AtTET7|AT4G28050
Lcu.2RBY.7g063730.1	L.culinaris v1	1.16E-48	38	262	AtTET7|AT4G28050
Glyma.08G029100.1.p	G.max Wm82.a4.v1	4.32E-48	43	258	AtTET7|AT4G28050
Glyma.05G205600.1.p	G.max Wm82.a4.v1	8.25E-48	36	247	AtTET7|AT4G28050
Lj4g0013226.1	L.japonicus Lj1.0v1	1.07E-47	36	250	AtTET7|AT4G28050
Phvul.002G304100.1.p	P.vulgaris v2.1	3.87E-47	40	257	AtTET7|AT4G28050
Lcu.2RBY.4g075230.1	L.culinaris v1	4.97E-47	36	247	AtTET7|AT4G28050
arahy.Tifrunner.gnm1.ann1.QC8A1V.1	A.hypogaea v1.0	5.68E-47	38	253	AtTET7|AT4G28050
arahy.Tifrunner.gnm1.ann1.EEBB9D.1	A.hypogaea v1.0	5.68E-47	38	253	AtTET7|AT4G28050
Glyma.08G012500.2.p	G.max Wm82.a4.v1	1.33E-45	36	247	AtTET7|AT4G28050
Lj2g0020248.1	L.japonicus Lj1.0v1	1.04E-44	39	223	AtTET7|AT4G28050
Medtr2g056130.1	M.truncatula Mt4.0v1	3.41E-44	41	227	AtTET7|AT4G28050
Phvul.004G171300.1.p	P.vulgaris v2.1	8.09E-43	38	258	AtTET7|AT4G28050
Ca_25832	C.arietinum v1.0	1.08E-42	40	230	AtTET7|AT4G28050
Lj1g0003076.1	L.japonicus Lj1.0v1	3.47E-42	38	270	AtTET7|AT4G28050
Medtr8g097280.1	M.truncatula Mt4.0v1	3.75E-42	35	227	AtTET7|AT4G28050
Glyma.07G129100.1.p	G.max Wm82.a4.v1	2.93E-41	39	269	AtTET7|AT4G28050
Glyma.04G144500.1.p	G.max Wm82.a4.v1	4.36E-41	37	263	AtTET7|AT4G28050
Glyma.15G207300.1.p	G.max Wm82.a4.v1	8.93E-41	37	263	AtTET7|AT4G28050
Lcu.2RBY.2g064540.1	L.culinaris v1	4.48E-37	37	266	AtTET7|AT4G28050
arahy.Tifrunner.gnm1.ann1.H32RIC.1	A.hypogaea v1.0	1.15E-36	41	165	AtTET7|AT4G28050
Phvul.009G191200.1.p	P.vulgaris v2.1	9.06E-36	33	262	AtTET7|AT4G28050
Ca_05724	C.arietinum v1.0	1.93E-35	31	254	AtTET7|AT4G28050
arahy.Tifrunner.gnm1.ann1.8RU08H.1	A.hypogaea v1.0	1.62E-30	31	243	AtTET7|AT4G28050
Phvul.003G151800.1.p	P.vulgaris v2.1	3.3E-114	69	263	AtTET8|AT2G23810
Glyma.13G091000.1.p	G.max Wm82.a4.v1	5.36E-111	66	270	AtTET8|AT2G23810
Lcu.2RBY.7g075810.1	L.culinaris v1	1.74E-110	67	263	AtTET8|AT2G23810
Glyma.17G069300.1.p	G.max Wm82.a4.v1	1.09E-108	64	270	AtTET8|AT2G23810
arahy.Tifrunner.gnm1.ann1.9I36N1.1	A.hypogaea v1.0	2.28E-108	66	263	AtTET8|AT2G23810
arahy.Tifrunner.gnm1.ann1.V2Z0GB.1	A.hypogaea v1.0	2.28E-108	66	263	AtTET8|AT2G23810
Lj4g0025241.1	L.japonicus Lj1.0v1	3.56E-107	67	270	AtTET8|AT2G23810
Medtr4g129670.1	M.truncatula Mt4.0v1	1.13E-104	66	260	AtTET8|AT2G23810
Lj6g0020177.1	L.japonicus Lj1.0v1	1.62E-100	65	263	AtTET8|AT2G23810
Phvul.009G207500.1.p	P.vulgaris v2.1	3.91E-96	62	263	AtTET8|AT2G23810
Glyma.04G147100.1.p	G.max Wm82.a4.v1	4.5E-91	62	261	AtTET8|AT2G23810
Ca_03295	C.arietinum v1.0	1.13E-84	66	238	AtTET8|AT2G23810

arahy.Tifrunner.gnm1.ann1.8QTZ72.1	A.hypogaea v1.0	1.43E-76	53	259	AtTET8|AT2G23810
Glyma.17G069300.2.p	G.max Wm82.a4.v1	9.41E-75	67	169	AtTET8|AT2G23810
Phvul.011G072100.1.p	P.vulgaris v2.1	3.23E-72	49	265	AtTET8|AT2G23810
arahy.Tifrunner.gnm1.ann1.LZE6LN.1	A.hypogaea v1.0	5.78E-72	48	263	AtTET8|AT2G23810
Ca_14606	C.arietinum v1.0	3.25E-70	49	265	AtTET8|AT2G23810
arahy.Tifrunner.gnm1.ann1.A2QH6A.1	A.hypogaea v1.0	4.1E-70	53	258	AtTET8|AT2G23810
Medtr4g061010.1	M.truncatula Mt4.0v1	2.88E-69	49	265	AtTET8|AT2G23810
Lcu.2RBY.4g032690.1	L.culinaris v1	1.13E-68	48	265	AtTET8|AT2G23810
Lj3g0001285.1	L.japonicus Lj1.0v1	1.19E-67	48	266	AtTET8|AT2G23810
arahy.Tifrunner.gnm1.ann1.JZ8XUN.1	A.hypogaea v1.0	1.37E-67	48	265	AtTET8|AT2G23810
arahy.Tifrunner.gnm1.ann1.AUDB51.1	A.hypogaea v1.0	2.11E-67	48	265	AtTET8|AT2G23810
Glyma.11G152124.1.p	G.max Wm82.a4.v1	9.94E-67	48	265	AtTET8|AT2G23810
Glyma.12G072100.1.p	G.max Wm82.a4.v1	1.75E-66	48	265	AtTET8|AT2G23810
Glyma.13G328700.1.p	G.max Wm82.a4.v1	1.42E-65	46	263	AtTET8|AT2G23810
Glyma.10G212400.1.p	G.max Wm82.a4.v1	2.1E-65	42	264	AtTET8|AT2G23810
Glyma.20G178700.1.p	G.max Wm82.a4.v1	2.5E-65	42	264	AtTET8|AT2G23810
Lj5g0000955.1	L.japonicus Lj1.0v1	3.65E-65	42	264	AtTET8|AT2G23810
arahy.Tifrunner.gnm1.ann1.Z4U2NE.1	A.hypogaea v1.0	7.14E-65	44	264	AtTET8|AT2G23810
Lj2g0015394.1	L.japonicus Lj1.0v1	8.69E-65	45	263	AtTET8|AT2G23810
arahy.Tifrunner.gnm1.ann1.Z77BB1.1	A.hypogaea v1.0	1.74E-64	44	264	AtTET8|AT2G23810
Phvul.007G093400.1.p	P.vulgaris v2.1	1.83E-64	41	264	AtTET8|AT2G23810
Medtr6g033335.1	M.truncatula Mt4.0v1	3.47E-64	44	264	AtTET8|AT2G23810
Phvul.004G071900.1.p	P.vulgaris v2.1	1.67E-63	42	264	AtTET8|AT2G23810
Lj2g0026181.1	L.japonicus Lj1.0v1	4.54E-63	42	264	AtTET8|AT2G23810
Phvul.005G137500.1.p	P.vulgaris v2.1	9.48E-63	46	265	AtTET8|AT2G23810
Ca_08501	C.arietinum v1.0	1.3E-62	40	264	AtTET8|AT2G23810
Lcu.2RBY.1g046720.1	L.culinaris v1	2.78E-62	41	264	AtTET8|AT2G23810
Glyma.02G063700.1.p	G.max Wm82.a4.v1	4.83E-62	41	264	AtTET8|AT2G23810
Glyma.16G145400.1.p	G.max Wm82.a4.v1	1.97E-61	41	264	AtTET8|AT2G23810
Phvul.003G284700.1.p	P.vulgaris v2.1	3E-61	44	263	AtTET8|AT2G23810
arahy.Tifrunner.gnm1.ann1.0ZGE19.1	A.hypogaea v1.0	5.73E-61	44	263	AtTET8|AT2G23810
Glyma.09G205400.1.p	G.max Wm82.a4.v1	4.34E-60	42	263	AtTET8|AT2G23810
Glyma.01G095700.1.p	G.max Wm82.a4.v1	5.33E-60	43	263	AtTET8|AT2G23810
Glyma.17G084000.1.p	G.max Wm82.a4.v1	1.52E-58	39	267	AtTET8|AT2G23810
Phvul.002G140500.1.p	P.vulgaris v2.1	1.1E-57	42	263	AtTET8|AT2G23810
Lcu.2RBY.7g030120.1	L.culinaris v1	1.76E-57	41	263	AtTET8|AT2G23810
Ca_05887	C.arietinum v1.0	2.48E-57	43	263	AtTET8|AT2G23810

Lcu.2RBY.2g046220.1	L.culinaris v1	3.84E-57	39	264	AtTET8|AT2G23810
Lj4g0026647.1	L.japonicus Lj1.0v1	1.2E-56	41	263	AtTET8|AT2G23810
Lcu.2RBY.5g015480.1	L.culinaris v1	1.23E-56	43	263	AtTET8|AT2G23810
Medtr1g095530.1	M.truncatula Mt4.0v1	1.67E-56	39	264	AtTET8|AT2G23810
Medtr8g101600.1	M.truncatula Mt4.0v1	5.16E-56	42	263	AtTET8|AT2G23810
arahy.Tifrunner.gnm1.ann1.N28W6S.1	A.hypogaea v1.0	7.35E-56	43	267	AtTET8|AT2G23810
arahy.Tifrunner.gnm1.ann1.EM0Y56.1	A.hypogaea v1.0	1.01E-55	43	267	AtTET8|AT2G23810
Ca_11504	C.arietinum v1.0	8.19E-55	42	263	AtTET8|AT2G23810
arahy.Tifrunner.gnm1.ann1.4R894K.1	A.hypogaea v1.0	2.32E-54	37	272	AtTET8|AT2G23810
Lcu.2RBY.4g078330.1	L.culinaris v1	8.79E-54	43	263	AtTET8|AT2G23810
arahy.Tifrunner.gnm1.ann1.B859HK.1	A.hypogaea v1.0	1.71E-53	40	260	AtTET8|AT2G23810
Glyma.05G016200.1.p	G.max Wm82.a4.v1	8.57E-53	39	264	AtTET8|AT2G23810
arahy.Tifrunner.gnm1.ann1.D9WPRA.1	A.hypogaea v1.0	9.42E-53	38	264	AtTET8|AT2G23810
arahy.Tifrunner.gnm1.ann1.XJXR2L.1	A.hypogaea v1.0	3.73E-52	37	259	AtTET8|AT2G23810
arahy.Tifrunner.gnm1.ann1.PNW690.1	A.hypogaea v1.0	6.19E-52	41	229	AtTET8|AT2G23810
Medtr8g066790.1	M.truncatula Mt4.0v1	7.31E-52	41	263	AtTET8|AT2G23810
Lj4g0027264.1	L.japonicus Lj1.0v1	1.11E-51	41	263	AtTET8|AT2G23810
Phvul.002G287500.1.p	P.vulgaris v2.1	1.69E-51	38	253	AtTET8|AT2G23810
arahy.Tifrunner.gnm1.ann1.2A0H1U.1	A.hypogaea v1.0	1.92E-51	37	259	AtTET8|AT2G23810
Lj4g0018138.1	L.japonicus Lj1.0v1	3.74E-51	40	265	AtTET8|AT2G23810
Phvul.003G180300.1.p	P.vulgaris v2.1	6.13E-51	39	266	AtTET8|AT2G23810
Medtr4g112570.1	M.truncatula Mt4.0v1	4.13E-50	41	264	AtTET8|AT2G23810
Glyma.05G205600.1.p	G.max Wm82.a4.v1	9.3E-50	37	253	AtTET8|AT2G23810
Lcu.2RBY.4g075230.1	L.culinaris v1	1E-49	37	253	AtTET8|AT2G23810
arahy.Tifrunner.gnm1.ann1.Z53AND.1	A.hypogaea v1.0	1.85E-49	38	276	AtTET8|AT2G23810
Phvul.002G304100.1.p	P.vulgaris v2.1	2.53E-49	42	263	AtTET8|AT2G23810
arahy.Tifrunner.gnm1.ann1.T23S8A.1	A.hypogaea v1.0	3.53E-49	38	276	AtTET8|AT2G23810
Lcu.2RBY.7g063730.1	L.culinaris v1	1.8E-48	39	262	AtTET8|AT2G23810
Glyma.08G012500.2.p	G.max Wm82.a4.v1	3.53E-48	36	253	AtTET8|AT2G23810
Lj4g0013226.1	L.japonicus Lj1.0v1	3.93E-48	39	253	AtTET8|AT2G23810
Ca_06587	C.arietinum v1.0	7.57E-48	40	263	AtTET8|AT2G23810
Glyma.08G029100.1.p	G.max Wm82.a4.v1	1.36E-46	43	263	AtTET8|AT2G23810
Medtr5g030070.1	M.truncatula Mt4.0v1	5.72E-46	43	263	AtTET8|AT2G23810
Phvul.004G171300.1.p	P.vulgaris v2.1	3.36E-45	39	266	AtTET8|AT2G23810
Medtr8g097280.1	M.truncatula Mt4.0v1	5.39E-45	36	224	AtTET8|AT2G23810
Lj2g0020248.1	L.japonicus Lj1.0v1	1.13E-44	38	224	AtTET8|AT2G23810
Lj1g0003076.1	L.japonicus Lj1.0v1	1.12E-43	37	278	AtTET8|AT2G23810

Glyma.04G144500.1.p	G.max Wm82.a4.v1	1.93E-43	37	265	AtTET8|AT2G23810
Glyma.15G207300.1.p	G.max Wm82.a4.v1	3.4E-43	38	265	AtTET8|AT2G23810
Medtr2g056130.1	M.truncatula Mt4.0v1	6.24E-42	39	265	AtTET8|AT2G23810
Ca_25832	C.arietinum v1.0	6.93E-42	39	273	AtTET8|AT2G23810
Phvul.009G191200.1.p	P.vulgaris v2.1	4.22E-41	35	265	AtTET8|AT2G23810
arahy.Tifrunner.gnm1.ann1.QC8A1V.1	A.hypogaea v1.0	3.86E-40	37	263	AtTET8|AT2G23810
arahy.Tifrunner.gnm1.ann1.EEBB9D.1	A.hypogaea v1.0	3.86E-40	37	263	AtTET8|AT2G23810
Glyma.07G129100.1.p	G.max Wm82.a4.v1	3.91E-39	38	265	AtTET8|AT2G23810
Ca_05724	C.arietinum v1.0	4.82E-38	32	253	AtTET8|AT2G23810
Lcu.2RBY.2g064540.1	L.culinaris v1	4.92E-36	37	263	AtTET8|AT2G23810
arahy.Tifrunner.gnm1.ann1.H32RIC.1	A.hypogaea v1.0	3.94E-33	35	268	AtTET8|AT2G23810
Ca_23646	C.arietinum v1.0	3.86E-27	40	154	AtTET8|AT2G23810
Phvul.003G151800.1.p	P.vulgaris v2.1	1.23E-105	59	266	AtTET9|AT4G30430
Glyma.13G091000.1.p	G.max Wm82.a4.v1	3.08E-102	58	265	AtTET9|AT4G30430
Lj4g0025241.1	L.japonicus Lj1.0v1	9.28E-102	59	262	AtTET9|AT4G30430
arahy.Tifrunner.gnm1.ann1.9I36N1.1	A.hypogaea v1.0	1.37E-100	58	264	AtTET9|AT4G30430
arahy.Tifrunner.gnm1.ann1.V2Z0GB.1	A.hypogaea v1.0	1.37E-100	58	264	AtTET9|AT4G30430
Glyma.17G069300.1.p	G.max Wm82.a4.v1	1.71E-100	58	265	AtTET9|AT4G30430
Lcu.2RBY.7g075810.1	L.culinaris v1	2.04E-98	56	266	AtTET9|AT4G30430
Medtr4g129670.1	M.truncatula Mt4.0v1	3.84E-98	58	265	AtTET9|AT4G30430
Lj6g0020177.1	L.japonicus Lj1.0v1	2.9E-96	58	266	AtTET9|AT4G30430
Phvul.009G207500.1.p	P.vulgaris v2.1	3.27E-92	56	262	AtTET9|AT4G30430
Glyma.04G147100.1.p	G.max Wm82.a4.v1	3.76E-91	58	262	AtTET9|AT4G30430
Ca_03295	C.arietinum v1.0	3.58E-77	56	241	AtTET9|AT4G30430
arahy.Tifrunner.gnm1.ann1.8QTZ72.1	A.hypogaea v1.0	1.71E-72	48	261	AtTET9|AT4G30430
Phvul.004G071900.1.p	P.vulgaris v2.1	5.53E-69	41	270	AtTET9|AT4G30430
arahy.Tifrunner.gnm1.ann1.LZE6LN.1	A.hypogaea v1.0	1.64E-68	43	271	AtTET9|AT4G30430
Phvul.011G072100.1.p	P.vulgaris v2.1	3.8E-68	45	273	AtTET9|AT4G30430
Medtr6g033335.1	M.truncatula Mt4.0v1	2.03E-67	40	270	AtTET9|AT4G30430
arahy.Tifrunner.gnm1.ann1.Z77BB1.1	A.hypogaea v1.0	2.92E-67	40	270	AtTET9|AT4G30430
arahy.Tifrunner.gnm1.ann1.Z4U2NE.1	A.hypogaea v1.0	4.18E-67	40	270	AtTET9|AT4G30430
Lj5g0000955.1	L.japonicus Lj1.0v1	4.28E-67	40	270	AtTET9|AT4G30430
Lj2g0015394.1	L.japonicus Lj1.0v1	9.53E-67	43	262	AtTET9|AT4G30430
Glyma.10G212400.1.p	G.max Wm82.a4.v1	1.49E-66	40	271	AtTET9|AT4G30430
Glyma.16G145400.1.p	G.max Wm82.a4.v1	2.4E-66	40	270	AtTET9|AT4G30430
arahy.Tifrunner.gnm1.ann1.A2QH6A.1	A.hypogaea v1.0	6.33E-66	47	260	AtTET9|AT4G30430
Lj2g0026181.1	L.japonicus Lj1.0v1	8.85E-66	41	270	AtTET9|AT4G30430

Glyma.20G178700.1.p	G.max Wm82.a4.v1	1.6E-65	40	260	AtTET9|AT4G30430
Glyma.02G063700.1.p	G.max Wm82.a4.v1	3.16E-65	39	270	AtTET9|AT4G30430
Phvul.007G093400.1.p	P.vulgaris v2.1	4.93E-65	38	271	AtTET9|AT4G30430
Glyma.12G072100.1.p	G.max Wm82.a4.v1	6.57E-65	44	273	AtTET9|AT4G30430
Glyma.11G152124.1.p	G.max Wm82.a4.v1	1.56E-64	44	273	AtTET9|AT4G30430
Lj3g0001285.1	L.japonicus Lj1.0v1	2.4E-64	44	274	AtTET9|AT4G30430
arahy.Tifrunner.gnm1.ann1.JZ8XUN.1	A.hypogaea v1.0	2.9E-64	44	273	AtTET9|AT4G30430
Glyma.09G205400.1.p	G.max Wm82.a4.v1	3.08E-64	40	258	AtTET9|AT4G30430
Phvul.003G284700.1.p	P.vulgaris v2.1	3.96E-64	41	258	AtTET9|AT4G30430
Glyma.17G069300.2.p	G.max Wm82.a4.v1	6.46E-64	59	169	AtTET9|AT4G30430
Glyma.13G328700.1.p	G.max Wm82.a4.v1	7.1E-64	43	264	AtTET9|AT4G30430
Medtr4g061010.1	M.truncatula Mt4.0v1	8.11E-64	44	273	AtTET9|AT4G30430
arahy.Tifrunner.gnm1.ann1.AUDB51.1	A.hypogaea v1.0	8.65E-64	44	273	AtTET9|AT4G30430
Glyma.01G095700.1.p	G.max Wm82.a4.v1	4.29E-63	39	262	AtTET9|AT4G30430
Lcu.2RBY.1g046720.1	L.culinaris v1	6.52E-63	38	270	AtTET9|AT4G30430
Lcu.2RBY.4g032690.1	L.culinaris v1	7.46E-63	44	273	AtTET9|AT4G30430
Lcu.2RBY.2g046220.1	L.culinaris v1	1.08E-62	39	270	AtTET9|AT4G30430
Ca_08501	C.arietinum v1.0	1.88E-62	39	270	AtTET9|AT4G30430
Ca_14606	C.arietinum v1.0	3.32E-62	43	273	AtTET9|AT4G30430
Lcu.2RBY.7g030120.1	L.culinaris v1	3.69E-60	39	262	AtTET9|AT4G30430
Phvul.002G140500.1.p	P.vulgaris v2.1	6.76E-60	40	257	AtTET9|AT4G30430
Ca_11504	C.arietinum v1.0	1.34E-59	41	262	AtTET9|AT4G30430
arahy.Tifrunner.gnm1.ann1.0ZGE19.1	A.hypogaea v1.0	1.79E-59	40	271	AtTET9|AT4G30430
Lcu.2RBY.5g015480.1	L.culinaris v1	1.93E-59	41	262	AtTET9|AT4G30430
Glyma.17G084000.1.p	G.max Wm82.a4.v1	2.47E-59	37	266	AtTET9|AT4G30430
Phvul.005G137500.1.p	P.vulgaris v2.1	5.48E-59	44	235	AtTET9|AT4G30430
Lj4g0026647.1	L.japonicus Lj1.0v1	7.07E-59	40	262	AtTET9|AT4G30430
Medtr1g095530.1	M.truncatula Mt4.0v1	3.14E-58	38	270	AtTET9|AT4G30430
Ca_05887	C.arietinum v1.0	3.08E-57	40	263	AtTET9|AT4G30430
arahy.Tifrunner.gnm1.ann1.4R894K.1	A.hypogaea v1.0	2.83E-56	36	270	AtTET9|AT4G30430
Medtr8g066790.1	M.truncatula Mt4.0v1	4.39E-56	39	262	AtTET9|AT4G30430
Medtr8g101600.1	M.truncatula Mt4.0v1	1.52E-55	37	263	AtTET9|AT4G30430
arahy.Tifrunner.gnm1.ann1.D9WPRA.1	A.hypogaea v1.0	2.92E-54	36	270	AtTET9|AT4G30430
arahy.Tifrunner.gnm1.ann1.Z53AND.1	A.hypogaea v1.0	6.21E-54	38	272	AtTET9|AT4G30430
arahy.Tifrunner.gnm1.ann1.B859HK.1	A.hypogaea v1.0	3.12E-53	36	259	AtTET9|AT4G30430
arahy.Tifrunner.gnm1.ann1.N28W6S.1	A.hypogaea v1.0	9.81E-53	38	266	AtTET9|AT4G30430
arahy.Tifrunner.gnm1.ann1.EM0Y56.1	A.hypogaea v1.0	1.14E-52	38	266	AtTET9|AT4G30430

Glyma.05G016200.1.p	G.max Wm82.a4.v1	1.69E-52	37	266	AtTET9|AT4G30430
Lcu.2RBY.4g078330.1	L.culinaris v1	4.55E-52	38	263	AtTET9|AT4G30430
Phvul.003G180300.1.p	P.vulgaris v2.1	4.72E-52	39	263	AtTET9|AT4G30430
Medtr4g112570.1	M.truncatula Mt4.0v1	4.84E-52	39	267	AtTET9|AT4G30430
arahy.Tifrunner.gnm1.ann1.T23S8A.1	A.hypogaea v1.0	1.15E-51	37	272	AtTET9|AT4G30430
arahy.Tifrunner.gnm1.ann1.PNW690.1	A.hypogaea v1.0	6.43E-51	37	230	AtTET9|AT4G30430
Lj4g0027264.1	L.japonicus Lj1.0v1	1.4E-50	38	263	AtTET9|AT4G30430
Medtr5g030070.1	M.truncatula Mt4.0v1	3.93E-50	41	262	AtTET9|AT4G30430
Lcu.2RBY.7g063730.1	L.culinaris v1	1.55E-49	38	261	AtTET9|AT4G30430
Lj4g0018138.1	L.japonicus Lj1.0v1	3.47E-49	37	263	AtTET9|AT4G30430
Ca_06587	C.arietinum v1.0	1.27E-48	38	261	AtTET9|AT4G30430
arahy.Tifrunner.gnm1.ann1.XJXR2L.1	A.hypogaea v1.0	2.03E-47	34	256	AtTET9|AT4G30430
arahy.Tifrunner.gnm1.ann1.2A0H1U.1	A.hypogaea v1.0	8.2E-47	34	256	AtTET9|AT4G30430
Phvul.002G304100.1.p	P.vulgaris v2.1	3.35E-46	37	263	AtTET9|AT4G30430
Phvul.002G287500.1.p	P.vulgaris v2.1	6.16E-45	34	256	AtTET9|AT4G30430
Glyma.08G029100.1.p	G.max Wm82.a4.v1	6.97E-45	38	264	AtTET9|AT4G30430
arahy.Tifrunner.gnm1.ann1.QC8A1V.1	A.hypogaea v1.0	1.01E-44	34	263	AtTET9|AT4G30430
arahy.Tifrunner.gnm1.ann1.EEBB9D.1	A.hypogaea v1.0	1.01E-44	34	263	AtTET9|AT4G30430
Glyma.05G205600.1.p	G.max Wm82.a4.v1	4.34E-43	34	247	AtTET9|AT4G30430
Lcu.2RBY.4g075230.1	L.culinaris v1	3.66E-42	33	252	AtTET9|AT4G30430
Lj1g0003076.1	L.japonicus Lj1.0v1	6.33E-42	36	278	AtTET9|AT4G30430
Glyma.08G012500.2.p	G.max Wm82.a4.v1	1.25E-41	34	247	AtTET9|AT4G30430
Glyma.15G207300.1.p	G.max Wm82.a4.v1	1.53E-41	35	263	AtTET9|AT4G30430
Glyma.04G144500.1.p	G.max Wm82.a4.v1	1.63E-41	35	263	AtTET9|AT4G30430
Lj2g0020248.1	L.japonicus Lj1.0v1	1.68E-41	34	223	AtTET9|AT4G30430
Phvul.004G171300.1.p	P.vulgaris v2.1	3.57E-41	34	262	AtTET9|AT4G30430
Medtr8g097280.1	M.truncatula Mt4.0v1	5.89E-41	33	221	AtTET9|AT4G30430
Ca_25832	C.arietinum v1.0	8.8E-40	37	262	AtTET9|AT4G30430
Medtr2g056130.1	M.truncatula Mt4.0v1	3.66E-39	35	265	AtTET9|AT4G30430
Lj4g0013226.1	L.japonicus Lj1.0v1	5.5E-38	34	247	AtTET9|AT4G30430
Lcu.2RBY.2g064540.1	L.culinaris v1	2.66E-37	34	264	AtTET9|AT4G30430
Glyma.07G129100.1.p	G.max Wm82.a4.v1	3.11E-37	35	266	AtTET9|AT4G30430
Phvul.009G191200.1.p	P.vulgaris v2.1	1.27E-33	32	264	AtTET9|AT4G30430
Ca_05724	C.arietinum v1.0	5.7E-32	28	247	AtTET9|AT4G30430
arahy.Tifrunner.gnm1.ann1.H32RIC.1	A.hypogaea v1.0	2.2E-26	31	262	AtTET9|AT4G30430
arahy.Tifrunner.gnm1.ann1.8RU08H.1	A.hypogaea v1.0	2.69E-25	29	243	AtTET9|AT4G30430
Phvul.002G287500.1.p	P.vulgaris v2.1	3.8E-107	77	230	AtTET10|AT1G63260

arahy.Tifrunner.gnm1.ann1.XJXR2L.1	A.hypogaea v1.0	8.64E-107	76	230	AtTET10|AT1G63260
arahy.Tifrunner.gnm1.ann1.2A0H1U.1	A.hypogaea v1.0	1.43E-106	76	230	AtTET10|AT1G63260
Glyma.05G205600.1.p	G.max Wm82.a4.v1	3.94E-106	77	230	AtTET10|AT1G63260
Glyma.08G012500.2.p	G.max Wm82.a4.v1	1.14E-105	77	230	AtTET10|AT1G63260
Lcu.2RBY.4g075230.1	L.culinaris v1	1.48E-103	74	230	AtTET10|AT1G63260
Medtr8g097280.1	M.truncatula Mt4.0v1	1.89E-102	73	230	AtTET10|AT1G63260
Lj4g0013226.1	L.japonicus Lj1.0v1	2.48E-102	74	230	AtTET10|AT1G63260
Ca_05724	C.arietinum v1.0	2.79E-92	65	230	AtTET10|AT1G63260
arahy.Tifrunner.gnm1.ann1.H32RIC.1	A.hypogaea v1.0	8.05E-80	60	216	AtTET10|AT1G63260
arahy.Tifrunner.gnm1.ann1.8RU08H.1	A.hypogaea v1.0	7.23E-68	54	216	AtTET10|AT1G63260
Phvul.003G284700.1.p	P.vulgaris v2.1	6.32E-40	36	229	AtTET10|AT1G63260
arahy.Tifrunner.gnm1.ann1.9I36N1.1	A.hypogaea v1.0	2.64E-39	40	221	AtTET10|AT1G63260
arahy.Tifrunner.gnm1.ann1.V2Z0GB.1	A.hypogaea v1.0	2.64E-39	40	221	AtTET10|AT1G63260
Phvul.003G151800.1.p	P.vulgaris v2.1	3.41E-39	40	225	AtTET10|AT1G63260
Glyma.01G095700.1.p	G.max Wm82.a4.v1	3.51E-39	37	229	AtTET10|AT1G63260
Lj4g0026647.1	L.japonicus Lj1.0v1	6.04E-39	35	229	AtTET10|AT1G63260
arahy.Tifrunner.gnm1.ann1.LZE6LN.1	A.hypogaea v1.0	1.11E-38	38	230	AtTET10|AT1G63260
Lj4g0025241.1	L.japonicus Lj1.0v1	1.24E-38	39	228	AtTET10|AT1G63260
Glyma.13G091000.1.p	G.max Wm82.a4.v1	4.21E-38	41	226	AtTET10|AT1G63260
Glyma.17G069300.1.p	G.max Wm82.a4.v1	1.28E-37	41	226	AtTET10|AT1G63260
Phvul.011G072100.1.p	P.vulgaris v2.1	5.8E-37	37	234	AtTET10|AT1G63260
Lcu.2RBY.7g030120.1	L.culinaris v1	1.44E-36	35	229	AtTET10|AT1G63260
Lcu.2RBY.7g075810.1	L.culinaris v1	1.98E-36	36	225	AtTET10|AT1G63260
Ca_14606	C.arietinum v1.0	2.02E-36	35	234	AtTET10|AT1G63260
Medtr8g066790.1	M.truncatula Mt4.0v1	3.54E-36	35	229	AtTET10|AT1G63260
Glyma.09G205400.1.p	G.max Wm82.a4.v1	5.53E-36	34	229	AtTET10|AT1G63260
Phvul.002G140500.1.p	P.vulgaris v2.1	3.45E-35	34	229	AtTET10|AT1G63260
Lcu.2RBY.5g015480.1	L.culinaris v1	1.12E-34	34	229	AtTET10|AT1G63260
Lcu.2RBY.4g032690.1	L.culinaris v1	1.18E-34	36	234	AtTET10|AT1G63260
Medtr4g061010.1	M.truncatula Mt4.0v1	1.61E-34	35	234	AtTET10|AT1G63260
Lj2g0015394.1	L.japonicus Lj1.0v1	2.75E-34	35	229	AtTET10|AT1G63260
arahy.Tifrunner.gnm1.ann1.JZ8XUN.1	A.hypogaea v1.0	3.62E-34	33	234	AtTET10|AT1G63260
Ca_11504	C.arietinum v1.0	9.16E-34	34	229	AtTET10|AT1G63260
Lj6g0020177.1	L.japonicus Lj1.0v1	9.58E-34	38	213	AtTET10|AT1G63260
arahy.Tifrunner.gnm1.ann1.AUDB51.1	A.hypogaea v1.0	1.15E-33	33	234	AtTET10|AT1G63260
Glyma.12G072100.1.p	G.max Wm82.a4.v1	1.2E-33	35	234	AtTET10|AT1G63260
Ca_05887	C.arietinum v1.0	5.45E-33	34	229	AtTET10|AT1G63260

Phvul.009G207500.1.p	P.vulgaris v2.1	9.37E-33	38	221	AtTET10|AT1G63260
Medtr4g129670.1	M.truncatula Mt4.0v1	1.62E-32	35	231	AtTET10|AT1G63260
Phvul.005G137500.1.p	P.vulgaris v2.1	1.95E-32	32	234	AtTET10|AT1G63260
Medtr5g030070.1	M.truncatula Mt4.0v1	2.59E-32	33	229	AtTET10|AT1G63260
Lj3g0001285.1	L.japonicus Lj1.0v1	3.38E-32	35	235	AtTET10|AT1G63260
Glyma.13G328700.1.p	G.max Wm82.a4.v1	4.38E-32	35	235	AtTET10|AT1G63260
Glyma.11G152124.1.p	G.max Wm82.a4.v1	1.68E-31	35	234	AtTET10|AT1G63260
arahy.Tifrunner.gnm1.ann1.PNW690.1	A.hypogaea v1.0	2.66E-31	33	229	AtTET10|AT1G63260
Lj5g0000955.1	L.japonicus Lj1.0v1	7.69E-31	36	225	AtTET10|AT1G63260
Lj4g0027264.1	L.japonicus Lj1.0v1	1.55E-30	33	229	AtTET10|AT1G63260
Medtr8g101600.1	M.truncatula Mt4.0v1	2.41E-30	31	229	AtTET10|AT1G63260
arahy.Tifrunner.gnm1.ann1.B859HK.1	A.hypogaea v1.0	1.09E-29	32	229	AtTET10|AT1G63260
Lcu.2RBY.1g046720.1	L.culinaris v1	2.23E-29	36	225	AtTET10|AT1G63260
Phvul.002G304100.1.p	P.vulgaris v2.1	2.32E-29	33	229	AtTET10|AT1G63260
Glyma.04G147100.1.p	G.max Wm82.a4.v1	3.63E-29	37	212	AtTET10|AT1G63260
Glyma.10G212400.1.p	G.max Wm82.a4.v1	4.05E-29	34	225	AtTET10|AT1G63260
Glyma.08G029100.1.p	G.max Wm82.a4.v1	5.19E-29	33	229	AtTET10|AT1G63260
Ca_08501	C.arietinum v1.0	5.72E-29	36	225	AtTET10|AT1G63260
Glyma.20G178700.1.p	G.max Wm82.a4.v1	5.84E-29	34	225	AtTET10|AT1G63260
arahy.Tifrunner.gnm1.ann1.0ZGE19.1	A.hypogaea v1.0	6.81E-29	35	230	AtTET10|AT1G63260
arahy.Tifrunner.gnm1.ann1.Z4U2NE.1	A.hypogaea v1.0	1.1E-28	34	227	AtTET10|AT1G63260
arahy.Tifrunner.gnm1.ann1.Z77BB1.1	A.hypogaea v1.0	1.2E-28	34	227	AtTET10|AT1G63260
Glyma.02G063700.1.p	G.max Wm82.a4.v1	1.34E-28	33	225	AtTET10|AT1G63260
Phvul.007G093400.1.p	P.vulgaris v2.1	2.11E-28	33	225	AtTET10|AT1G63260
Lcu.2RBY.4g078330.1	L.culinaris v1	2.41E-28	33	229	AtTET10|AT1G63260
Phvul.004G071900.1.p	P.vulgaris v2.1	2.85E-28	32	222	AtTET10|AT1G63260
Medtr6g033335.1	M.truncatula Mt4.0v1	2.91E-28	33	222	AtTET10|AT1G63260
Lj2g0026181.1	L.japonicus Lj1.0v1	1.14E-27	33	225	AtTET10|AT1G63260
Glyma.16G145400.1.p	G.max Wm82.a4.v1	1.26E-27	32	225	AtTET10|AT1G63260
arahy.Tifrunner.gnm1.ann1.N28W6S.1	A.hypogaea v1.0	1.79E-27	32	228	AtTET10|AT1G63260
arahy.Tifrunner.gnm1.ann1.EM0Y56.1	A.hypogaea v1.0	1.99E-27	32	228	AtTET10|AT1G63260
Ca_03295	C.arietinum v1.0	3.75E-26	39	207	AtTET10|AT1G63260
Phvul.004G171300.1.p	P.vulgaris v2.1	2.73E-25	35	137	AtTET10|AT1G63260
arahy.Tifrunner.gnm1.ann1.8QTZ72.1	A.hypogaea v1.0	8.07E-25	34	231	AtTET10|AT1G63260
Lcu.2RBY.7g063730.1	L.culinaris v1	9.53E-25	34	138	AtTET10|AT1G63260
Lj2g0020248.1	L.japonicus Lj1.0v1	2.41E-24	33	227	AtTET10|AT1G63260
Lcu.2RBY.2g046220.1	L.culinaris v1	3E-24	34	222	AtTET10|AT1G63260

Medtr1g095530.1	M.truncatula Mt4.0v1	3.39E-24	33	210	AtTET10|AT1G63260
Glyma.05G016200.1.p	G.max Wm82.a4.v1	4.29E-24	31	229	AtTET10|AT1G63260
Ca_06587	C.arietinum v1.0	1.33E-23	34	137	AtTET10|AT1G63260
Medtr4g112570.1	M.truncatula Mt4.0v1	5.6E-23	33	139	AtTET10|AT1G63260
Glyma.17G084000.1.p	G.max Wm82.a4.v1	4.06E-22	31	229	AtTET10|AT1G63260
Lj4g0018138.1	L.japonicus Lj1.0v1	1.14E-21	32	139	AtTET10|AT1G63260
arahy.Tifrunner.gnm1.ann1.A2QH6A.1	A.hypogaea v1.0	1.19E-21	34	232	AtTET10|AT1G63260
Glyma.04G144500.1.p	G.max Wm82.a4.v1	1.9E-21	27	217	AtTET10|AT1G63260
Phvul.003G180300.1.p	P.vulgaris v2.1	2.13E-21	30	228	AtTET10|AT1G63260
Glyma.15G207300.1.p	G.max Wm82.a4.v1	2.2E-21	27	217	AtTET10|AT1G63260
arahy.Tifrunner.gnm1.ann1.T23S8A.1	A.hypogaea v1.0	2.54E-21	33	142	AtTET10|AT1G63260
arahy.Tifrunner.gnm1.ann1.Z53AND.1	A.hypogaea v1.0	9.64E-21	32	142	AtTET10|AT1G63260
Glyma.07G129100.1.p	G.max Wm82.a4.v1	2.58E-20	33	136	AtTET10|AT1G63260
arahy.Tifrunner.gnm1.ann1.K1NV0H.1	A.hypogaea v1.0	3.11E-20	44	86	AtTET10|AT1G63260
arahy.Tifrunner.gnm1.ann1.QC8A1V.1	A.hypogaea v1.0	6.12E-19	30	229	AtTET10|AT1G63260
arahy.Tifrunner.gnm1.ann1.EEBB9D.1	A.hypogaea v1.0	6.12E-19	30	229	AtTET10|AT1G63260
Glyma.17G069300.2.p	G.max Wm82.a4.v1	7.82E-19	38	182	AtTET10|AT1G63260
Ca_23646	C.arietinum v1.0	5.7E-18	35	120	AtTET10|AT1G63260
arahy.Tifrunner.gnm1.ann1.4R894K.1	A.hypogaea v1.0	6.04E-18	32	225	AtTET10|AT1G63260
arahy.Tifrunner.gnm1.ann1.D9WPRA.1	A.hypogaea v1.0	1.15E-16	32	225	AtTET10|AT1G63260
Medtr2g056130.1	M.truncatula Mt4.0v1	3.7E-16	31	137	AtTET10|AT1G63260
Lj1g0003076.1	L.japonicus Lj1.0v1	1.08E-15	28	126	AtTET10|AT1G63260
Glyma.17G084000.1.p	G.max Wm82.a4.v1	4.78E-74	47	267	AtTET11|AT1G18520
Phvul.003G180300.1.p	P.vulgaris v2.1	5.34E-71	46	267	AtTET11|AT1G18520
Lcu.2RBY.7g063730.1	L.culinaris v1	2.85E-70	47	264	AtTET11|AT1G18520
arahy.Tifrunner.gnm1.ann1.T23S8A.1	A.hypogaea v1.0	3.21E-70	44	266	AtTET11|AT1G18520
Lj4g0018138.1	L.japonicus Lj1.0v1	3.49E-70	47	266	AtTET11|AT1G18520
Glyma.05G016200.1.p	G.max Wm82.a4.v1	1.24E-69	46	267	AtTET11|AT1G18520
Ca_06587	C.arietinum v1.0	3.65E-69	47	273	AtTET11|AT1G18520
arahy.Tifrunner.gnm1.ann1.Z53AND.1	A.hypogaea v1.0	5.34E-69	44	266	AtTET11|AT1G18520
Medtr4g112570.1	M.truncatula Mt4.0v1	6.04E-69	45	271	AtTET11|AT1G18520
Phvul.003G151800.1.p	P.vulgaris v2.1	2.14E-68	44	264	AtTET11|AT1G18520
Glyma.13G091000.1.p	G.max Wm82.a4.v1	3.68E-68	45	264	AtTET11|AT1G18520
Lj4g0025241.1	L.japonicus Lj1.0v1	8.36E-68	44	264	AtTET11|AT1G18520
arahy.Tifrunner.gnm1.ann1.9I36N1.1	A.hypogaea v1.0	1E-67	44	264	AtTET11|AT1G18520
arahy.Tifrunner.gnm1.ann1.V2Z0GB.1	A.hypogaea v1.0	1E-67	44	264	AtTET11|AT1G18520
Glyma.17G069300.1.p	G.max Wm82.a4.v1	4.64E-67	45	264	AtTET11|AT1G18520

Lcu.2RBY.7g075810.1	L.culinaris v1	1.19E-64	42	264	AtTET11|AT1G18520
Medtr4g129670.1	M.truncatula Mt4.0v1	1.02E-63	42	262	AtTET11|AT1G18520
Lj6g0020177.1	L.japonicus Lj1.0v1	1.32E-62	43	264	AtTET11|AT1G18520
arahy.Tifrunner.gnm1.ann1.LZE6LN.1	A.hypogaea v1.0	1.18E-60	40	263	AtTET11|AT1G18520
Glyma.04G147100.1.p	G.max Wm82.a4.v1	7.57E-60	42	261	AtTET11|AT1G18520
Lcu.2RBY.7g030120.1	L.culinaris v1	1.04E-58	39	262	AtTET11|AT1G18520
Glyma.12G072100.1.p	G.max Wm82.a4.v1	1.11E-58	41	267	AtTET11|AT1G18520
Medtr2g056130.1	M.truncatula Mt4.0v1	2.02E-58	43	268	AtTET11|AT1G18520
Phvul.009G207500.1.p	P.vulgaris v2.1	6.07E-58	42	269	AtTET11|AT1G18520
Ca_03295	C.arietinum v1.0	2.58E-57	44	232	AtTET11|AT1G18520
Glyma.04G144500.1.p	G.max Wm82.a4.v1	3.46E-57	43	265	AtTET11|AT1G18520
Phvul.011G072100.1.p	P.vulgaris v2.1	3.73E-57	38	265	AtTET11|AT1G18520
Glyma.11G152124.1.p	G.max Wm82.a4.v1	1.52E-56	40	265	AtTET11|AT1G18520
Glyma.15G207300.1.p	G.max Wm82.a4.v1	2.42E-56	43	265	AtTET11|AT1G18520
Lj2g0015394.1	L.japonicus Lj1.0v1	2.61E-56	38	263	AtTET11|AT1G18520
Lj5g0000955.1	L.japonicus Lj1.0v1	5.12E-56	40	267	AtTET11|AT1G18520
Glyma.10G212400.1.p	G.max Wm82.a4.v1	5.23E-56	40	267	AtTET11|AT1G18520
Medtr4g061010.1	M.truncatula Mt4.0v1	1.51E-55	38	236	AtTET11|AT1G18520
Glyma.01G095700.1.p	G.max Wm82.a4.v1	2.58E-55	40	262	AtTET11|AT1G18520
Ca_14606	C.arietinum v1.0	1.86E-54	38	267	AtTET11|AT1G18520
arahy.Tifrunner.gnm1.ann1.JZ8XUN.1	A.hypogaea v1.0	2.57E-54	38	265	AtTET11|AT1G18520
arahy.Tifrunner.gnm1.ann1.AUDB51.1	A.hypogaea v1.0	2.78E-54	38	265	AtTET11|AT1G18520
Glyma.16G145400.1.p	G.max Wm82.a4.v1	3.15E-54	39	267	AtTET11|AT1G18520
Ca_08501	C.arietinum v1.0	3.83E-54	39	267	AtTET11|AT1G18520
Medtr8g066790.1	M.truncatula Mt4.0v1	4.27E-54	40	264	AtTET11|AT1G18520
Lcu.2RBY.4g032690.1	L.culinaris v1	4.61E-54	38	265	AtTET11|AT1G18520
Glyma.13G328700.1.p	G.max Wm82.a4.v1	5.09E-54	37	266	AtTET11|AT1G18520
Phvul.007G093400.1.p	P.vulgaris v2.1	6.02E-54	38	267	AtTET11|AT1G18520
Lj2g0026181.1	L.japonicus Lj1.0v1	6.27E-54	38	267	AtTET11|AT1G18520
Lj1g0003076.1	L.japonicus Lj1.0v1	6.31E-54	42	272	AtTET11|AT1G18520
Glyma.20G178700.1.p	G.max Wm82.a4.v1	2.12E-53	38	267	AtTET11|AT1G18520
Lj3g0001285.1	L.japonicus Lj1.0v1	2.24E-53	38	268	AtTET11|AT1G18520
Glyma.02G063700.1.p	G.max Wm82.a4.v1	2.69E-53	39	267	AtTET11|AT1G18520
arahy.Tifrunner.gnm1.ann1.Z4U2NE.1	A.hypogaea v1.0	3.9E-53	39	267	AtTET11|AT1G18520
Lcu.2RBY.1g046720.1	L.culinaris v1	1.46E-52	39	267	AtTET11|AT1G18520
Phvul.003G284700.1.p	P.vulgaris v2.1	1.67E-52	38	262	AtTET11|AT1G18520
Ca_25832	C.arietinum v1.0	2.62E-52	41	265	AtTET11|AT1G18520

Glyma.09G205400.1.p	G.max Wm82.a4.v1	2.96E-52	38	262	AtTET11|AT1G18520
Phvul.004G071900.1.p	P.vulgaris v2.1	3.65E-52	37	267	AtTET11|AT1G18520
Medtr6g033335.1	M.truncatula Mt4.0v1	6.45E-52	37	271	AtTET11|AT1G18520
arahy.Tifrunner.gnm1.ann1.Z77BB1.1	A.hypogaea v1.0	7.01E-52	38	268	AtTET11|AT1G18520
Lcu.2RBY.5g015480.1	L.culinaris v1	7.48E-52	38	262	AtTET11|AT1G18520
Lj4g0026647.1	L.japonicus Lj1.0v1	4.72E-51	38	262	AtTET11|AT1G18520
Lcu.2RBY.2g064540.1	L.culinaris v1	5.3E-51	41	266	AtTET11|AT1G18520
Phvul.005G137500.1.p	P.vulgaris v2.1	7.57E-51	38	240	AtTET11|AT1G18520
Ca_11504	C.arietinum v1.0	2.04E-50	38	262	AtTET11|AT1G18520
Lcu.2RBY.2g046220.1	L.culinaris v1	2.93E-50	37	267	AtTET11|AT1G18520
Medtr1g095530.1	M.truncatula Mt4.0v1	3.93E-50	40	267	AtTET11|AT1G18520
arahy.Tifrunner.gnm1.ann1.8QTZ72.1	A.hypogaea v1.0	6.03E-49	34	263	AtTET11|AT1G18520
arahy.Tifrunner.gnm1.ann1.B859HK.1	A.hypogaea v1.0	7.48E-49	37	264	AtTET11|AT1G18520
arahy.Tifrunner.gnm1.ann1.PNW690.1	A.hypogaea v1.0	8.02E-49	38	233	AtTET11|AT1G18520
Glyma.05G205600.1.p	G.max Wm82.a4.v1	2.73E-48	33	258	AtTET11|AT1G18520
Ca_05887	C.arietinum v1.0	3.72E-48	38	262	AtTET11|AT1G18520
Phvul.009G191200.1.p	P.vulgaris v2.1	1.82E-47	38	265	AtTET11|AT1G18520
Medtr8g101600.1	M.truncatula Mt4.0v1	2.01E-47	37	262	AtTET11|AT1G18520
Lcu.2RBY.4g075230.1	L.culinaris v1	3.89E-47	33	258	AtTET11|AT1G18520
Phvul.002G287500.1.p	P.vulgaris v2.1	1.31E-46	33	258	AtTET11|AT1G18520
Glyma.08G012500.2.p	G.max Wm82.a4.v1	1.93E-46	33	258	AtTET11|AT1G18520
arahy.Tifrunner.gnm1.ann1.0ZGE19.1	A.hypogaea v1.0	3.8E-46	34	263	AtTET11|AT1G18520
Medtr5g030070.1	M.truncatula Mt4.0v1	7.69E-45	38	262	AtTET11|AT1G18520
Phvul.002G140500.1.p	P.vulgaris v2.1	1.16E-44	35	261	AtTET11|AT1G18520
arahy.Tifrunner.gnm1.ann1.A2QH6A.1	A.hypogaea v1.0	2.34E-44	34	263	AtTET11|AT1G18520
Lj4g0027264.1	L.japonicus Lj1.0v1	5.07E-44	37	267	AtTET11|AT1G18520
arahy.Tifrunner.gnm1.ann1.XJXR2L.1	A.hypogaea v1.0	7.88E-44	31	267	AtTET11|AT1G18520
Lcu.2RBY.4g078330.1	L.culinaris v1	1.53E-43	36	262	AtTET11|AT1G18520
arahy.Tifrunner.gnm1.ann1.N28W6S.1	A.hypogaea v1.0	1.62E-43	35	265	AtTET11|AT1G18520
arahy.Tifrunner.gnm1.ann1.EM0Y56.1	A.hypogaea v1.0	1.95E-43	35	265	AtTET11|AT1G18520
arahy.Tifrunner.gnm1.ann1.2A0H1U.1	A.hypogaea v1.0	3.69E-43	31	267	AtTET11|AT1G18520
arahy.Tifrunner.gnm1.ann1.4R894K.1	A.hypogaea v1.0	8.2E-43	34	267	AtTET11|AT1G18520
Lj4g0013226.1	L.japonicus Lj1.0v1	1.85E-42	33	258	AtTET11|AT1G18520
Medtr8g097280.1	M.truncatula Mt4.0v1	1.99E-42	31	232	AtTET11|AT1G18520
Glyma.08G029100.1.p	G.max Wm82.a4.v1	1.1E-41	37	267	AtTET11|AT1G18520
Phvul.002G304100.1.p	P.vulgaris v2.1	1.3E-40	37	262	AtTET11|AT1G18520
arahy.Tifrunner.gnm1.ann1.D9WPRA.1	A.hypogaea v1.0	1.22E-39	34	267	AtTET11|AT1G18520

Phvul.004G171300.1.p	P.vulgaris v2.1	3.15E-37	36	262	AtTET11|AT1G18520
arahy.Tifrunner.gnm1.ann1.QC8A1V.1	A.hypogaea v1.0	1.42E-36	31	261	AtTET11|AT1G18520
arahy.Tifrunner.gnm1.ann1.EEBB9D.1	A.hypogaea v1.0	1.42E-36	31	261	AtTET11|AT1G18520
Lj2g0020248.1	L.japonicus Lj1.0v1	5.2E-36	34	231	AtTET11|AT1G18520
Glyma.07G129100.1.p	G.max Wm82.a4.v1	9.85E-36	35	266	AtTET11|AT1G18520
Glyma.17G069300.2.p	G.max Wm82.a4.v1	3.4E-33	41	170	AtTET11|AT1G18520
Ca_05724	C.arietinum v1.0	1.32E-30	29	258	AtTET11|AT1G18520
arahy.Tifrunner.gnm1.ann1.H32RIC.1	A.hypogaea v1.0	8.13E-29	36	179	AtTET11|AT1G18520
Ca_23646	C.arietinum v1.0	1E-25	40	156	AtTET11|AT1G18520
arahy.Tifrunner.gnm1.ann1.K1NV0H.1	A.hypogaea v1.0	2.61E-24	48	81	AtTET11|AT1G18520
Lcu.2RBY.7g063730.1	L.culinaris v1	2.1E-40	40	260	AtTET12|AT5G23030
Medtr4g112570.1	M.truncatula Mt4.0v1	3.24E-40	40	260	AtTET12|AT5G23030
Phvul.003G180300.1.p	P.vulgaris v2.1	2.98E-39	41	260	AtTET12|AT5G23030
Ca_06587	C.arietinum v1.0	3.28E-39	40	260	AtTET12|AT5G23030
Glyma.17G084000.1.p	G.max Wm82.a4.v1	9.77E-39	38	261	AtTET12|AT5G23030
Lj4g0018138.1	L.japonicus Lj1.0v1	1.8E-38	39	266	AtTET12|AT5G23030
arahy.Tifrunner.gnm1.ann1.T23S8A.1	A.hypogaea v1.0	4.03E-35	35	271	AtTET12|AT5G23030
arahy.Tifrunner.gnm1.ann1.Z53AND.1	A.hypogaea v1.0	8.1E-35	36	266	AtTET12|AT5G23030
Glyma.05G016200.1.p	G.max Wm82.a4.v1	9.22E-35	39	261	AtTET12|AT5G23030
Glyma.17G069300.1.p	G.max Wm82.a4.v1	7.06E-34	36	261	AtTET12|AT5G23030
Lcu.2RBY.7g075810.1	L.culinaris v1	1.24E-33	36	267	AtTET12|AT5G23030
Phvul.003G151800.1.p	P.vulgaris v2.1	9.79E-33	36	267	AtTET12|AT5G23030
Glyma.13G091000.1.p	G.max Wm82.a4.v1	1.18E-31	35	261	AtTET12|AT5G23030
Lj6g0020177.1	L.japonicus Lj1.0v1	6.71E-30	34	267	AtTET12|AT5G23030
Medtr4g129670.1	M.truncatula Mt4.0v1	9.7E-30	34	266	AtTET12|AT5G23030
arahy.Tifrunner.gnm1.ann1.9I36N1.1	A.hypogaea v1.0	1.88E-29	35	261	AtTET12|AT5G23030
arahy.Tifrunner.gnm1.ann1.V2Z0GB.1	A.hypogaea v1.0	1.88E-29	35	261	AtTET12|AT5G23030
Medtr2g056130.1	M.truncatula Mt4.0v1	3.06E-29	35	256	AtTET12|AT5G23030
Lj4g0025241.1	L.japonicus Lj1.0v1	4.19E-29	34	269	AtTET12|AT5G23030
Glyma.04G144500.1.p	G.max Wm82.a4.v1	5.99E-29	34	264	AtTET12|AT5G23030
Glyma.15G207300.1.p	G.max Wm82.a4.v1	9.6E-29	34	264	AtTET12|AT5G23030
Ca_03295	C.arietinum v1.0	3.71E-28	36	240	AtTET12|AT5G23030
Glyma.04G147100.1.p	G.max Wm82.a4.v1	6.53E-28	33	264	AtTET12|AT5G23030
Lj1g0003076.1	L.japonicus Lj1.0v1	1.66E-27	34	268	AtTET12|AT5G23030
Phvul.009G191200.1.p	P.vulgaris v2.1	3.98E-27	33	261	AtTET12|AT5G23030
arahy.Tifrunner.gnm1.ann1.A2QH6A.1	A.hypogaea v1.0	5.13E-27	37	259	AtTET12|AT5G23030
Ca_25832	C.arietinum v1.0	7.41E-27	35	224	AtTET12|AT5G23030

arahy.Tifrunner.gnm1.ann1.8QTZ72.1	A.hypogaea v1.0	1.99E-26	35	260	AtTET12|AT5G23030
arahy.Tifrunner.gnm1.ann1.LZE6LN.1	A.hypogaea v1.0	4.16E-26	34	227	AtTET12|AT5G23030
Phvul.005G137500.1.p	P.vulgaris v2.1	9.37E-26	34	203	AtTET12|AT5G23030
Ca_14606	C.arietinum v1.0	2.44E-25	36	200	AtTET12|AT5G23030
Phvul.009G207500.1.p	P.vulgaris v2.1	2.62E-25	34	260	AtTET12|AT5G23030
arahy.Tifrunner.gnm1.ann1.AUDB51.1	A.hypogaea v1.0	2.76E-25	38	201	AtTET12|AT5G23030
Lcu.2RBY.4g032690.1	L.culinaris v1	6.2E-25	35	200	AtTET12|AT5G23030
Glyma.12G072100.1.p	G.max Wm82.a4.v1	1.15E-24	36	200	AtTET12|AT5G23030
arahy.Tifrunner.gnm1.ann1.JZ8XUN.1	A.hypogaea v1.0	1.35E-24	37	201	AtTET12|AT5G23030
Phvul.011G072100.1.p	P.vulgaris v2.1	1.35E-24	33	228	AtTET12|AT5G23030
Lcu.2RBY.7g030120.1	L.culinaris v1	1.84E-24	30	260	AtTET12|AT5G23030
Glyma.11G152124.1.p	G.max Wm82.a4.v1	4.9E-24	35	203	AtTET12|AT5G23030
Glyma.13G328700.1.p	G.max Wm82.a4.v1	4.31E-23	35	228	AtTET12|AT5G23030
Medtr1g095530.1	M.truncatula Mt4.0v1	9.4E-23	32	222	AtTET12|AT5G23030
Glyma.01G095700.1.p	G.max Wm82.a4.v1	1.89E-22	29	258	AtTET12|AT5G23030
Medtr4g061010.1	M.truncatula Mt4.0v1	2.19E-22	33	203	AtTET12|AT5G23030
Lj5g0000955.1	L.japonicus Lj1.0v1	2.27E-22	31	222	AtTET12|AT5G23030
Lj3g0001285.1	L.japonicus Lj1.0v1	2.76E-22	35	202	AtTET12|AT5G23030
arahy.Tifrunner.gnm1.ann1.PNW690.1	A.hypogaea v1.0	4.12E-22	33	204	AtTET12|AT5G23030
Glyma.10G212400.1.p	G.max Wm82.a4.v1	4.21E-22	28	257	AtTET12|AT5G23030
Glyma.20G178700.1.p	G.max Wm82.a4.v1	4.91E-22	29	257	AtTET12|AT5G23030
Phvul.007G093400.1.p	P.vulgaris v2.1	5.12E-22	29	257	AtTET12|AT5G23030
arahy.Tifrunner.gnm1.ann1.N28W6S.1	A.hypogaea v1.0	7.28E-22	31	230	AtTET12|AT5G23030
arahy.Tifrunner.gnm1.ann1.EM0Y56.1	A.hypogaea v1.0	7.51E-22	31	230	AtTET12|AT5G23030
Lcu.2RBY.1g046720.1	L.culinaris v1	8.72E-22	29	257	AtTET12|AT5G23030
Ca_05887	C.arietinum v1.0	1.04E-21	31	256	AtTET12|AT5G23030
Phvul.003G284700.1.p	P.vulgaris v2.1	1.11E-21	28	258	AtTET12|AT5G23030
Phvul.004G071900.1.p	P.vulgaris v2.1	1.82E-21	31	222	AtTET12|AT5G23030
Ca_08501	C.arietinum v1.0	1.98E-21	28	257	AtTET12|AT5G23030
Medtr6g033335.1	M.truncatula Mt4.0v1	2.38E-21	28	257	AtTET12|AT5G23030
Lj2g0026181.1	L.japonicus Lj1.0v1	5.89E-21	30	222	AtTET12|AT5G23030
arahy.Tifrunner.gnm1.ann1.B859HK.1	A.hypogaea v1.0	1.22E-20	32	204	AtTET12|AT5G23030
arahy.Tifrunner.gnm1.ann1.2A0H1U.1	A.hypogaea v1.0	1.71E-20	32	220	AtTET12|AT5G23030
Medtr8g066790.1	M.truncatula Mt4.0v1	1.88E-20	34	165	AtTET12|AT5G23030
Glyma.16G145400.1.p	G.max Wm82.a4.v1	2.03E-20	30	220	AtTET12|AT5G23030
Glyma.08G029100.1.p	G.max Wm82.a4.v1	2.67E-20	32	159	AtTET12|AT5G23030
arahy.Tifrunner.gnm1.ann1.XJXR2L.1	A.hypogaea v1.0	2.91E-20	32	220	AtTET12|AT5G23030

Medtr8g101600.1	M.truncatula Mt4.0v1	3.14E-20	32	222	AtTET12|AT5G23030
Lj4g0026647.1	L.japonicus Lj1.0v1	9.18E-20	31	230	AtTET12|AT5G23030
Lj2g0015394.1	L.japonicus Lj1.0v1	9.46E-20	29	226	AtTET12|AT5G23030
Lj4g0013226.1	L.japonicus Lj1.0v1	1.08E-19	31	221	AtTET12|AT5G23030
Phvul.002G287500.1.p	P.vulgaris v2.1	1.15E-19	31	221	AtTET12|AT5G23030
Glyma.07G129100.1.p	G.max Wm82.a4.v1	1.9E-19	32	163	AtTET12|AT5G23030
arahy.Tifrunner.gnm1.ann1.Z4U2NE.1	A.hypogaea v1.0	2.03E-19	28	257	AtTET12|AT5G23030
Lcu.2RBY.2g064540.1	L.culinaris v1	2.22E-19	29	259	AtTET12|AT5G23030
Phvul.004G171300.1.p	P.vulgaris v2.1	2.27E-19	31	159	AtTET12|AT5G23030
Glyma.09G205400.1.p	G.max Wm82.a4.v1	2.67E-19	30	224	AtTET12|AT5G23030
Lcu.2RBY.4g078330.1	L.culinaris v1	3.31E-19	30	256	AtTET12|AT5G23030
Lcu.2RBY.2g046220.1	L.culinaris v1	3.35E-19	28	225	AtTET12|AT5G23030
Glyma.05G205600.1.p	G.max Wm82.a4.v1	3.75E-19	31	220	AtTET12|AT5G23030
Lj4g0027264.1	L.japonicus Lj1.0v1	5.13E-19	33	159	AtTET12|AT5G23030
arahy.Tifrunner.gnm1.ann1.Z77BB1.1	A.hypogaea v1.0	6.32E-19	29	222	AtTET12|AT5G23030
arahy.Tifrunner.gnm1.ann1.0ZGE19.1	A.hypogaea v1.0	6.68E-19	31	224	AtTET12|AT5G23030
Glyma.02G063700.1.p	G.max Wm82.a4.v1	1.04E-18	30	222	AtTET12|AT5G23030
Glyma.08G012500.2.p	G.max Wm82.a4.v1	1.76E-18	30	220	AtTET12|AT5G23030
Phvul.002G304100.1.p	P.vulgaris v2.1	4.72E-18	33	159	AtTET12|AT5G23030
Lj2g0020248.1	L.japonicus Lj1.0v1	8.55E-18	35	130	AtTET12|AT5G23030
Ca_11504	C.arietinum v1.0	9.88E-18	28	230	AtTET12|AT5G23030
Lcu.2RBY.5g015480.1	L.culinaris v1	1.14E-17	28	230	AtTET12|AT5G23030
Phvul.002G140500.1.p	P.vulgaris v2.1	1.52E-17	27	255	AtTET12|AT5G23030
arahy.Tifrunner.gnm1.ann1.H32RIC.1	A.hypogaea v1.0	4.22E-17	31	160	AtTET12|AT5G23030
Lcu.2RBY.4g075230.1	L.culinaris v1	8.78E-17	30	220	AtTET12|AT5G23030
Medtr8g097280.1	M.truncatula Mt4.0v1	1.76E-16	29	197	AtTET12|AT5G23030
Ca_23646	C.arietinum v1.0	3.17E-16	31	143	AtTET12|AT5G23030
arahy.Tifrunner.gnm1.ann1.K1NV0H.1	A.hypogaea v1.0	4.9E-16	51	75	AtTET12|AT5G23030
Ca_05724	C.arietinum v1.0	2.2E-15	29	160	AtTET12|AT5G23030
arahy.Tifrunner.gnm1.ann1.8RU08H.1	A.hypogaea v1.0	3.07E-14	30	157	AtTET12|AT5G23030
Medtr5g030070.1	M.truncatula Mt4.0v1	5.64E-14	32	130	AtTET12|AT5G23030
arahy.Tifrunner.gnm1.ann1.4R894K.1	A.hypogaea v1.0	4.05E-13	27	257	AtTET12|AT5G23030
Glyma.17G069300.2.p	G.max Wm82.a4.v1	6.18E-12	32	169	AtTET12|AT5G23030
arahy.Tifrunner.gnm1.ann1.D9WPRA.1	A.hypogaea v1.0	1.66E-11	27	257	AtTET12|AT5G23030
arahy.Tifrunner.gnm1.ann1.QC8A1V.1	A.hypogaea v1.0	6.73E-11	42	89	AtTET12|AT5G23030
arahy.Tifrunner.gnm1.ann1.LZE6LN.1	A.hypogaea v1.0	1.36E-38	29	245	AtTET13|AT2G03840
Phvul.011G072100.1.p	P.vulgaris v2.1	2.71E-38	31	222	AtTET13|AT2G03840

Glyma.12G072100.1.p	G.max Wm82.a4.v1	3.08E-37	32	223	AtTET13|AT2G03840
Glyma.11G152124.1.p	G.max Wm82.a4.v1	1.89E-36	32	223	AtTET13|AT2G03840
arahy.Tifrunner.gnm1.ann1.JZ8XUN.1	A.hypogaea v1.0	6.79E-36	30	223	AtTET13|AT2G03840
arahy.Tifrunner.gnm1.ann1.AUDB51.1	A.hypogaea v1.0	9.84E-36	30	223	AtTET13|AT2G03840
Glyma.04G147100.1.p	G.max Wm82.a4.v1	1.38E-35	29	242	AtTET13|AT2G03840
Lj2g0015394.1	L.japonicus Lj1.0v1	5.21E-35	28	246	AtTET13|AT2G03840
Phvul.003G151800.1.p	P.vulgaris v2.1	5.23E-35	30	251	AtTET13|AT2G03840
Medtr4g061010.1	M.truncatula Mt4.0v1	3.54E-34	30	223	AtTET13|AT2G03840
Glyma.10G212400.1.p	G.max Wm82.a4.v1	3.96E-34	28	243	AtTET13|AT2G03840
Ca_14606	C.arietinum v1.0	4.1E-34	30	223	AtTET13|AT2G03840
Lcu.2RBY.4g032690.1	L.culinaris v1	4.66E-34	30	223	AtTET13|AT2G03840
Ca_08501	C.arietinum v1.0	5E-34	30	244	AtTET13|AT2G03840
Lj5g0000955.1	L.japonicus Lj1.0v1	8.47E-34	28	247	AtTET13|AT2G03840
Lj6g0020177.1	L.japonicus Lj1.0v1	9.9E-34	31	208	AtTET13|AT2G03840
Phvul.005G137500.1.p	P.vulgaris v2.1	1.48E-33	29	223	AtTET13|AT2G03840
Glyma.20G178700.1.p	G.max Wm82.a4.v1	2.16E-33	28	243	AtTET13|AT2G03840
Glyma.09G205400.1.p	G.max Wm82.a4.v1	3.06E-33	30	225	AtTET13|AT2G03840
Lcu.2RBY.7g075810.1	L.culinaris v1	4.45E-33	29	241	AtTET13|AT2G03840
arahy.Tifrunner.gnm1.ann1.Z4U2NE.1	A.hypogaea v1.0	7.89E-33	30	227	AtTET13|AT2G03840
Glyma.13G091000.1.p	G.max Wm82.a4.v1	9E-33	29	265	AtTET13|AT2G03840
Glyma.13G328700.1.p	G.max Wm82.a4.v1	9.86E-33	29	246	AtTET13|AT2G03840
Lj3g0001285.1	L.japonicus Lj1.0v1	1.15E-32	29	224	AtTET13|AT2G03840
Lcu.2RBY.5g015480.1	L.culinaris v1	2.02E-32	30	221	AtTET13|AT2G03840
arahy.Tifrunner.gnm1.ann1.Z77BB1.1	A.hypogaea v1.0	2.71E-32	30	226	AtTET13|AT2G03840
Medtr6g033335.1	M.truncatula Mt4.0v1	4.89E-32	28	224	AtTET13|AT2G03840
Ca_11504	C.arietinum v1.0	4.9E-32	31	212	AtTET13|AT2G03840
Lj4g0025241.1	L.japonicus Lj1.0v1	1.7E-31	30	223	AtTET13|AT2G03840
Lj2g0026181.1	L.japonicus Lj1.0v1	1.89E-31	29	237	AtTET13|AT2G03840
Glyma.02G063700.1.p	G.max Wm82.a4.v1	2.98E-31	30	233	AtTET13|AT2G03840
Medtr4g129670.1	M.truncatula Mt4.0v1	2.98E-31	31	233	AtTET13|AT2G03840
Ca_05887	C.arietinum v1.0	4.42E-31	27	230	AtTET13|AT2G03840
Phvul.007G093400.1.p	P.vulgaris v2.1	5.65E-31	26	243	AtTET13|AT2G03840
Phvul.003G284700.1.p	P.vulgaris v2.1	7.41E-31	28	225	AtTET13|AT2G03840
Glyma.01G095700.1.p	G.max Wm82.a4.v1	9.84E-31	29	225	AtTET13|AT2G03840
Medtr8g066790.1	M.truncatula Mt4.0v1	2.1E-30	28	225	AtTET13|AT2G03840
Lcu.2RBY.7g030120.1	L.culinaris v1	3.77E-30	27	245	AtTET13|AT2G03840
Glyma.16G145400.1.p	G.max Wm82.a4.v1	6.29E-30	30	232	AtTET13|AT2G03840

Lcu.2RBY.1g046720.1	L.culinaris v1	6.43E-30	27	243	AtTET13|AT2G03840
arahy.Tifrunner.gnm1.ann1.N28W6S.1	A.hypogaea v1.0	7.88E-30	28	230	AtTET13|AT2G03840
Lj4g0026647.1	L.japonicus Lj1.0v1	7.95E-30	29	225	AtTET13|AT2G03840
Lcu.2RBY.4g078330.1	L.culinaris v1	9.41E-30	29	231	AtTET13|AT2G03840
arahy.Tifrunner.gnm1.ann1.EM0Y56.1	A.hypogaea v1.0	9.71E-30	28	230	AtTET13|AT2G03840
arahy.Tifrunner.gnm1.ann1.B859HK.1	A.hypogaea v1.0	1.41E-29	27	231	AtTET13|AT2G03840
arahy.Tifrunner.gnm1.ann1.PNW690.1	A.hypogaea v1.0	2.09E-29	27	231	AtTET13|AT2G03840
arahy.Tifrunner.gnm1.ann1.9I36N1.1	A.hypogaea v1.0	2.52E-29	27	239	AtTET13|AT2G03840
arahy.Tifrunner.gnm1.ann1.V2Z0GB.1	A.hypogaea v1.0	2.52E-29	27	239	AtTET13|AT2G03840
Medtr1g095530.1	M.truncatula Mt4.0v1	4.54E-29	28	229	AtTET13|AT2G03840
Glyma.17G069300.1.p	G.max Wm82.a4.v1	5.2E-29	28	268	AtTET13|AT2G03840
Phvul.009G207500.1.p	P.vulgaris v2.1	5.94E-29	28	259	AtTET13|AT2G03840
Lcu.2RBY.2g046220.1	L.culinaris v1	1.25E-28	29	225	AtTET13|AT2G03840
arahy.Tifrunner.gnm1.ann1.8QTZ72.1	A.hypogaea v1.0	1.48E-28	30	217	AtTET13|AT2G03840
Phvul.004G071900.1.p	P.vulgaris v2.1	1.66E-28	29	232	AtTET13|AT2G03840
Medtr8g101600.1	M.truncatula Mt4.0v1	1.66E-28	27	230	AtTET13|AT2G03840
Phvul.002G140500.1.p	P.vulgaris v2.1	3.38E-28	27	226	AtTET13|AT2G03840
arahy.Tifrunner.gnm1.ann1.0ZGE19.1	A.hypogaea v1.0	5.16E-28	25	244	AtTET13|AT2G03840
Medtr8g097280.1	M.truncatula Mt4.0v1	5.85E-28	30	186	AtTET13|AT2G03840
arahy.Tifrunner.gnm1.ann1.A2QH6A.1	A.hypogaea v1.0	1.12E-27	29	216	AtTET13|AT2G03840
Medtr5g030070.1	M.truncatula Mt4.0v1	1.47E-27	30	225	AtTET13|AT2G03840
Lcu.2RBY.4g075230.1	L.culinaris v1	1.99E-27	31	188	AtTET13|AT2G03840
Phvul.002G304100.1.p	P.vulgaris v2.1	1.25E-26	27	230	AtTET13|AT2G03840
Phvul.002G287500.1.p	P.vulgaris v2.1	4.14E-26	30	210	AtTET13|AT2G03840
Lj4g0027264.1	L.japonicus Lj1.0v1	4.88E-26	27	230	AtTET13|AT2G03840
Ca_03295	C.arietinum v1.0	1.15E-25	31	193	AtTET13|AT2G03840
Glyma.08G029100.1.p	G.max Wm82.a4.v1	1.32E-25	28	230	AtTET13|AT2G03840
Ca_06587	C.arietinum v1.0	1.39E-25	27	216	AtTET13|AT2G03840
arahy.Tifrunner.gnm1.ann1.2A0H1U.1	A.hypogaea v1.0	1.46E-25	28	247	AtTET13|AT2G03840
Glyma.05G205600.1.p	G.max Wm82.a4.v1	3.24E-25	30	209	AtTET13|AT2G03840
Lcu.2RBY.7g063730.1	L.culinaris v1	4.12E-25	26	209	AtTET13|AT2G03840
Lj2g0020248.1	L.japonicus Lj1.0v1	7.38E-25	26	234	AtTET13|AT2G03840
Lj4g0018138.1	L.japonicus Lj1.0v1	1.07E-24	29	224	AtTET13|AT2G03840
arahy.Tifrunner.gnm1.ann1.XJXR2L.1	A.hypogaea v1.0	1.93E-24	28	237	AtTET13|AT2G03840
Glyma.08G012500.2.p	G.max Wm82.a4.v1	4.24E-24	30	210	AtTET13|AT2G03840
Glyma.17G084000.1.p	G.max Wm82.a4.v1	5.18E-24	27	211	AtTET13|AT2G03840
Medtr4g112570.1	M.truncatula Mt4.0v1	9.79E-24	26	203	AtTET13|AT2G03840

Lj4g0013226.1	L.japonicus Lj1.0v1	1.15E-23	29	208	AtTET13|AT2G03840
Medtr2g056130.1	M.truncatula Mt4.0v1	1.62E-23	25	232	AtTET13|AT2G03840
arahy.Tifrunner.gnm1.ann1.4R894K.1	A.hypogaea v1.0	2.61E-23	25	228	AtTET13|AT2G03840
Glyma.05G016200.1.p	G.max Wm82.a4.v1	2.83E-23	27	226	AtTET13|AT2G03840
Ca_25832	C.arietinum v1.0	1.45E-22	29	204	AtTET13|AT2G03840
Phvul.003G180300.1.p	P.vulgaris v2.1	3.38E-22	26	201	AtTET13|AT2G03840
arahy.Tifrunner.gnm1.ann1.D9WPRA.1	A.hypogaea v1.0	3.64E-22	26	227	AtTET13|AT2G03840
arahy.Tifrunner.gnm1.ann1.Z53AND.1	A.hypogaea v1.0	5.07E-22	25	228	AtTET13|AT2G03840
arahy.Tifrunner.gnm1.ann1.T23S8A.1	A.hypogaea v1.0	8.62E-22	26	229	AtTET13|AT2G03840
arahy.Tifrunner.gnm1.ann1.H32RIC.1	A.hypogaea v1.0	2.7E-21	29	234	AtTET13|AT2G03840
Phvul.004G171300.1.p	P.vulgaris v2.1	7.29E-20	26	229	AtTET13|AT2G03840
Glyma.04G144500.1.p	G.max Wm82.a4.v1	7.3E-20	25	221	AtTET13|AT2G03840
Ca_23646	C.arietinum v1.0	1.98E-19	34	151	AtTET13|AT2G03840
Glyma.15G207300.1.p	G.max Wm82.a4.v1	2.75E-19	25	221	AtTET13|AT2G03840
Phvul.009G191200.1.p	P.vulgaris v2.1	2.81E-19	29	218	AtTET13|AT2G03840
Ca_05724	C.arietinum v1.0	9.74E-19	35	136	AtTET13|AT2G03840
Lcu.2RBY.2g064540.1	L.culinaris v1	1.1E-18	26	221	AtTET13|AT2G03840
Glyma.07G129100.1.p	G.max Wm82.a4.v1	1.64E-18	25	253	AtTET13|AT2G03840
arahy.Tifrunner.gnm1.ann1.K1NV0H.1	A.hypogaea v1.0	1.2E-17	44	84	AtTET13|AT2G03840
Lj1g0003076.1	L.japonicus Lj1.0v1	1.27E-16	25	247	AtTET13|AT2G03840
arahy.Tifrunner.gnm1.ann1.QC8A1V.1	A.hypogaea v1.0	1.81E-16	23	230	AtTET13|AT2G03840
arahy.Tifrunner.gnm1.ann1.EEBB9D.1	A.hypogaea v1.0	1.81E-16	23	230	AtTET13|AT2G03840
arahy.Tifrunner.gnm1.ann1.8RU08H.1	A.hypogaea v1.0	5.53E-14	26	179	AtTET13|AT2G03840
arahy.Tifrunner.gnm1.ann1.Z53AND.1	A.hypogaea v1.0	2.46E-04	28	97	AtTET14|AT2G01960
arahy.Tifrunner.gnm1.ann1.T23S8A.1	A.hypogaea v1.0	3.61E-04	28	98	AtTET14|AT2G01960
Lj3g0001285.1	L.japonicus Lj1.0v1	5.9E-04	25	240	AtTET14|AT2G01960
Glyma.12G072100.1.p	G.max Wm82.a4.v1	8.71E-04	25	244	AtTET14|AT2G01960
Medtr4g061010.1	M.truncatula Mt4.0v1	1.91E-03	24	241	AtTET14|AT2G01960
Glyma.11G152124.1.p	G.max Wm82.a4.v1	2.9E-03	24	241	AtTET14|AT2G01960
arahy.Tifrunner.gnm1.ann1.PNW690.1	A.hypogaea v1.0	3.32E-03	28	99	AtTET14|AT2G01960
arahy.Tifrunner.gnm1.ann1.K1NV0H.1	A.hypogaea v1.0	6.14E-03	28	103	AtTET14|AT2G01960
arahy.Tifrunner.gnm1.ann1.B859HK.1	A.hypogaea v1.0	1.33E-02	28	81	AtTET14|AT2G01960
Phvul.005G137500.1.p	P.vulgaris v2.1	1.33E-02	24	254	AtTET14|AT2G01960
Ca_14606	C.arietinum v1.0	1.42E-02	23	241	AtTET14|AT2G01960
Lcu.2RBY.4g032690.1	L.culinaris v1	2.8E-02	24	240	AtTET14|AT2G01960
Phvul.011G072100.1.p	P.vulgaris v2.1	3.42E-02	23	241	AtTET14|AT2G01960
Lcu.2RBY.5g015480.1	L.culinaris v1	3.56E-02	27	91	AtTET14|AT2G01960

Glyma.09G205400.1.p	G.max Wm82.a4.v1	3.56E-02	27	91	AtTET14|AT2G01960
Medtr5g030070.1	M.truncatula Mt4.0v1	3.79E-02	26	91	AtTET14|AT2G01960
Ca_11504	C.arietinum v1.0	4.84E-02	26	95	AtTET14|AT2G01960
Medtr2g056130.1	M.truncatula Mt4.0v1	6E-02	25	105	AtTET14|AT2G01960
Glyma.13G328700.1.p	G.max Wm82.a4.v1	7.45E-02	26	171	AtTET14|AT2G01960
Lj4g0018138.1	L.japonicus Lj1.0v1	9.61E-02	26	97	AtTET14|AT2G01960
arahy.Tifrunner.gnm1.ann1.LZE6LN.1	A.hypogaea v1.0	9.84E-02	24	237	AtTET14|AT2G01960
Phvul.005G137500.1.p	P.vulgaris v2.1	4.86E-20	29	241	AtTET15|AT5G57810
Medtr4g061010.1	M.truncatula Mt4.0v1	5.48E-19	30	244	AtTET15|AT5G57810
arahy.Tifrunner.gnm1.ann1.LZE6LN.1	A.hypogaea v1.0	1.19E-18	29	239	AtTET15|AT5G57810
Phvul.011G072100.1.p	P.vulgaris v2.1	3.66E-18	30	244	AtTET15|AT5G57810
Lcu.2RBY.7g030120.1	L.culinaris v1	8.96E-18	26	246	AtTET15|AT5G57810
Glyma.05G016200.1.p	G.max Wm82.a4.v1	1.25E-17	25	240	AtTET15|AT5G57810
Glyma.11G152124.1.p	G.max Wm82.a4.v1	1.71E-17	30	244	AtTET15|AT5G57810
Glyma.17G084000.1.p	G.max Wm82.a4.v1	2.53E-17	24	238	AtTET15|AT5G57810
Glyma.16G145400.1.p	G.max Wm82.a4.v1	6.7E-17	26	238	AtTET15|AT5G57810
Ca_14606	C.arietinum v1.0	7.67E-17	28	243	AtTET15|AT5G57810
Glyma.13G328700.1.p	G.max Wm82.a4.v1	7.74E-17	32	247	AtTET15|AT5G57810
Lj3g0001285.1	L.japonicus Lj1.0v1	1.14E-16	30	244	AtTET15|AT5G57810
arahy.Tifrunner.gnm1.ann1.T23S8A.1	A.hypogaea v1.0	1.16E-16	33	128	AtTET15|AT5G57810
Lj5g0000955.1	L.japonicus Lj1.0v1	3.67E-16	28	242	AtTET15|AT5G57810
Phvul.009G191200.1.p	P.vulgaris v2.1	5.59E-16	27	245	AtTET15|AT5G57810
Lj1g0003076.1	L.japonicus Lj1.0v1	6.54E-16	27	242	AtTET15|AT5G57810
arahy.Tifrunner.gnm1.ann1.Z53AND.1	A.hypogaea v1.0	6.69E-16	26	213	AtTET15|AT5G57810
Glyma.12G072100.1.p	G.max Wm82.a4.v1	1.1E-15	28	243	AtTET15|AT5G57810
Medtr6g033335.1	M.truncatula Mt4.0v1	1.15E-15	27	237	AtTET15|AT5G57810
Phvul.009G207500.1.p	P.vulgaris v2.1	1.31E-15	24	240	AtTET15|AT5G57810
arahy.Tifrunner.gnm1.ann1.AUDB51.1	A.hypogaea v1.0	1.78E-15	30	244	AtTET15|AT5G57810
Lcu.2RBY.4g032690.1	L.culinaris v1	1.98E-15	27	244	AtTET15|AT5G57810
Glyma.04G144500.1.p	G.max Wm82.a4.v1	2.26E-15	25	249	AtTET15|AT5G57810
Glyma.02G063700.1.p	G.max Wm82.a4.v1	2.91E-15	24	237	AtTET15|AT5G57810
Lj4g0025241.1	L.japonicus Lj1.0v1	2.94E-15	27	249	AtTET15|AT5G57810
Phvul.003G180300.1.p	P.vulgaris v2.1	2.97E-15	24	240	AtTET15|AT5G57810
Lj4g0018138.1	L.japonicus Lj1.0v1	4.05E-15	32	126	AtTET15|AT5G57810
arahy.Tifrunner.gnm1.ann1.JZ8XUN.1	A.hypogaea v1.0	4.49E-15	30	243	AtTET15|AT5G57810
Glyma.15G207300.1.p	G.max Wm82.a4.v1	4.57E-15	25	249	AtTET15|AT5G57810
Lcu.2RBY.1g046720.1	L.culinaris v1	4.69E-15	25	237	AtTET15|AT5G57810

arahy.Tifrunner.gnm1.ann1.9I36N1.1	A.hypogaea v1.0	4.7E-15	26	238	AtTET15|AT5G57810
arahy.Tifrunner.gnm1.ann1.V2Z0GB.1	A.hypogaea v1.0	4.7E-15	26	238	AtTET15|AT5G57810
Phvul.004G071900.1.p	P.vulgaris v2.1	4.74E-15	26	237	AtTET15|AT5G57810
arahy.Tifrunner.gnm1.ann1.Z4U2NE.1	A.hypogaea v1.0	8.89E-15	26	242	AtTET15|AT5G57810
arahy.Tifrunner.gnm1.ann1.Z77BB1.1	A.hypogaea v1.0	1.07E-14	26	242	AtTET15|AT5G57810
Ca_08501	C.arietinum v1.0	1.12E-14	27	242	AtTET15|AT5G57810
Glyma.17G069300.1.p	G.max Wm82.a4.v1	1.46E-14	26	238	AtTET15|AT5G57810
Medtr8g066790.1	M.truncatula Mt4.0v1	1.47E-14	24	255	AtTET15|AT5G57810
Ca_06587	C.arietinum v1.0	1.51E-14	25	230	AtTET15|AT5G57810
Glyma.09G205400.1.p	G.max Wm82.a4.v1	2.56E-14	24	238	AtTET15|AT5G57810
Glyma.13G091000.1.p	G.max Wm82.a4.v1	2.91E-14	25	238	AtTET15|AT5G57810
Lcu.2RBY.7g075810.1	L.culinaris v1	3E-14	25	240	AtTET15|AT5G57810
Medtr1g095530.1	M.truncatula Mt4.0v1	3.49E-14	32	125	AtTET15|AT5G57810
Lj2g0026181.1	L.japonicus Lj1.0v1	3.58E-14	29	242	AtTET15|AT5G57810
Phvul.002G140500.1.p	P.vulgaris v2.1	3.85E-14	24	229	AtTET15|AT5G57810
arahy.Tifrunner.gnm1.ann1.8QTZ72.1	A.hypogaea v1.0	3.88E-14	25	246	AtTET15|AT5G57810
Medtr4g112570.1	M.truncatula Mt4.0v1	4.37E-14	31	126	AtTET15|AT5G57810
Glyma.20G178700.1.p	G.max Wm82.a4.v1	5.4E-14	27	242	AtTET15|AT5G57810
Lcu.2RBY.7g063730.1	L.culinaris v1	6.48E-14	30	126	AtTET15|AT5G57810
arahy.Tifrunner.gnm1.ann1.2A0H1U.1	A.hypogaea v1.0	7.58E-14	31	125	AtTET15|AT5G57810
arahy.Tifrunner.gnm1.ann1.XJXR2L.1	A.hypogaea v1.0	7.73E-14	31	125	AtTET15|AT5G57810
Glyma.10G212400.1.p	G.max Wm82.a4.v1	8.17E-14	27	242	AtTET15|AT5G57810
Phvul.002G287500.1.p	P.vulgaris v2.1	1.34E-13	30	125	AtTET15|AT5G57810
Phvul.007G093400.1.p	P.vulgaris v2.1	2.06E-13	26	242	AtTET15|AT5G57810
arahy.Tifrunner.gnm1.ann1.PNW690.1	A.hypogaea v1.0	2.65E-13	28	123	AtTET15|AT5G57810
Lcu.2RBY.2g046220.1	L.culinaris v1	2.68E-13	24	236	AtTET15|AT5G57810
Glyma.05G205600.1.p	G.max Wm82.a4.v1	2.69E-13	30	145	AtTET15|AT5G57810
Glyma.08G012500.2.p	G.max Wm82.a4.v1	3.05E-13	30	122	AtTET15|AT5G57810
Glyma.01G095700.1.p	G.max Wm82.a4.v1	3.11E-13	26	250	AtTET15|AT5G57810
arahy.Tifrunner.gnm1.ann1.B859HK.1	A.hypogaea v1.0	4.04E-13	28	123	AtTET15|AT5G57810
arahy.Tifrunner.gnm1.ann1.H32RIC.1	A.hypogaea v1.0	4.21E-13	26	218	AtTET15|AT5G57810
Lcu.2RBY.4g075230.1	L.culinaris v1	4.84E-13	29	125	AtTET15|AT5G57810
Ca_05724	C.arietinum v1.0	7.21E-13	25	224	AtTET15|AT5G57810
Medtr8g097280.1	M.truncatula Mt4.0v1	8.77E-13	29	125	AtTET15|AT5G57810
Lj4g0013226.1	L.japonicus Lj1.0v1	1.38E-12	29	125	AtTET15|AT5G57810
Lj4g0026647.1	L.japonicus Lj1.0v1	1.54E-12	25	251	AtTET15|AT5G57810
Phvul.002G304100.1.p	P.vulgaris v2.1	2.15E-12	26	125	AtTET15|AT5G57810

Phvul.003G284700.1.p	P.vulgaris v2.1	2.92E-12	27	126	AtTET15|AT5G57810
Glyma.08G029100.1.p	G.max Wm82.a4.v1	4.08E-12	26	144	AtTET15|AT5G57810
arahy.Tifrunner.gnm1.ann1.EM0Y56.1	A.hypogaea v1.0	6.04E-12	28	123	AtTET15|AT5G57810
arahy.Tifrunner.gnm1.ann1.N28W6S.1	A.hypogaea v1.0	6.28E-12	28	123	AtTET15|AT5G57810
Lj2g0015394.1	L.japonicus Lj1.0v1	7.81E-12	27	113	AtTET15|AT5G57810
Ca_11504	C.arietinum v1.0	9.54E-12	23	235	AtTET15|AT5G57810
Lcu.2RBY.2g064540.1	L.culinaris v1	1.05E-11	25	244	AtTET15|AT5G57810
Medtr2g056130.1	M.truncatula Mt4.0v1	1.15E-11	33	126	AtTET15|AT5G57810
Phvul.003G151800.1.p	P.vulgaris v2.1	1.31E-11	24	238	AtTET15|AT5G57810
Ca_23646	C.arietinum v1.0	1.68E-11	30	125	AtTET15|AT5G57810
Medtr5g030070.1	M.truncatula Mt4.0v1	2.1E-11	29	113	AtTET15|AT5G57810
Phvul.004G171300.1.p	P.vulgaris v2.1	2.25E-11	28	123	AtTET15|AT5G57810
Lj2g0020248.1	L.japonicus Lj1.0v1	2.59E-11	27	133	AtTET15|AT5G57810
Glyma.04G147100.1.p	G.max Wm82.a4.v1	2.69E-11	25	227	AtTET15|AT5G57810
Lcu.2RBY.5g015480.1	L.culinaris v1	3.02E-11	27	113	AtTET15|AT5G57810
arahy.Tifrunner.gnm1.ann1.0ZGE19.1	A.hypogaea v1.0	3.1E-11	27	240	AtTET15|AT5G57810
Glyma.07G129100.1.p	G.max Wm82.a4.v1	3.14E-11	31	117	AtTET15|AT5G57810
arahy.Tifrunner.gnm1.ann1.A2QH6A.1	A.hypogaea v1.0	3.17E-11	24	245	AtTET15|AT5G57810
Medtr4g129670.1	M.truncatula Mt4.0v1	5.92E-11	24	240	AtTET15|AT5G57810
Ca_25832	C.arietinum v1.0	6.13E-11	22	216	AtTET15|AT5G57810
Lcu.2RBY.4g078330.1	L.culinaris v1	6.89E-11	27	125	AtTET15|AT5G57810
Ca_05887	C.arietinum v1.0	7.43E-11	28	123	AtTET15|AT5G57810
Medtr8g101600.1	M.truncatula Mt4.0v1	4.03E-10	26	132	AtTET15|AT5G57810
arahy.Tifrunner.gnm1.ann1.4R894K.1	A.hypogaea v1.0	4.03E-10	26	242	AtTET15|AT5G57810
Lj4g0027264.1	L.japonicus Lj1.0v1	4.3E-10	28	123	AtTET15|AT5G57810
arahy.Tifrunner.gnm1.ann1.8RU08H.1	A.hypogaea v1.0	4.04E-09	25	218	AtTET15|AT5G57810
arahy.Tifrunner.gnm1.ann1.K1NV0H.1	A.hypogaea v1.0	1.12E-08	30	83	AtTET15|AT5G57810
Lj6g0020177.1	L.japonicus Lj1.0v1	4.11E-08	23	228	AtTET15|AT5G57810
Ca_03295	C.arietinum v1.0	5.73E-08	28	112	AtTET15|AT5G57810
arahy.Tifrunner.gnm1.ann1.D9WPRA.1	A.hypogaea v1.0	1.45E-07	25	242	AtTET15|AT5G57810
arahy.Tifrunner.gnm1.ann1.QC8A1V.1	A.hypogaea v1.0	2.1E-07	35	78	AtTET15|AT5G57810
arahy.Tifrunner.gnm1.ann1.8QTZ72.1	A.hypogaea v1.0	2.85E-09	21	239	AtTET16|AT1G18510
arahy.Tifrunner.gnm1.ann1.A2QH6A.1	A.hypogaea v1.0	2.07E-08	22	238	AtTET16|AT1G18510
Glyma.02G063700.1.p	G.max Wm82.a4.v1	2.71E-07	21	232	AtTET16|AT1G18510
Glyma.10G212400.1.p	G.max Wm82.a4.v1	2.95E-07	23	241	AtTET16|AT1G18510
Lcu.2RBY.7g075810.1	L.culinaris v1	1.32E-06	21	248	AtTET16|AT1G18510
Glyma.16G145400.1.p	G.max Wm82.a4.v1	1.74E-06	21	230	AtTET16|AT1G18510

Glyma.04G147100.1.p	G.max Wm82.a4.v1	5.29E-06	22	237	AtTET16|AT1G18510
Glyma.20G178700.1.p	G.max Wm82.a4.v1	5.35E-06	22	241	AtTET16|AT1G18510
Lj5g0000955.1	L.japonicus Lj1.0v1	5.5E-06	23	236	AtTET16|AT1G18510
Phvul.007G093400.1.p	P.vulgaris v2.1	6.75E-06	22	241	AtTET16|AT1G18510
Lj2g0026181.1	L.japonicus Lj1.0v1	1.8E-05	20	229	AtTET16|AT1G18510
Phvul.004G071900.1.p	P.vulgaris v2.1	1.92E-05	20	226	AtTET16|AT1G18510
Ca_08501	C.arietinum v1.0	6.61E-05	22	241	AtTET16|AT1G18510
Medtr6g033335.1	M.truncatula Mt4.0v1	9.74E-05	19	218	AtTET16|AT1G18510
Glyma.13G328700.1.p	G.max Wm82.a4.v1	1.73E-04	20	245	AtTET16|AT1G18510
arahy.Tifrunner.gnm1.ann1.Z77BB1.1	A.hypogaea v1.0	2.26E-04	22	231	AtTET16|AT1G18510
Medtr4g129670.1	M.truncatula Mt4.0v1	2.3E-04	21	238	AtTET16|AT1G18510
arahy.Tifrunner.gnm1.ann1.Z4U2NE.1	A.hypogaea v1.0	2.46E-04	22	231	AtTET16|AT1G18510
Ca_03295	C.arietinum v1.0	8.36E-04	21	209	AtTET16|AT1G18510
Lcu.2RBY.1g046720.1	L.culinaris v1	8.65E-04	22	241	AtTET16|AT1G18510
Lj6g0020177.1	L.japonicus Lj1.0v1	1.1E-03	20	230	AtTET16|AT1G18510
Lj4g0025241.1	L.japonicus Lj1.0v1	1.18E-03	18	234	AtTET16|AT1G18510
Lcu.2RBY.2g046220.1	L.culinaris v1	2.08E-03	18	218	AtTET16|AT1G18510
arahy.Tifrunner.gnm1.ann1.LZE6LN.1	A.hypogaea v1.0	2.53E-03	20	271	AtTET16|AT1G18510
arahy.Tifrunner.gnm1.ann1.0ZGE19.1	A.hypogaea v1.0	3.42E-03	19	254	AtTET16|AT1G18510
Lcu.2RBY.4g032690.1	L.culinaris v1	4.38E-03	19	232	AtTET16|AT1G18510
Ca_14606	C.arietinum v1.0	6.66E-03	19	236	AtTET16|AT1G18510
Phvul.009G191200.1.p	P.vulgaris v2.1	1.75E-02	20	240	AtTET16|AT1G18510
Medtr1g095530.1	M.truncatula Mt4.0v1	2.48E-02	22	222	AtTET16|AT1G18510
arahy.Tifrunner.gnm1.ann1.4R894K.1	A.hypogaea v1.0	2.83E-02	19	214	AtTET16|AT1G18510
arahy.Tifrunner.gnm1.ann1.D9WPRA.1	A.hypogaea v1.0	7.84E-02	21	222	AtTET16|AT1G18510
Glyma.17G084000.1.p	G.max Wm82.a4.v1	1.57E-03	25	120	AtTET17|AT1G74045
Lcu.2RBY.7g063730.1	L.culinaris v1	3.1E-03	23	137	AtTET17|AT1G74045
Glyma.05G016200.1.p	G.max Wm82.a4.v1	4.37E-03	25	120	AtTET17|AT1G74045
Lcu.2RBY.2g064540.1	L.culinaris v1	1.13E-02	25	120	AtTET17|AT1G74045
Ca_06587	C.arietinum v1.0	1.34E-02	25	126	AtTET17|AT1G74045
Lj4g0018138.1	L.japonicus Lj1.0v1	1.43E-02	23	124	AtTET17|AT1G74045
Medtr4g112570.1	M.truncatula Mt4.0v1	1.91E-02	24	129	AtTET17|AT1G74045
arahy.Tifrunner.gnm1.ann1.H32RIC.1	A.hypogaea v1.0	2.18E-02	22	128	AtTET17|AT1G74045
Phvul.009G191200.1.p	P.vulgaris v2.1	2.99E-02	25	128	AtTET17|AT1G74045
Lj1g0003076.1	L.japonicus Lj1.0v1	3.96E-02	30	79	AtTET17|AT1G74045
Glyma.05G205600.1.p	G.max Wm82.a4.v1	4.5E-02	21	124	AtTET17|AT1G74045
Medtr2g056130.1	M.truncatula Mt4.0v1	5.98E-02	23	117	AtTET17|AT1G74045
