# Supplementary figures and images for: TETRASPANIN 8-1 from Phaseolus vulgaris plays a key role during mutualistic interactions
Source: Front Plant Sci. 2023 Jul 3;14:1152493. doi: 10.3389/fpls.2023.1152493 (PMC10352089; doi:10.3389/fpls.2023.1152493)

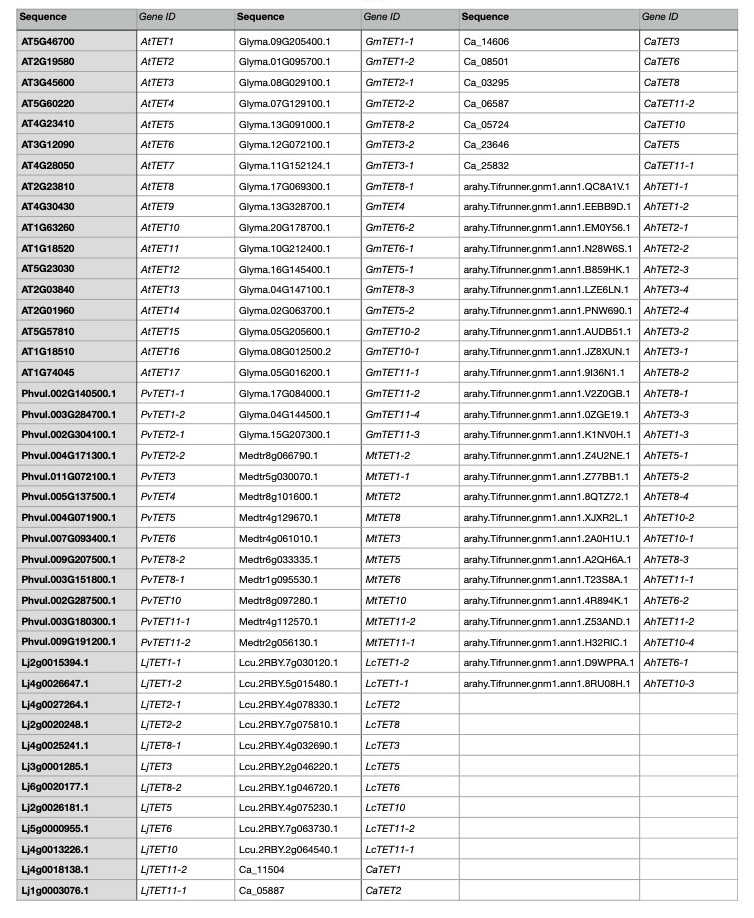

Supplement: Supplementary file 1 [file DataSheet_1.zip › Supplementary Figure 1.JPEG]

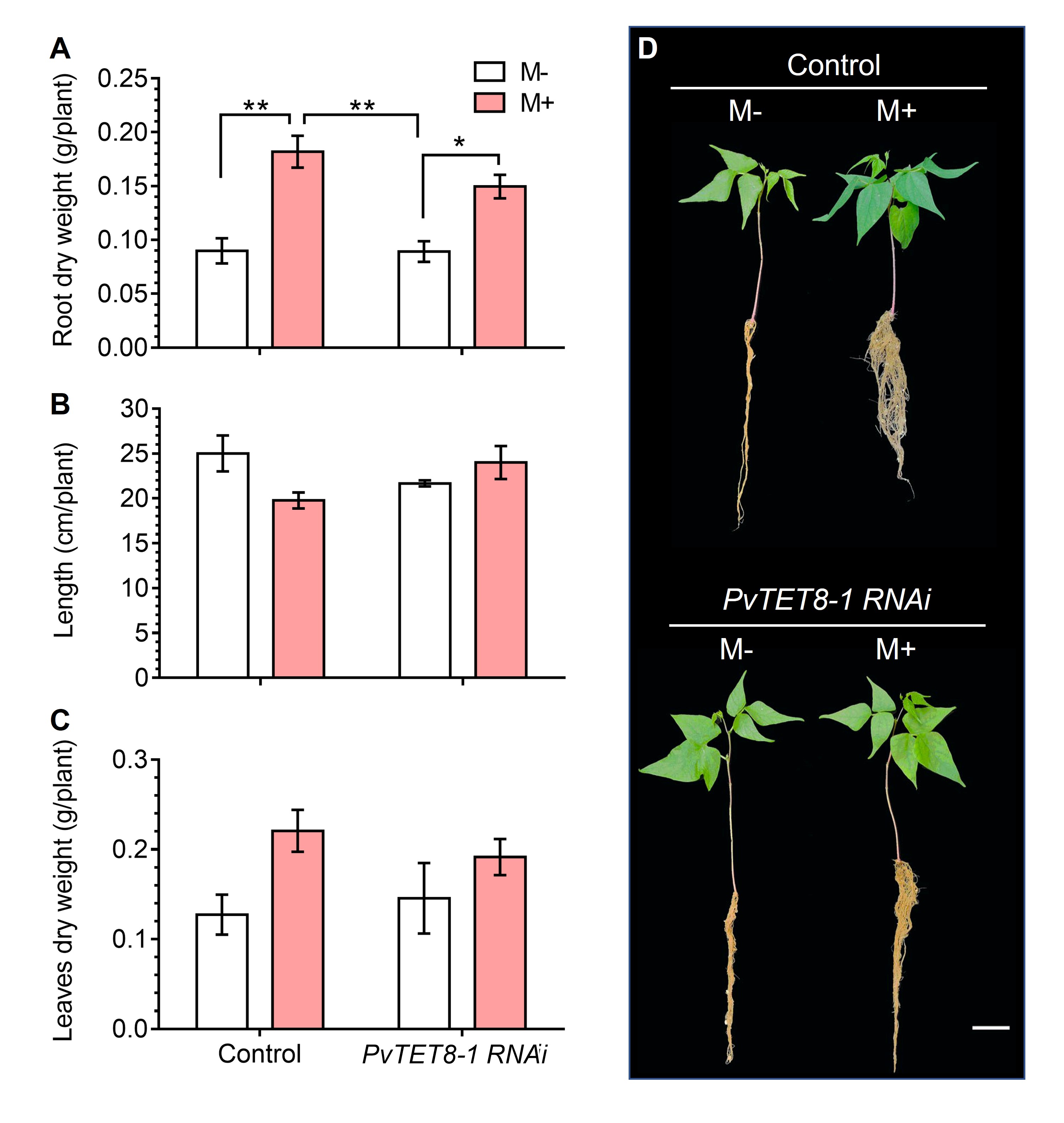

Supplement: Supplementary file 1 [file DataSheet_1.zip › Supplementary Figure 10.JPEG]

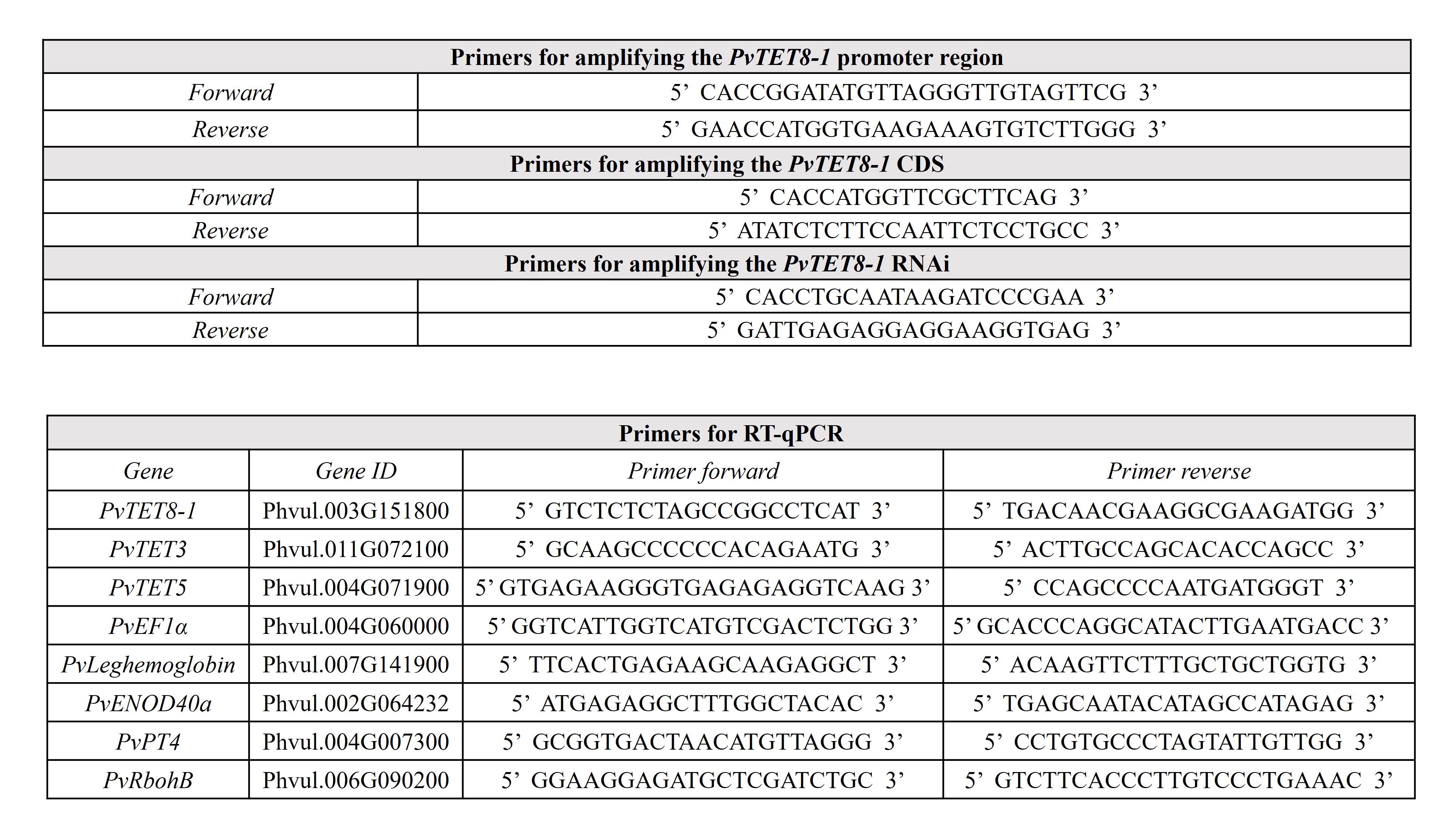

Supplement: Supplementary file 1 [file DataSheet_1.zip › Supplementary Figure 11.JPEG]

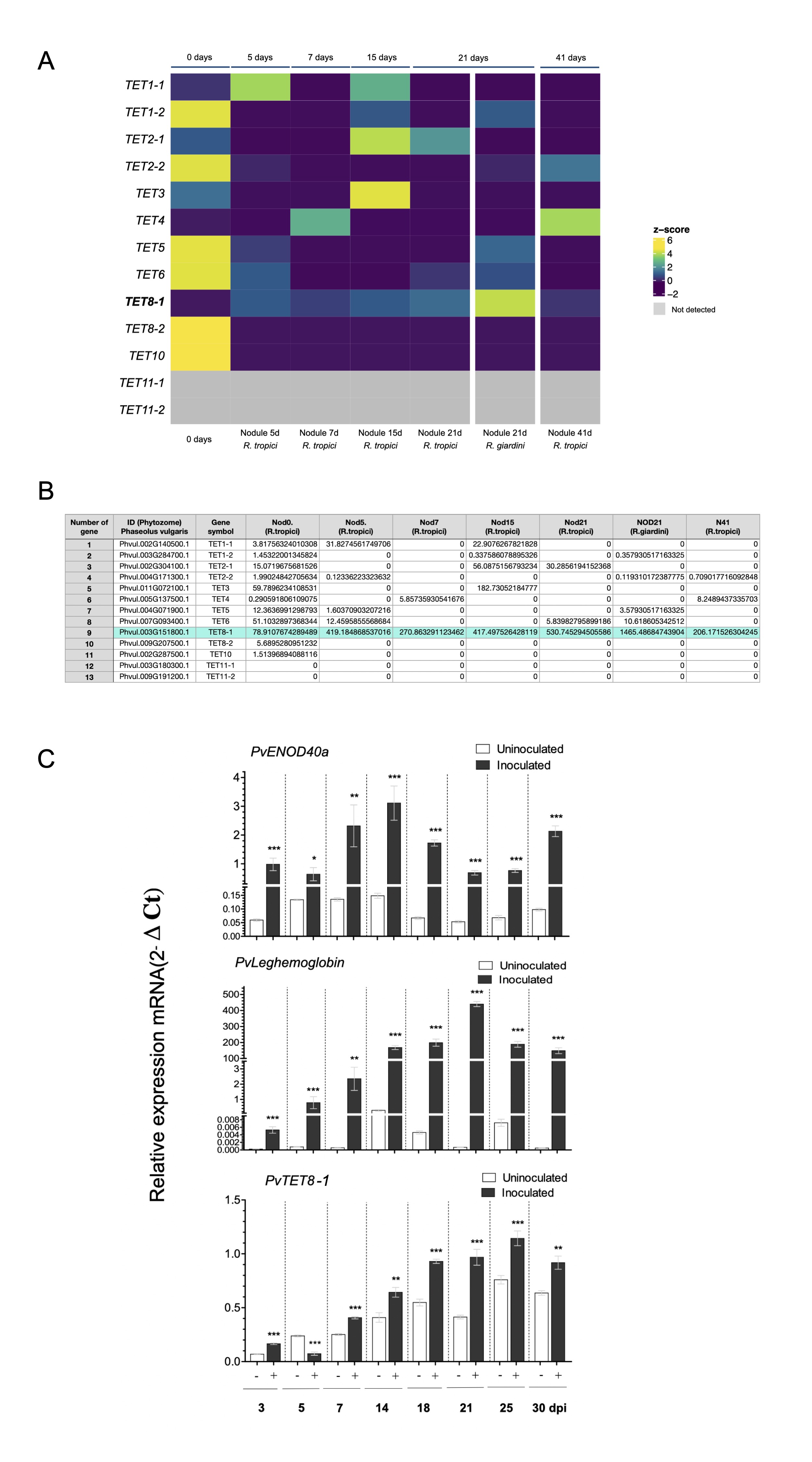

Supplement: Supplementary file 1 [file DataSheet_1.zip › Supplementary Figure 2.JPEG]

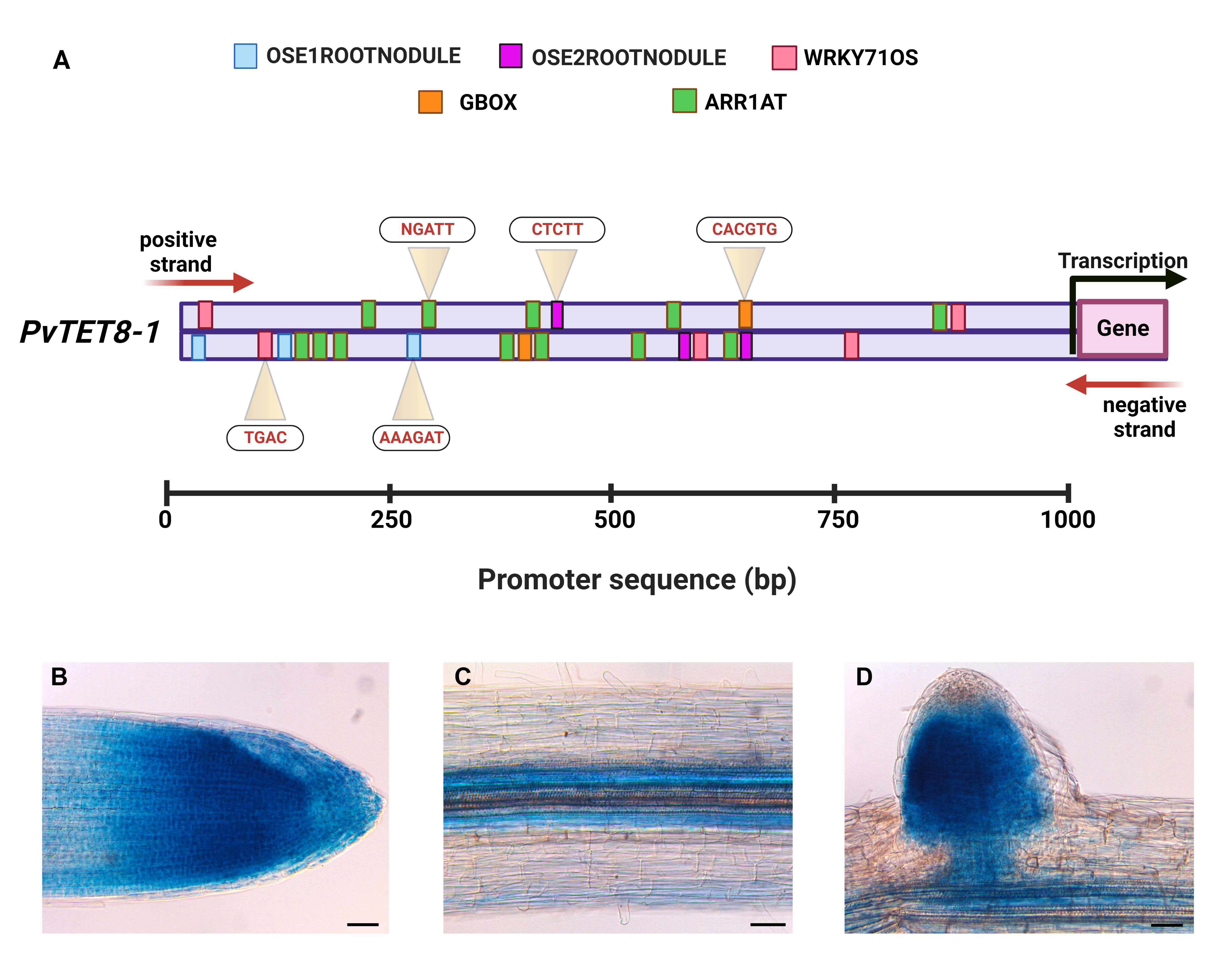

Supplement: Supplementary file 1 [file DataSheet_1.zip › Supplementary Figure 3.JPEG]

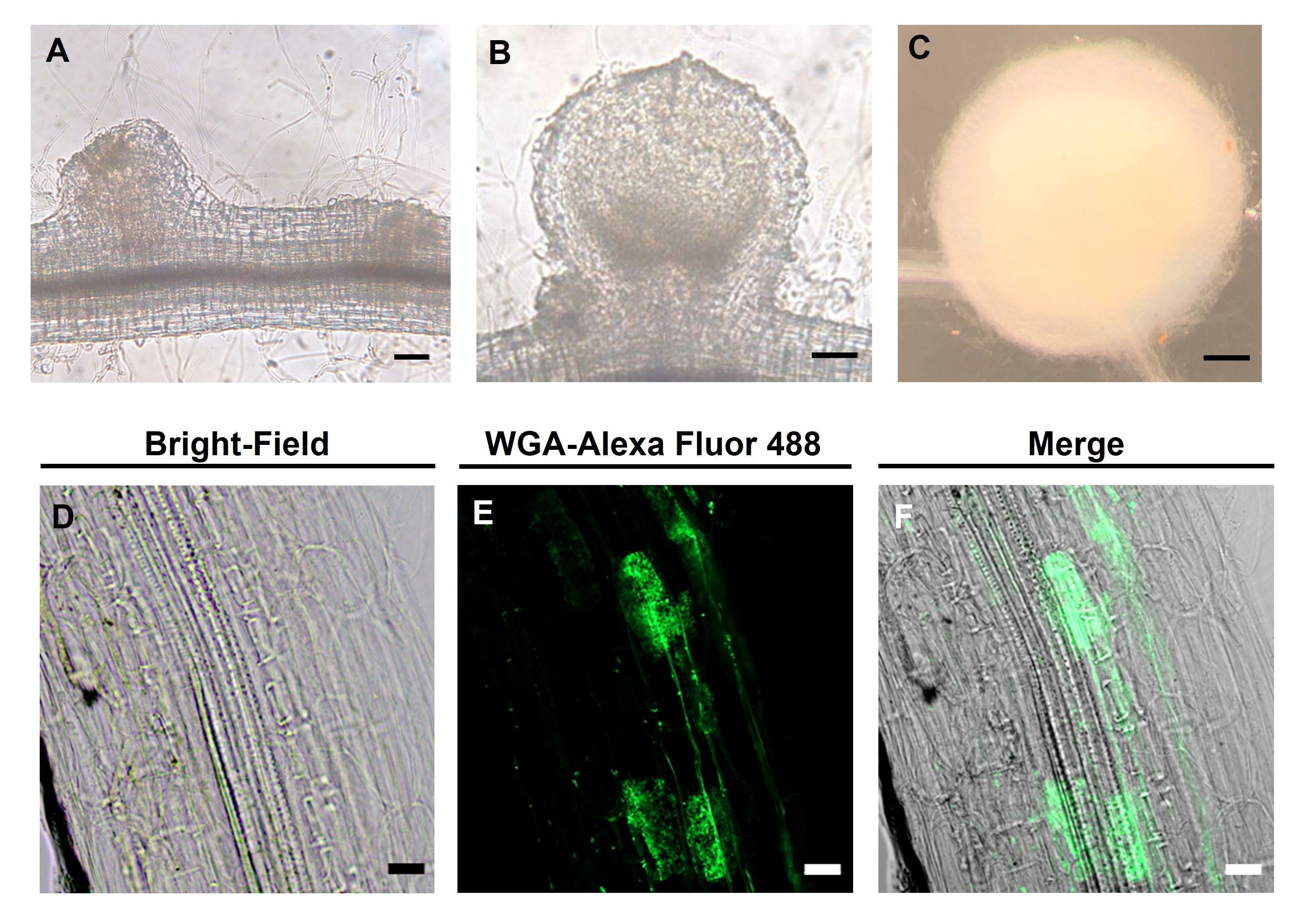

Supplement: Supplementary file 1 [file DataSheet_1.zip › Supplementary Figure 4.JPEG]

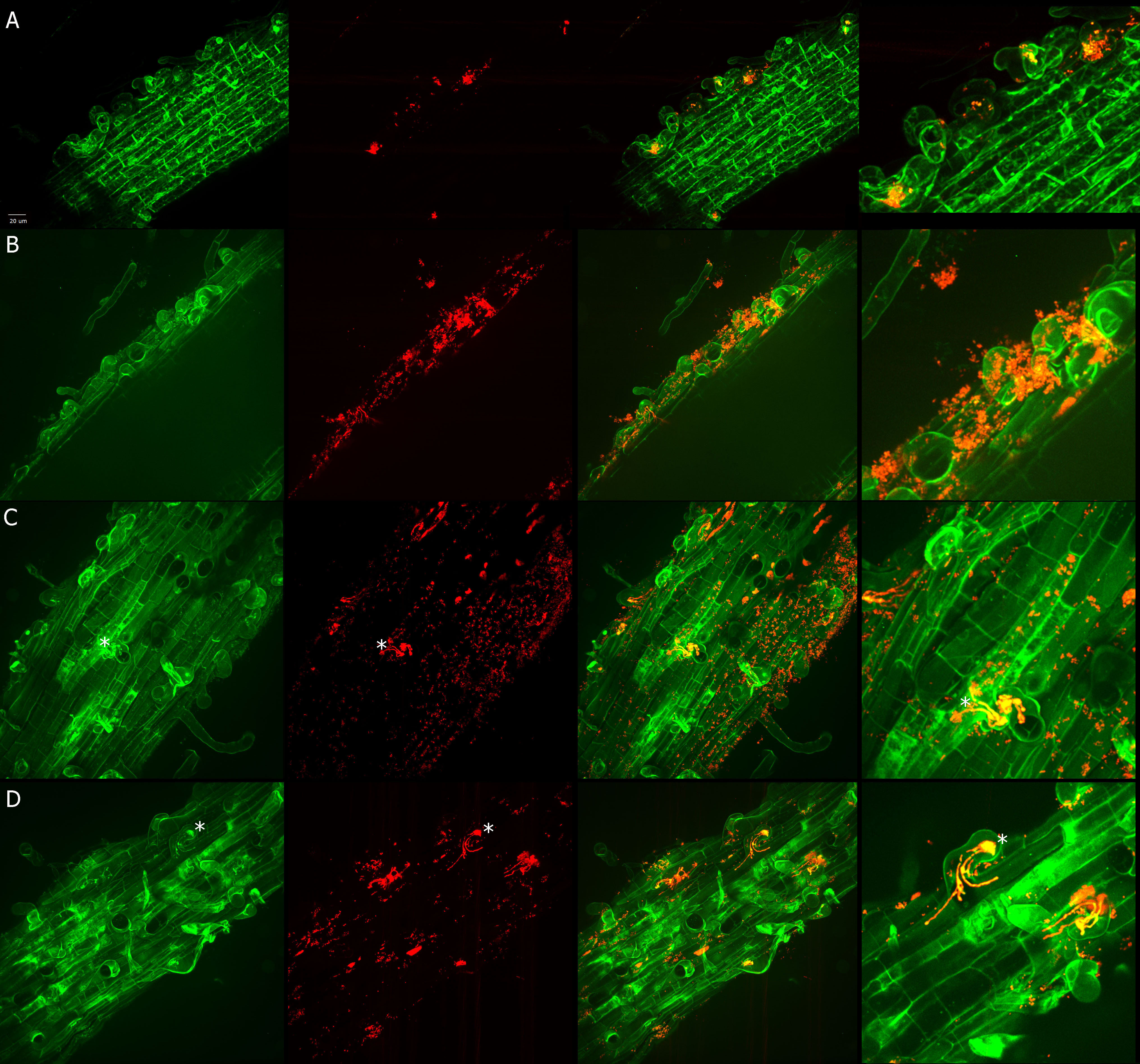

Supplement: Supplementary file 1 [file DataSheet_1.zip › Supplementary Figure 5.JPEG]

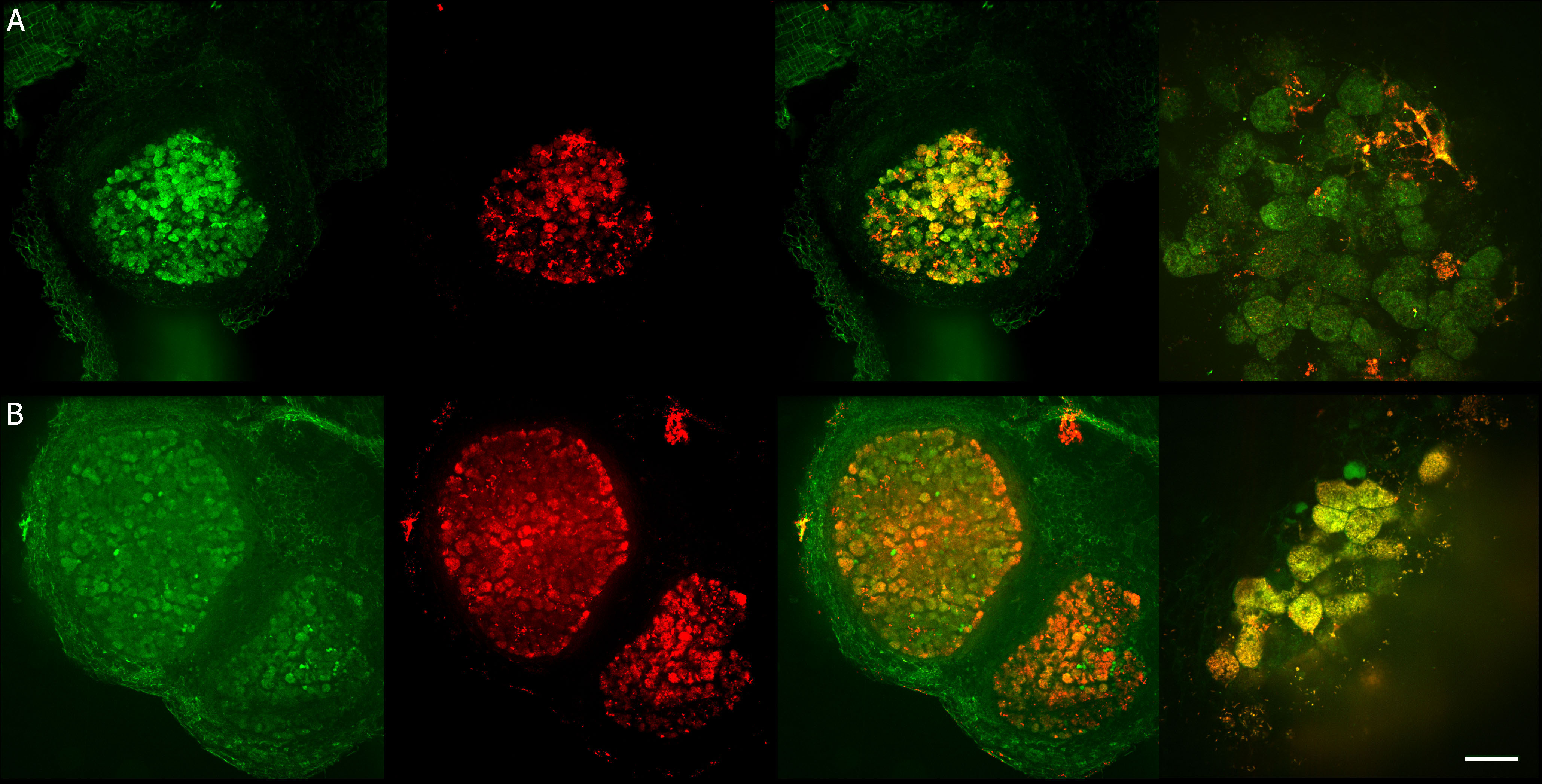

Supplement: Supplementary file 1 [file DataSheet_1.zip › Supplementary Figure 6.JPEG]

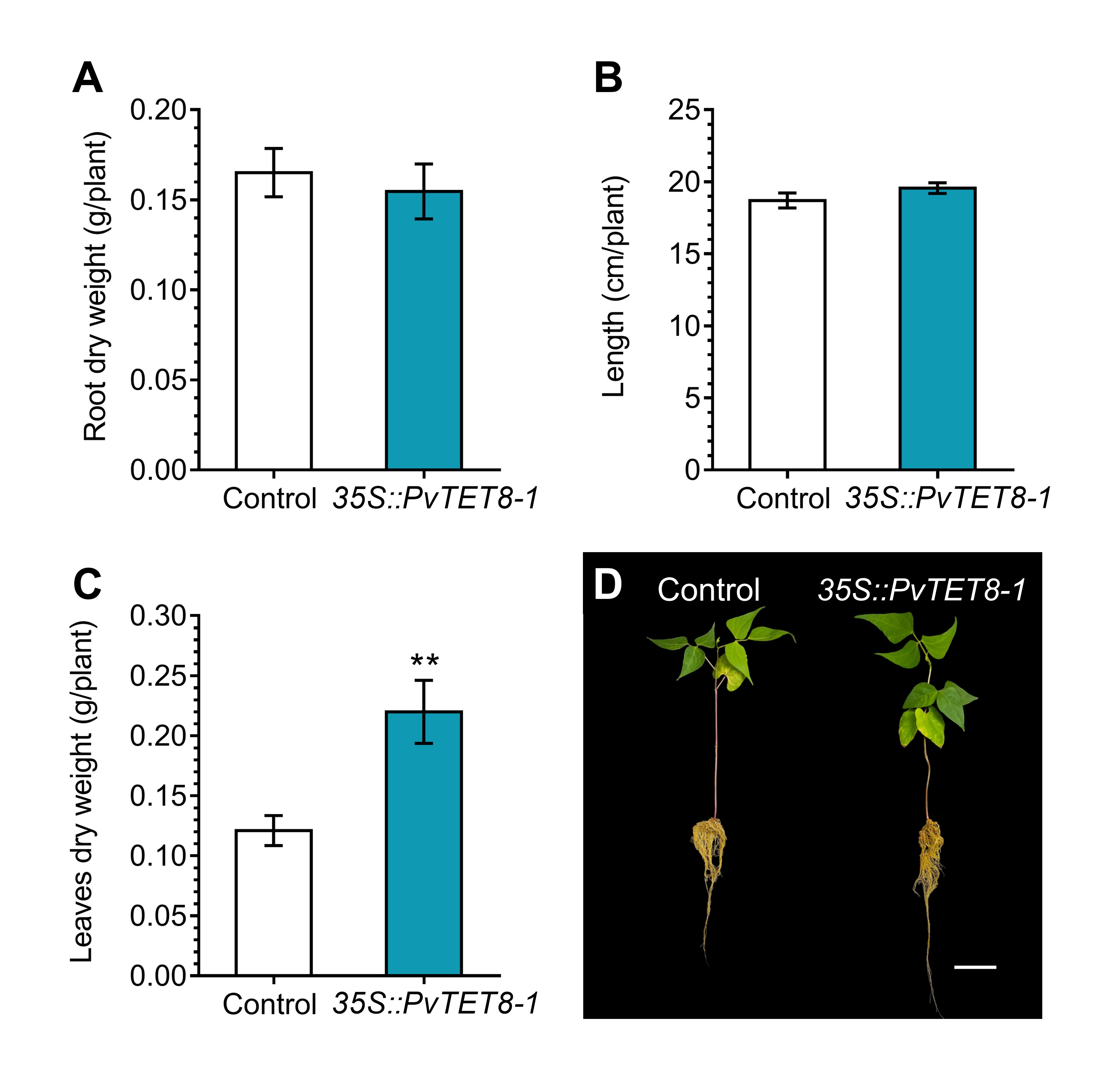

Supplement: Supplementary file 1 [file DataSheet_1.zip › Supplementary Figure 7.JPEG]

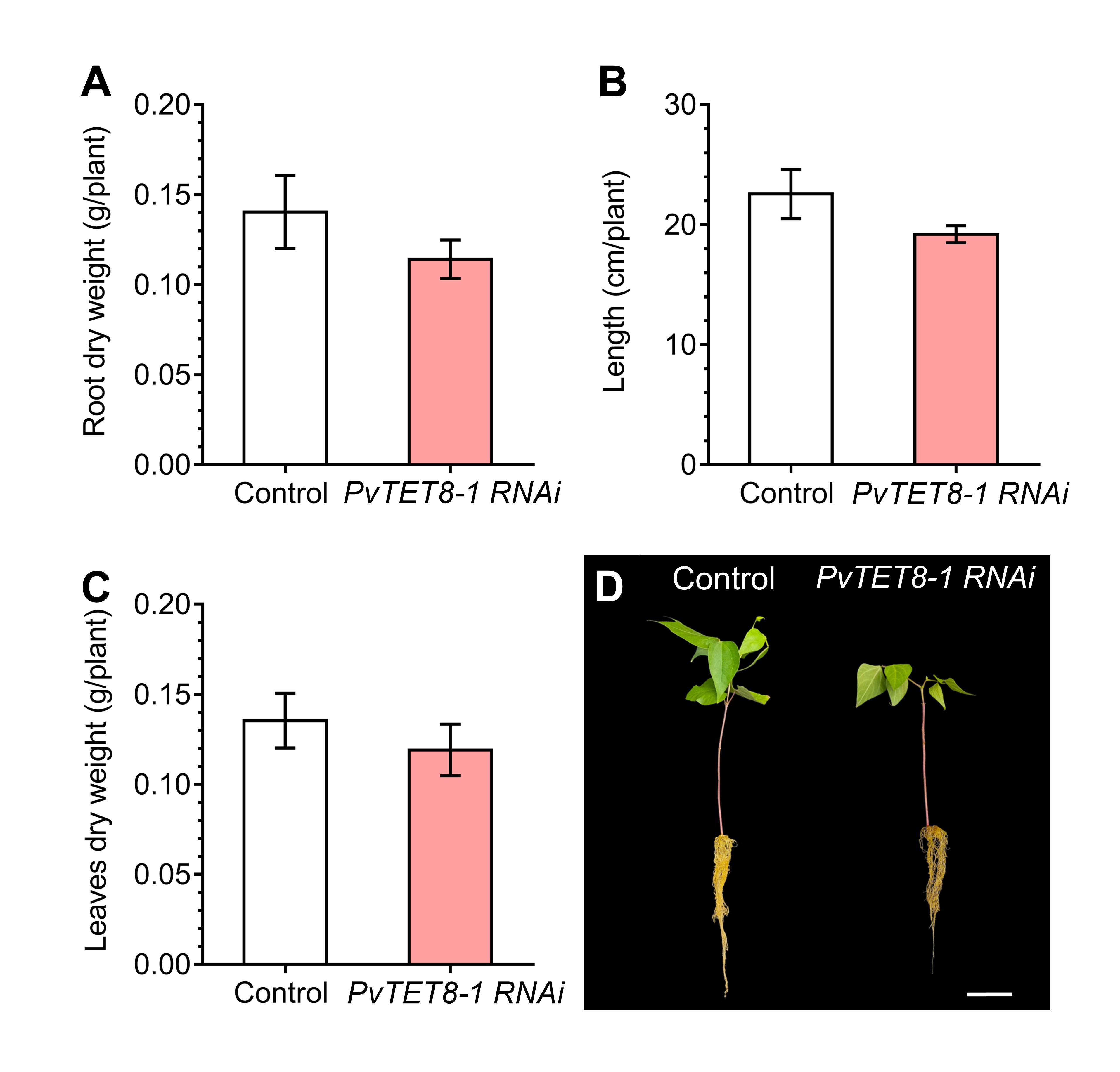

Supplement: Supplementary file 1 [file DataSheet_1.zip › Supplementary Figure 8.JPEG]

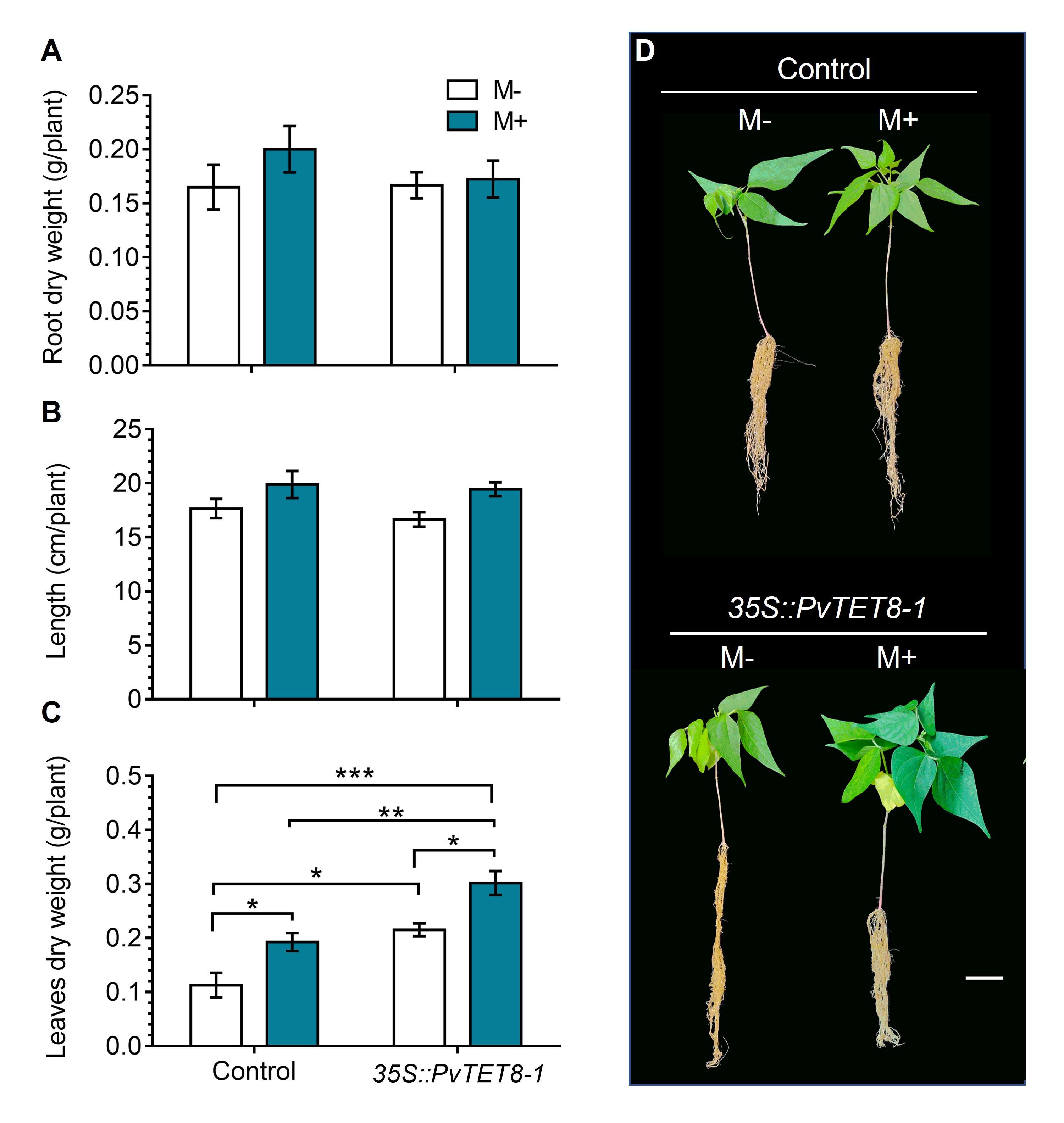

Supplement: Supplementary file 1 [file DataSheet_1.zip › Supplementary Figure 9.JPEG]
